# Supplementary material for: epiAneufinder identifies copy number alterations from single-cell ATAC-seq data
Source: Nat Commun. 2023 Sep 20;14:5846. doi: 10.1038/s41467-023-41076-1 (PMC10511508; doi:10.1038/s41467-023-41076-1)
Supplement: Supplementary file 1 — Supplementary information [file 41467_2023_41076_MOESM1_ESM.pdf]

Supplementary Figures and Tables:

epiAneufinder identifyies copy number alterations from single-cell ATAC-seq data

Akshaya Ramakrishnan[*1], Aikaterini Symeonidi[*,1,2,4], Patrick Hanel[1,2], Katharina T. Schmid[2], Maria L. Richter[2], Michael Schubert[3], Maria Colomé-Tatché[1,2,4]

[1] Institute of Computational Biology, Helmholtz Zentrum München, German Research Center for Environmental Health, Neuherberg, Germany.
[2] Biomedical Center (BMC), Physiological Chemistry, Faculty of Medicine, LMU Munich, Planegg-Martinsried, Germany.
[3] Oncode Institute, Division of Cell Biology, Netherlands Cancer Institute, Plesmanlaan 121, 1066 CX, Amsterdam, the Netherlands.
[*] These authors contributed equally: Akshaya Ramakrishnan and Aikaterini Symeonidi.
[4] Correspondence to aikaterini.symeonidi@helmholtz-munich.de
maria.colome@bmc.med.lmu.de

Figure S1: Number of reads distribution per somy

a)

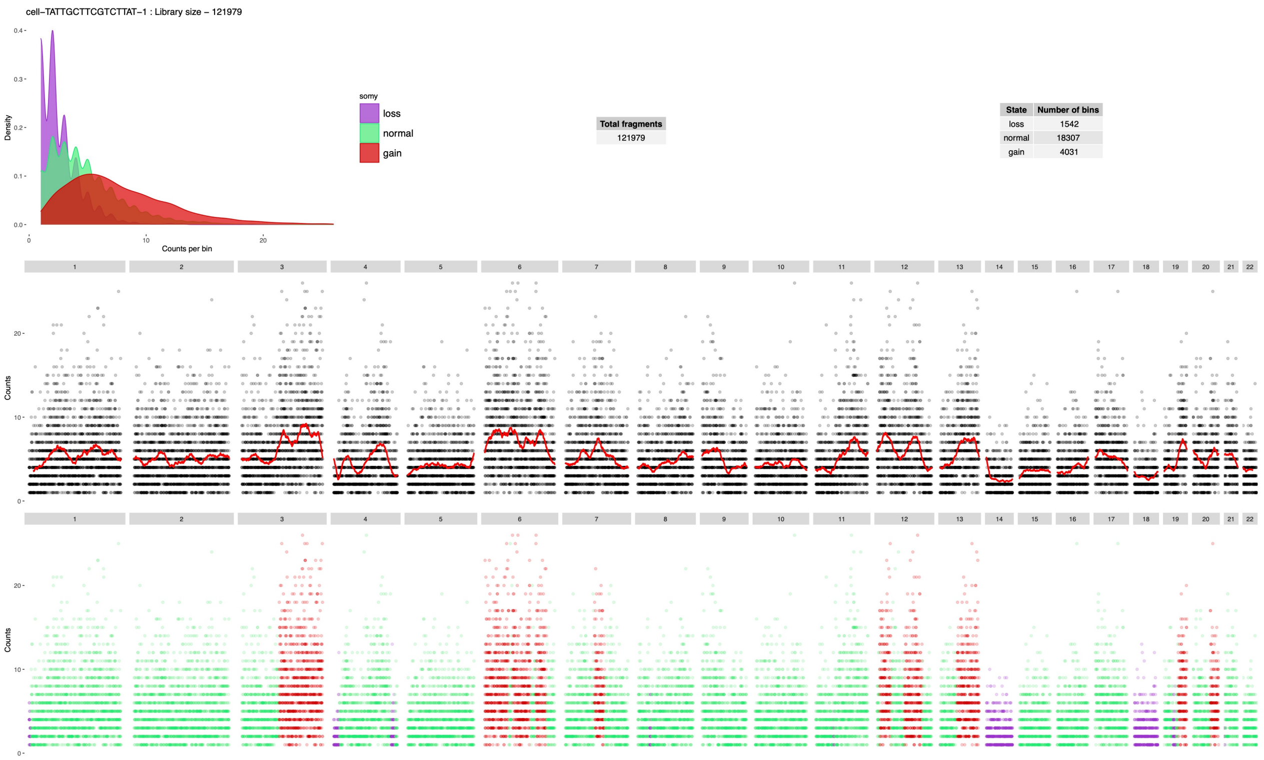

b)

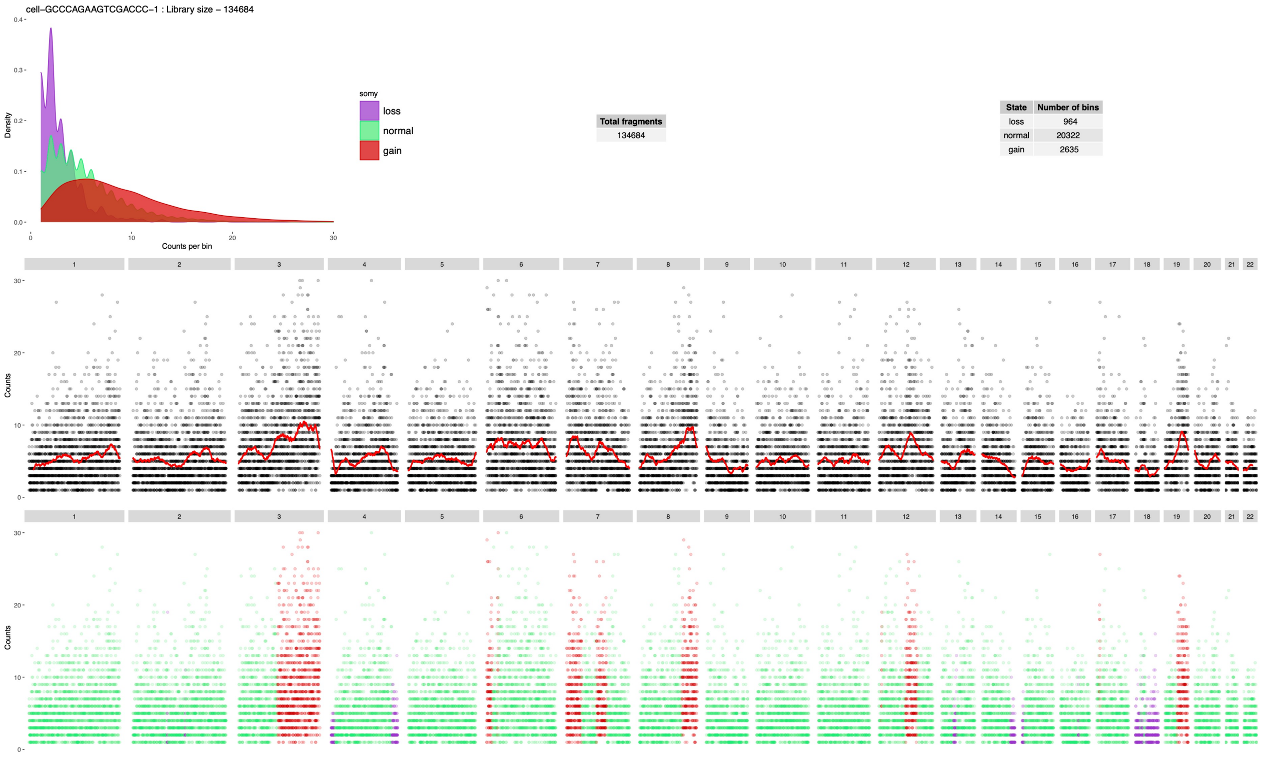

Figure S1: Number of reads distribution per somy

c)

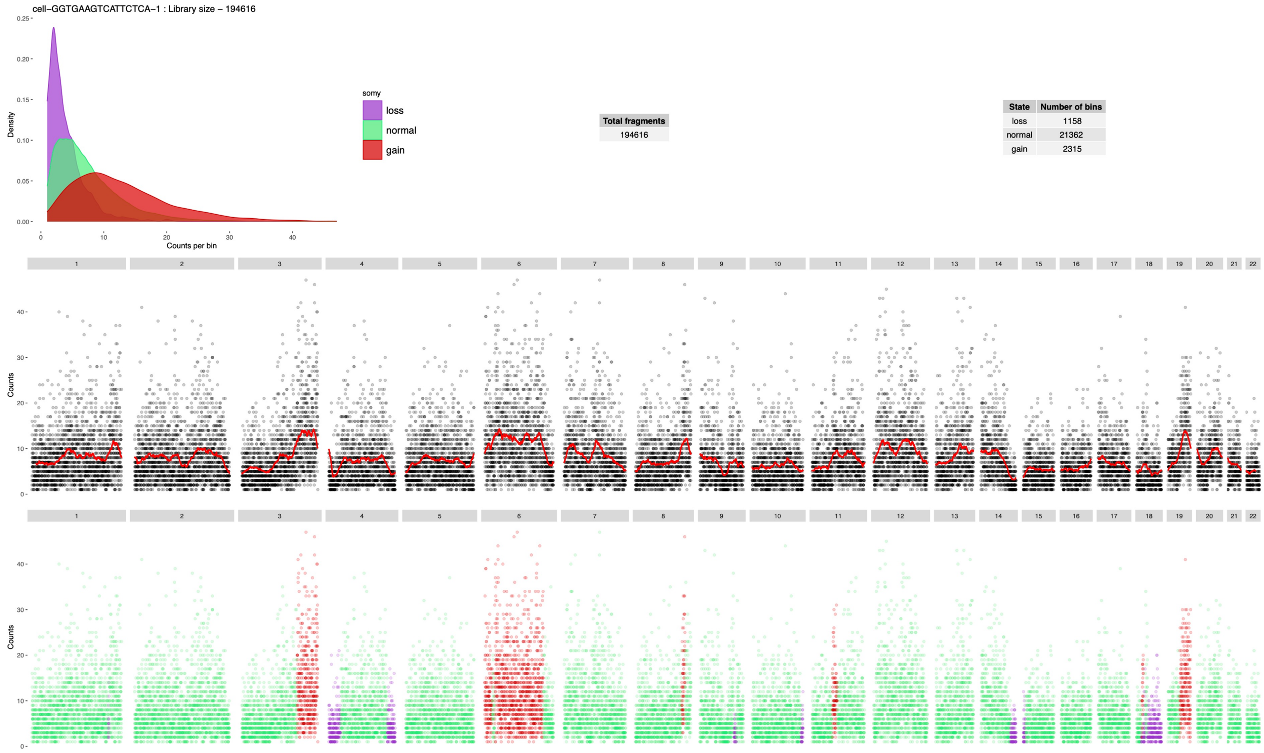

d)

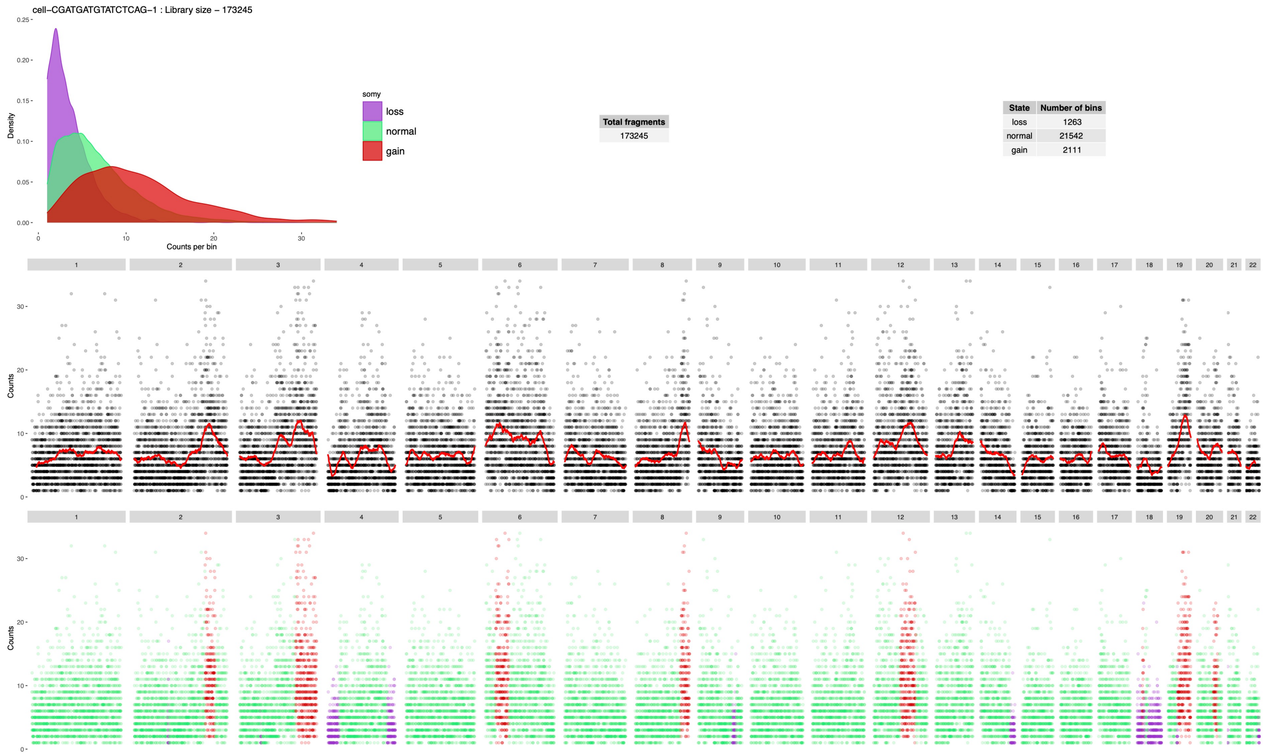

Figure S1: Number of reads distribution per somy

e)

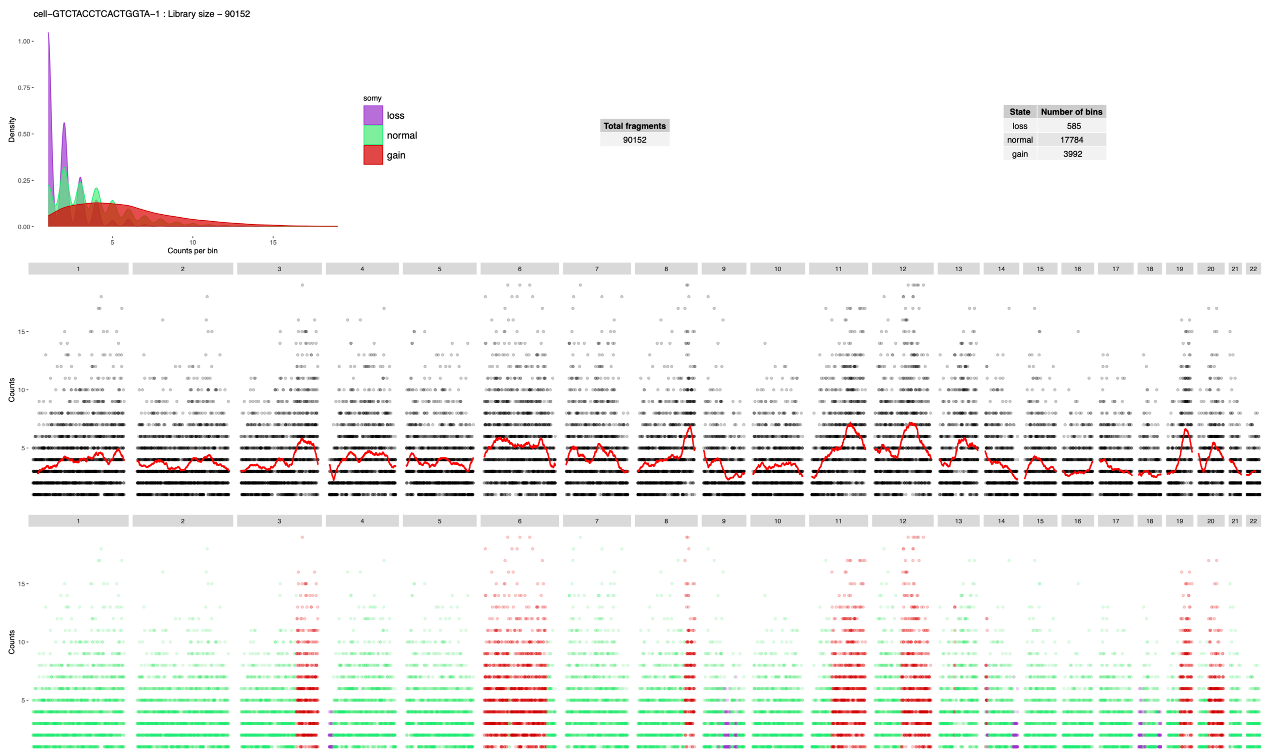

f)

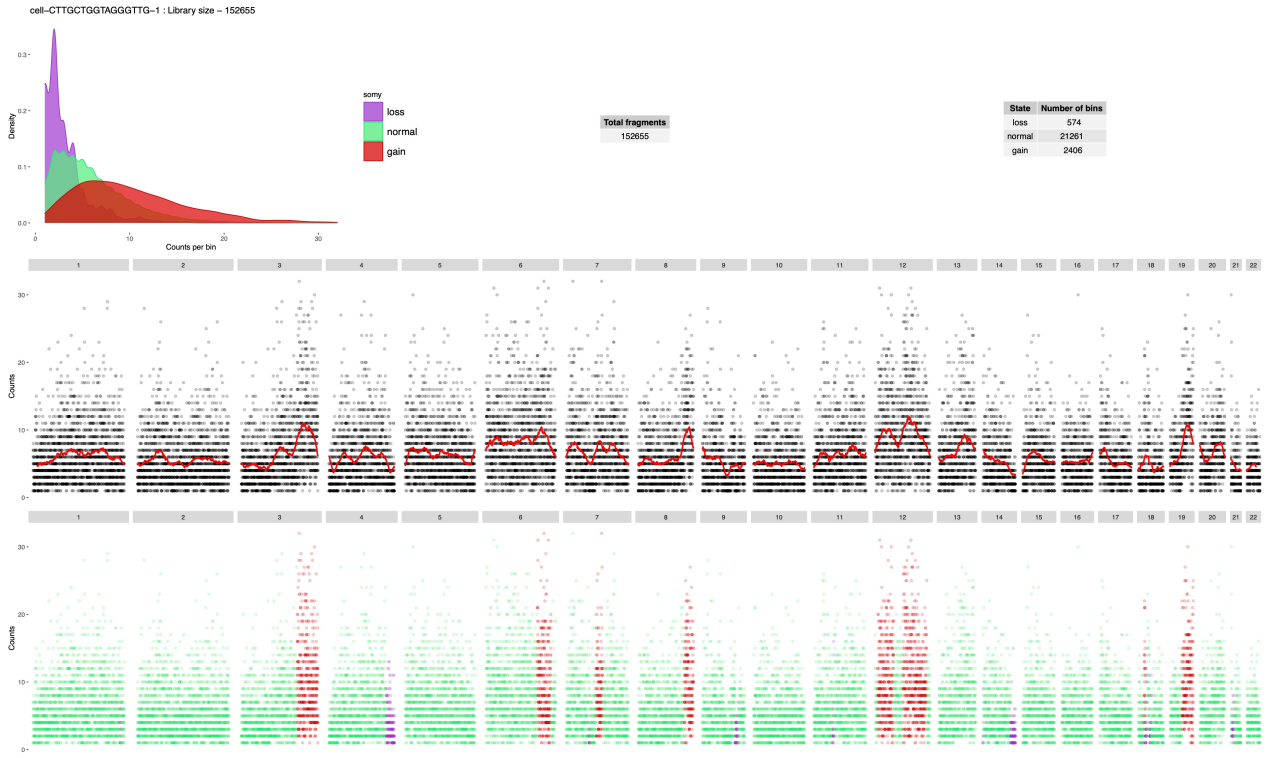

Figure S1: Number of reads distribution per somy

g)

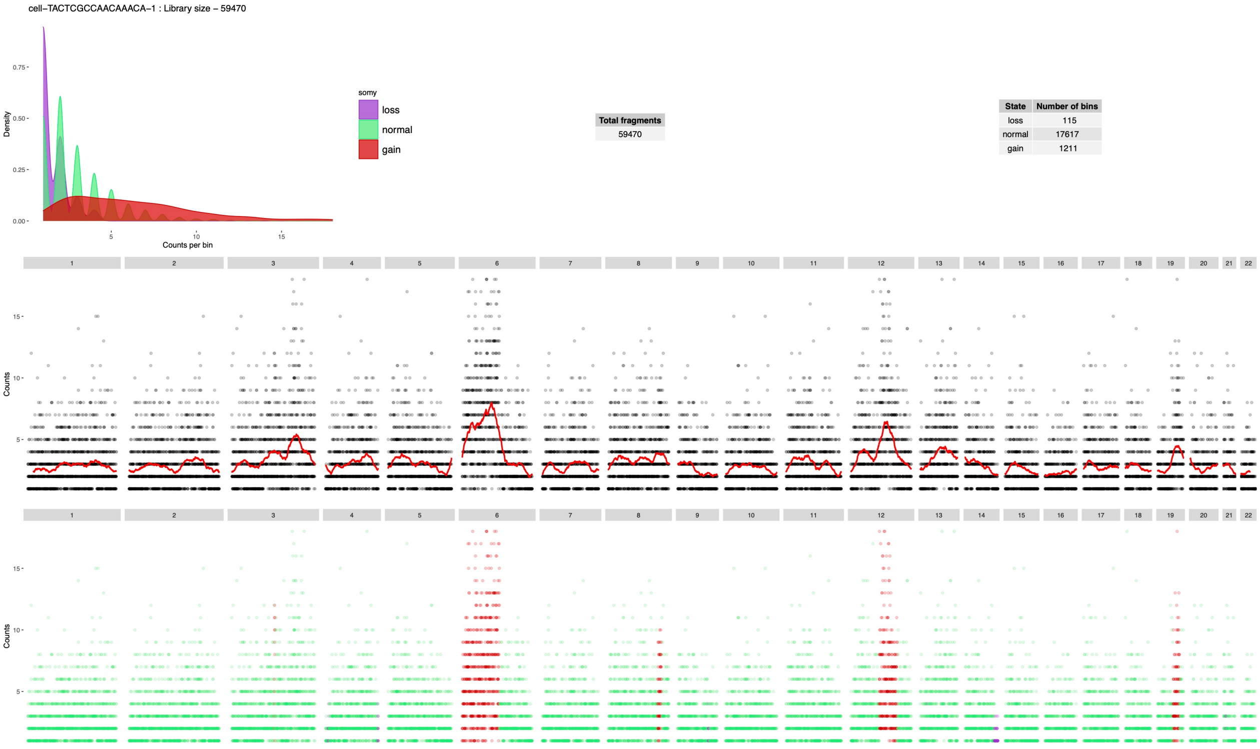

Figure S1: Number of reads distribution per somy

h)

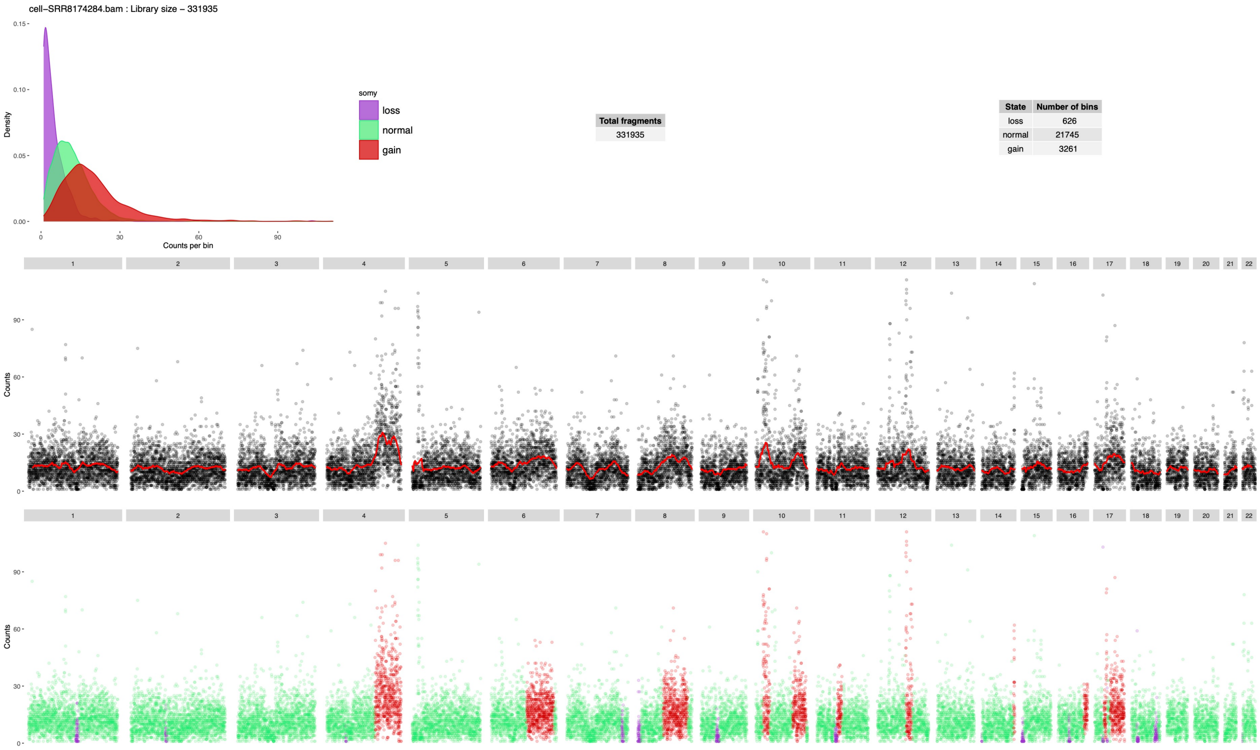

i)

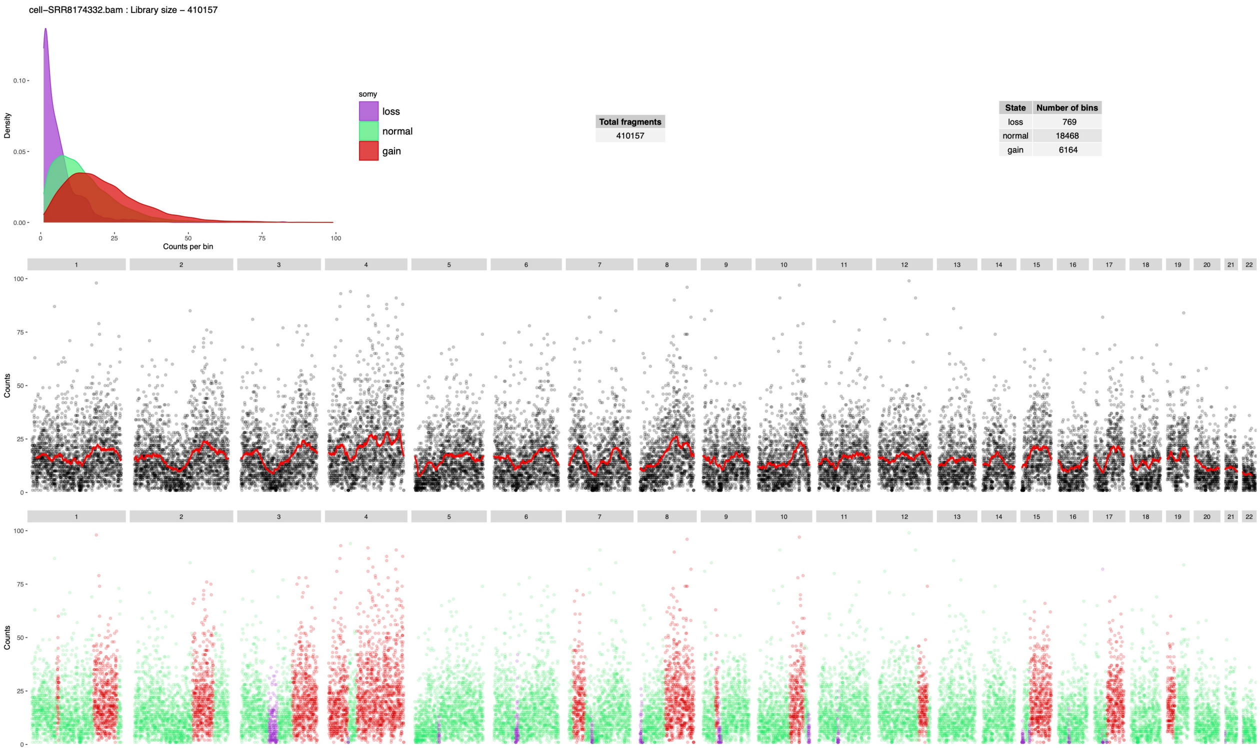

Figure S1: Number of reads distribution per somy

j)

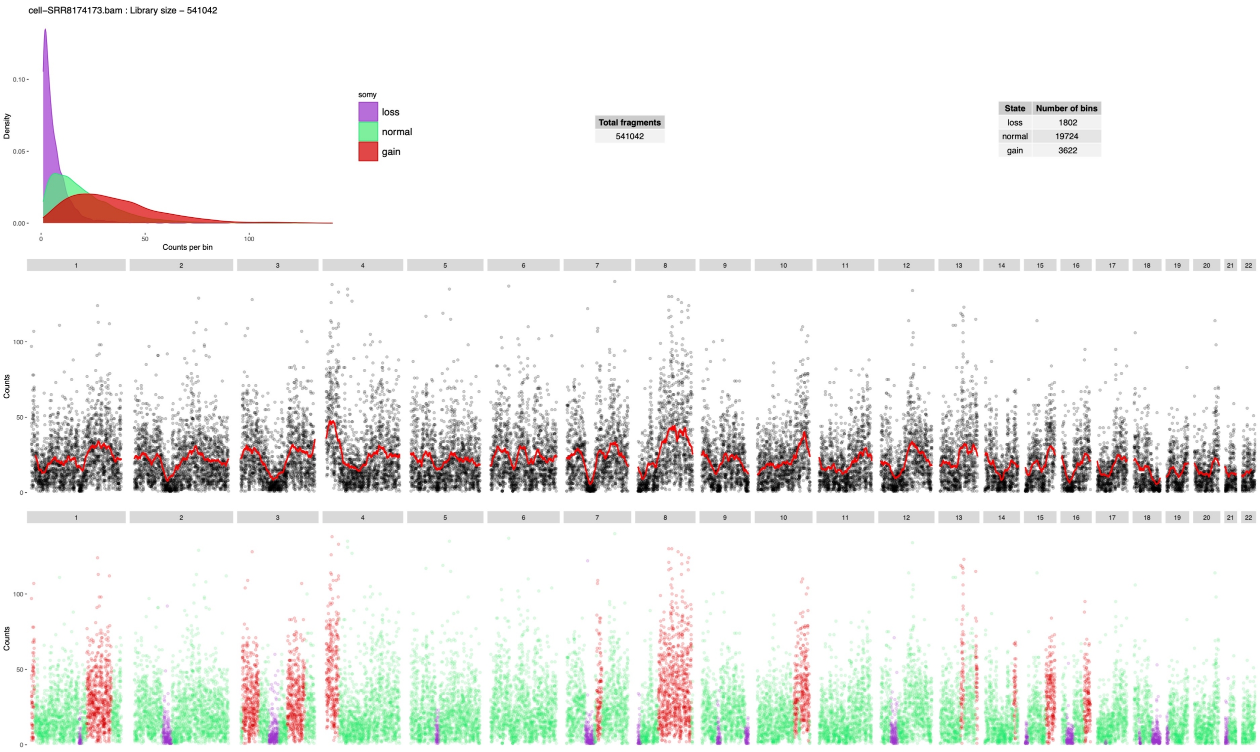

k)

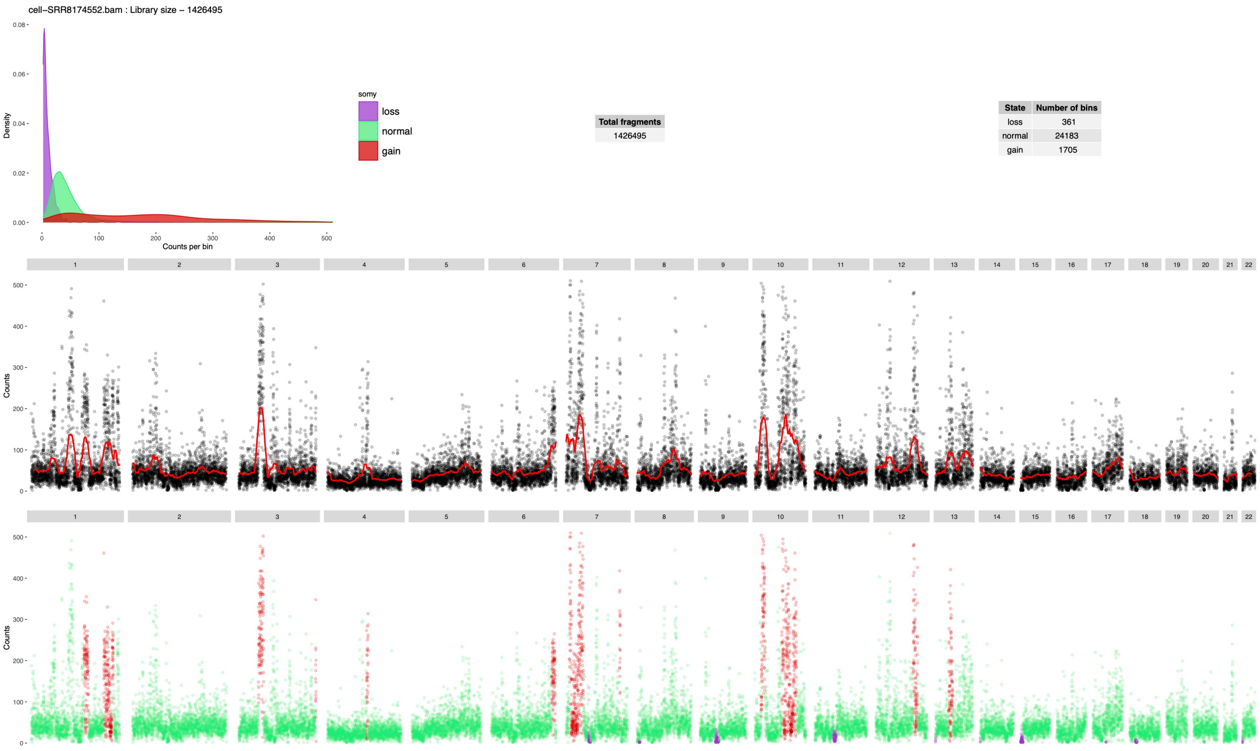

Figure S1: Number of reads distribution per somy

l)

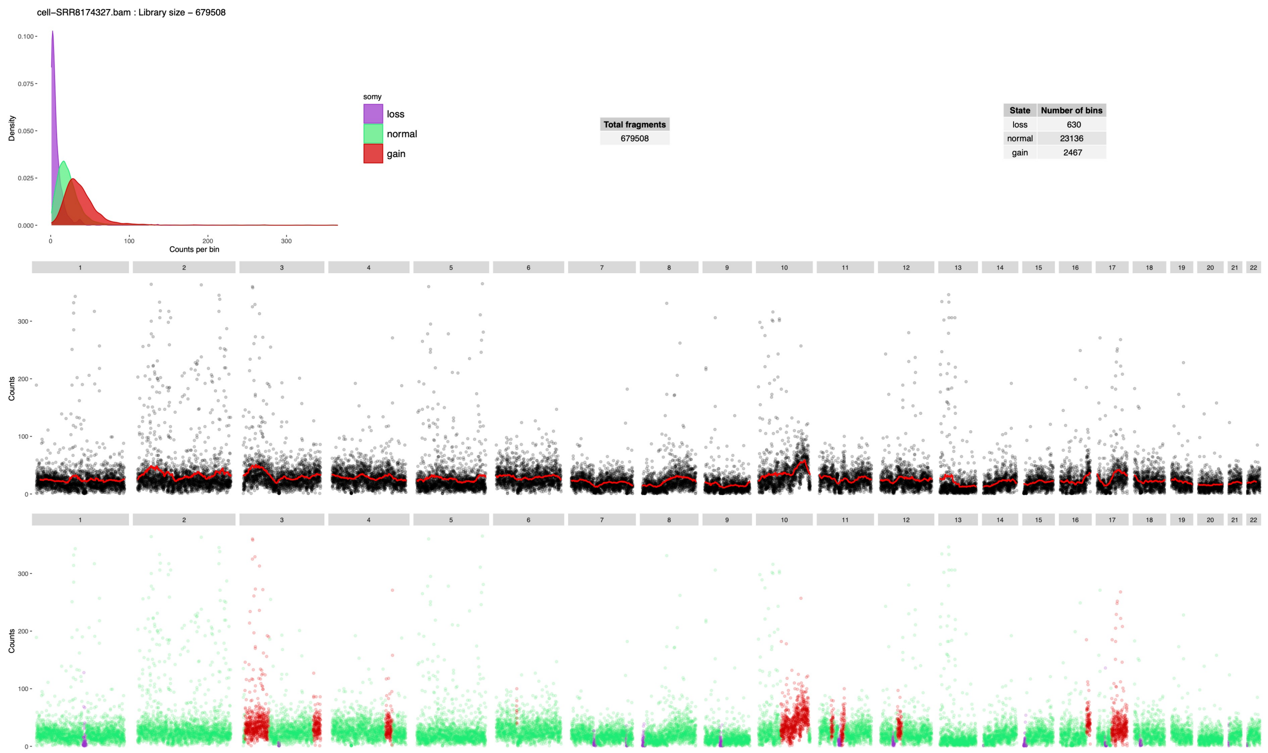

m)

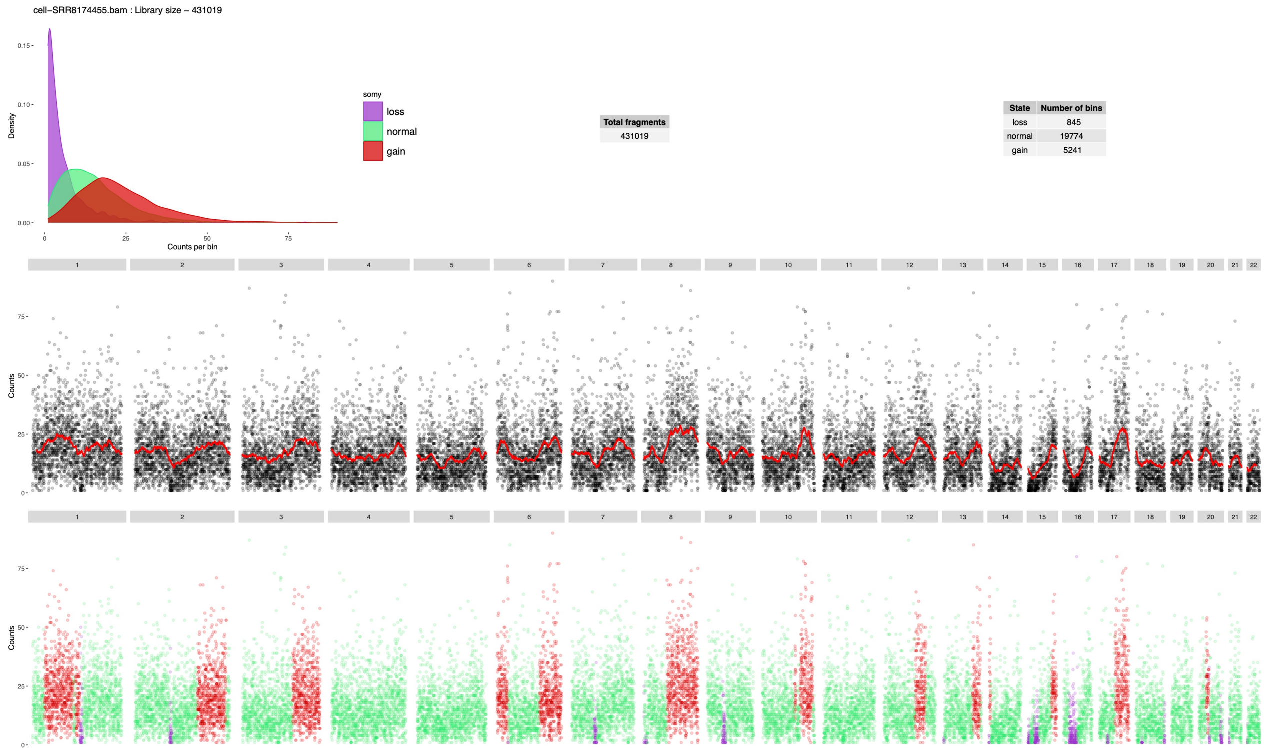

Figure S1: Number of reads distribution per somy

n)

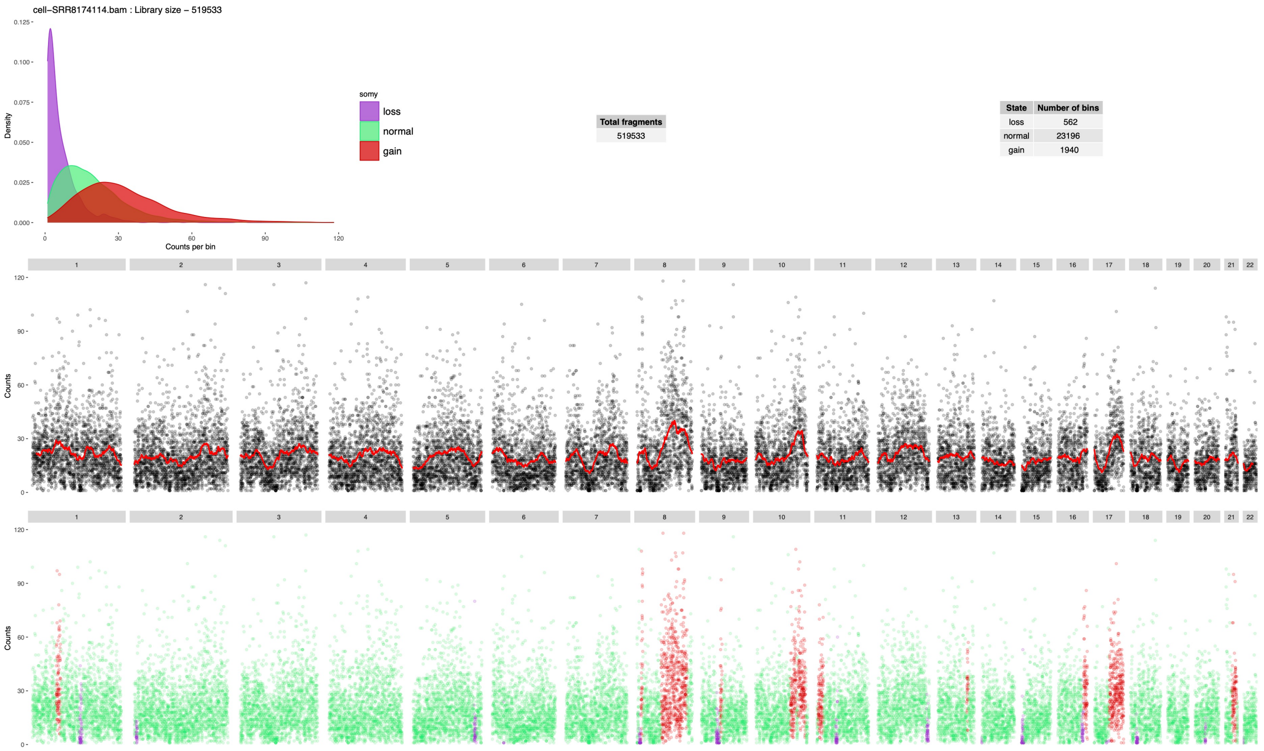

Figure S1: a-g) Different examples of the CNV calls for cells from the SNU601 cell line (Wu et al. 2021) and h-n) Different examples of the CNV calls for cells from the HCT116 cell line (Liu et al. 2019), for cells with different coverages. The histogram shows the number of reads mapping into every 100 000 bp bin, for the regions identified as lost (purple), gained (red) and normal (green). The genome-wide representations (chromosome 1 to 22) show the number of counts per 100 000 bp bin for the same cell, with copy numbers indicated by purple (loss), red (gain) and green (normal). The rolling mean (red line) was calculated on the counts per bin with a window size of 200.

Figure S2: SNU segmentation with clustering results for scATAC-seq

a)

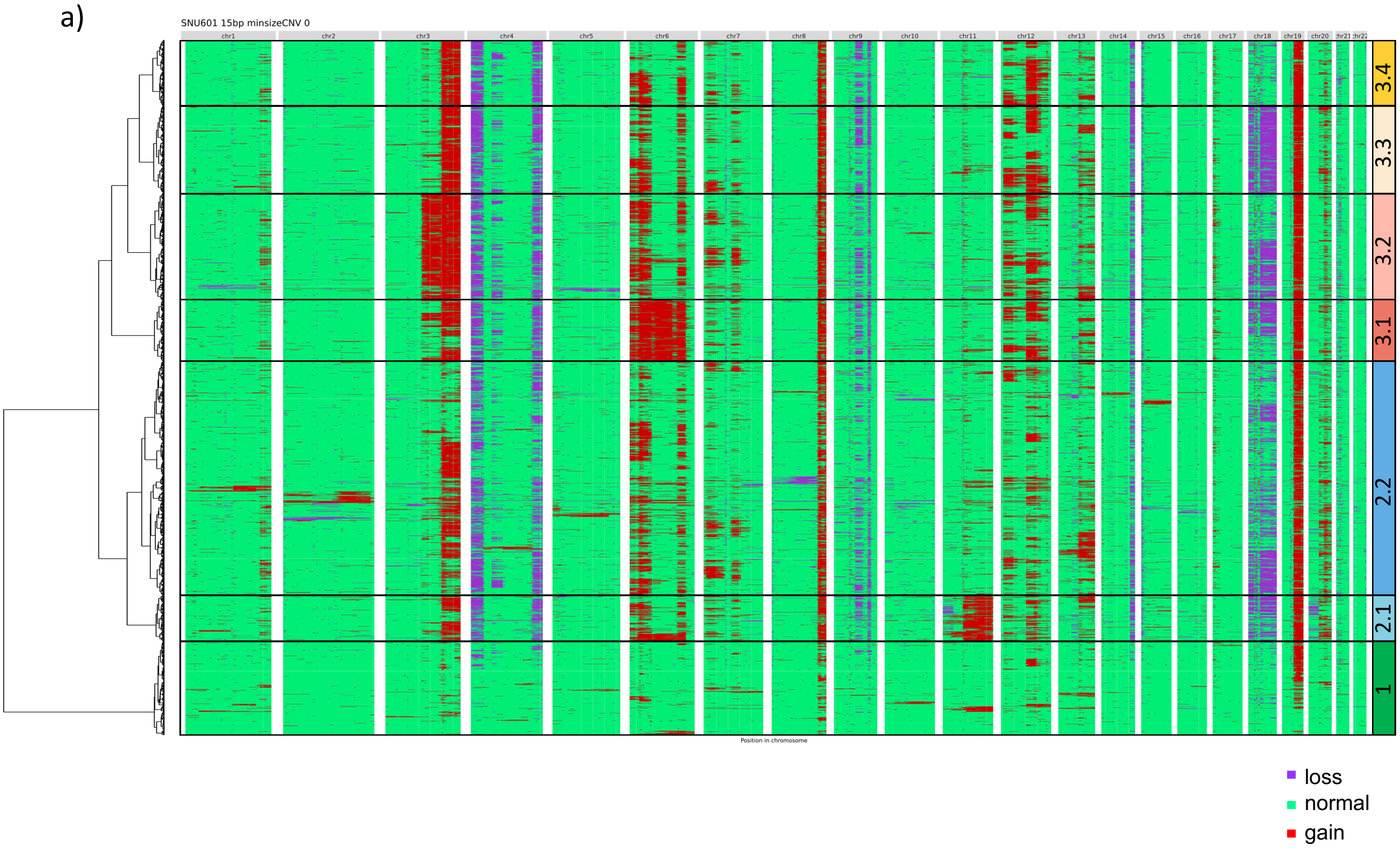

b)

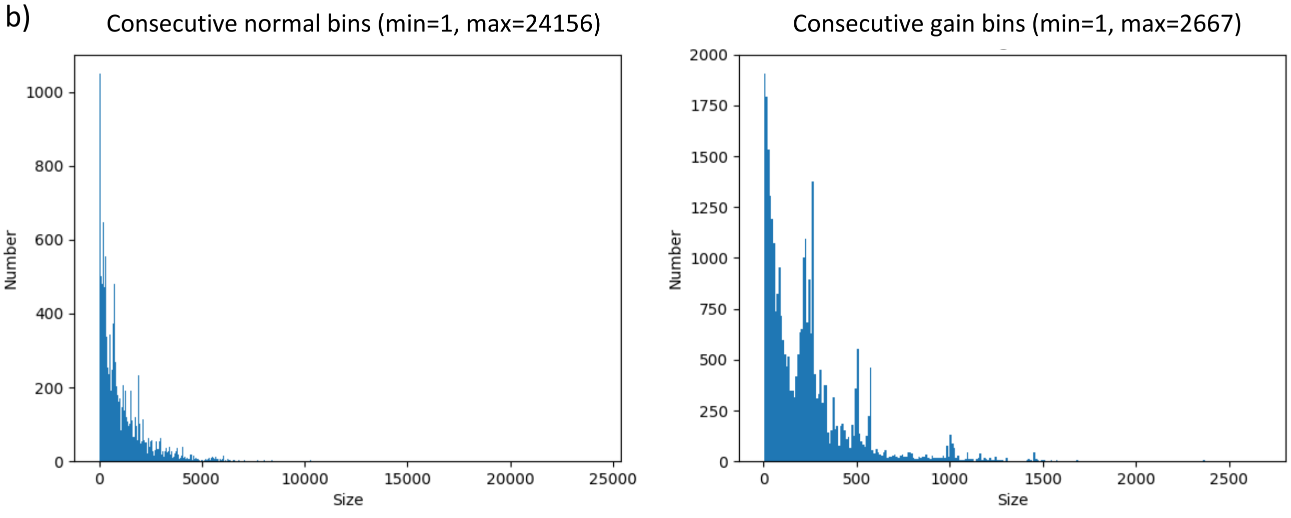

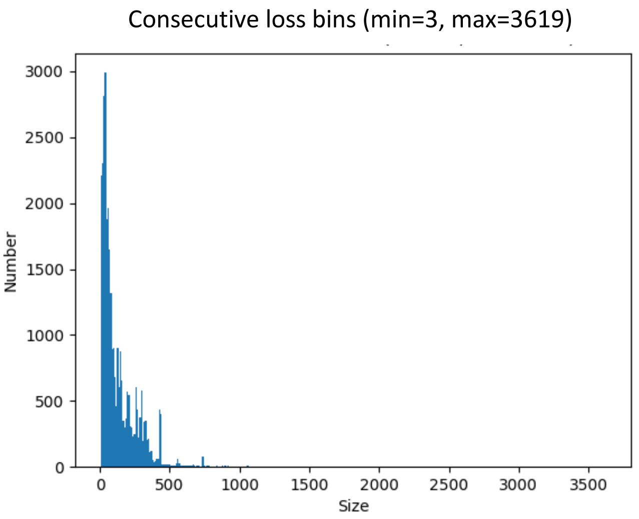

Figure S2: a) Karyogram per single-cell (every cell is a row, every column is a chromosome) for the SNU601 scATAC-seq dataset (Wu et al. 2021), with gains indicated in red, losses in pruple and normal state in green. The cells are ordered based on the clustering results (clustering based on their copy number profiles using Euclidean distance and Ward Clustering). b) Length distribution of the consecutive normal bins, gained bins and loss bins

Figure S2: SNU segmentation with clustering results for scATAC-seq

c)

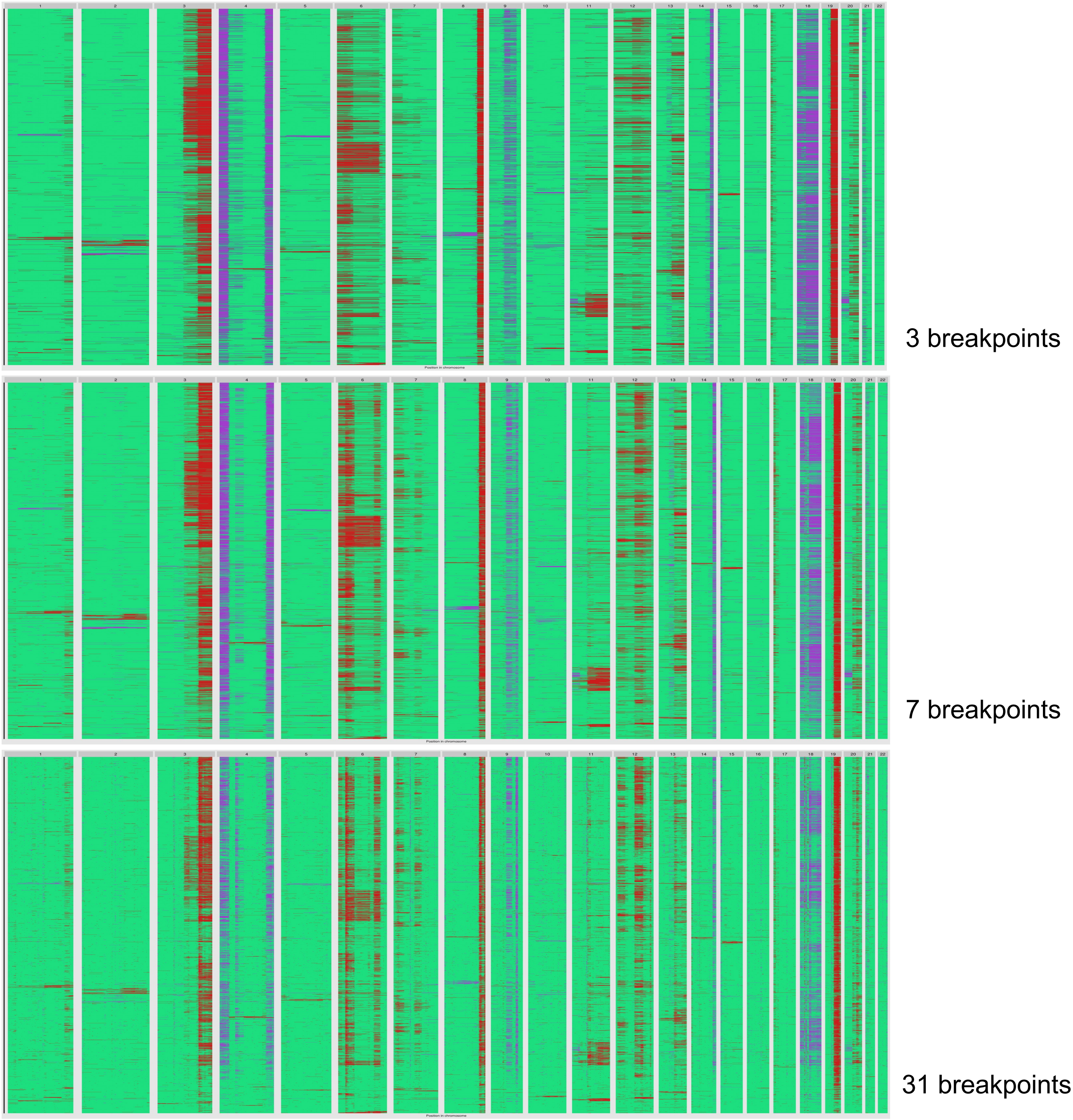

d)

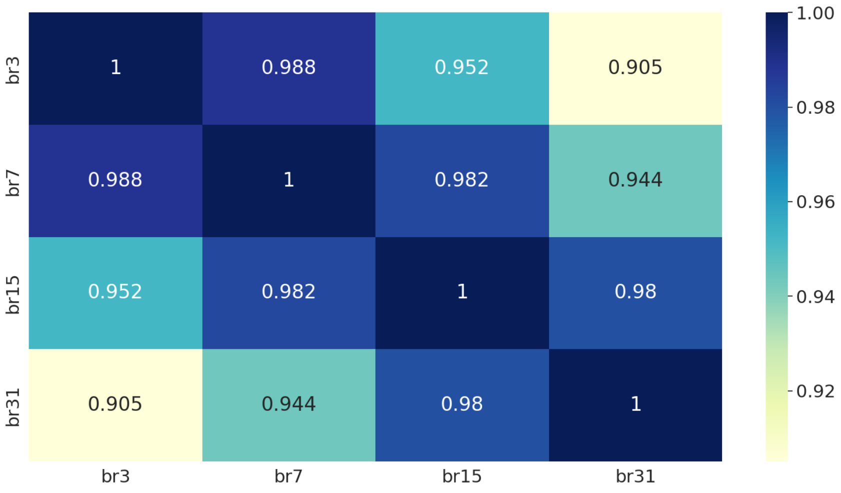

Figure S2: c) karyograms for the SNU601 scATAC-seq dataset (Wu et al. 2021) (like in panel a) calculated with 3, 7 and 31 maximum number of breakpoints per chromosome. d) correlation of pseudo bulk CNV profiles for the following number of breakpoints: 3, 7, 15 and 31.

Figure S3: SNU601 segmentation for WGS

a)

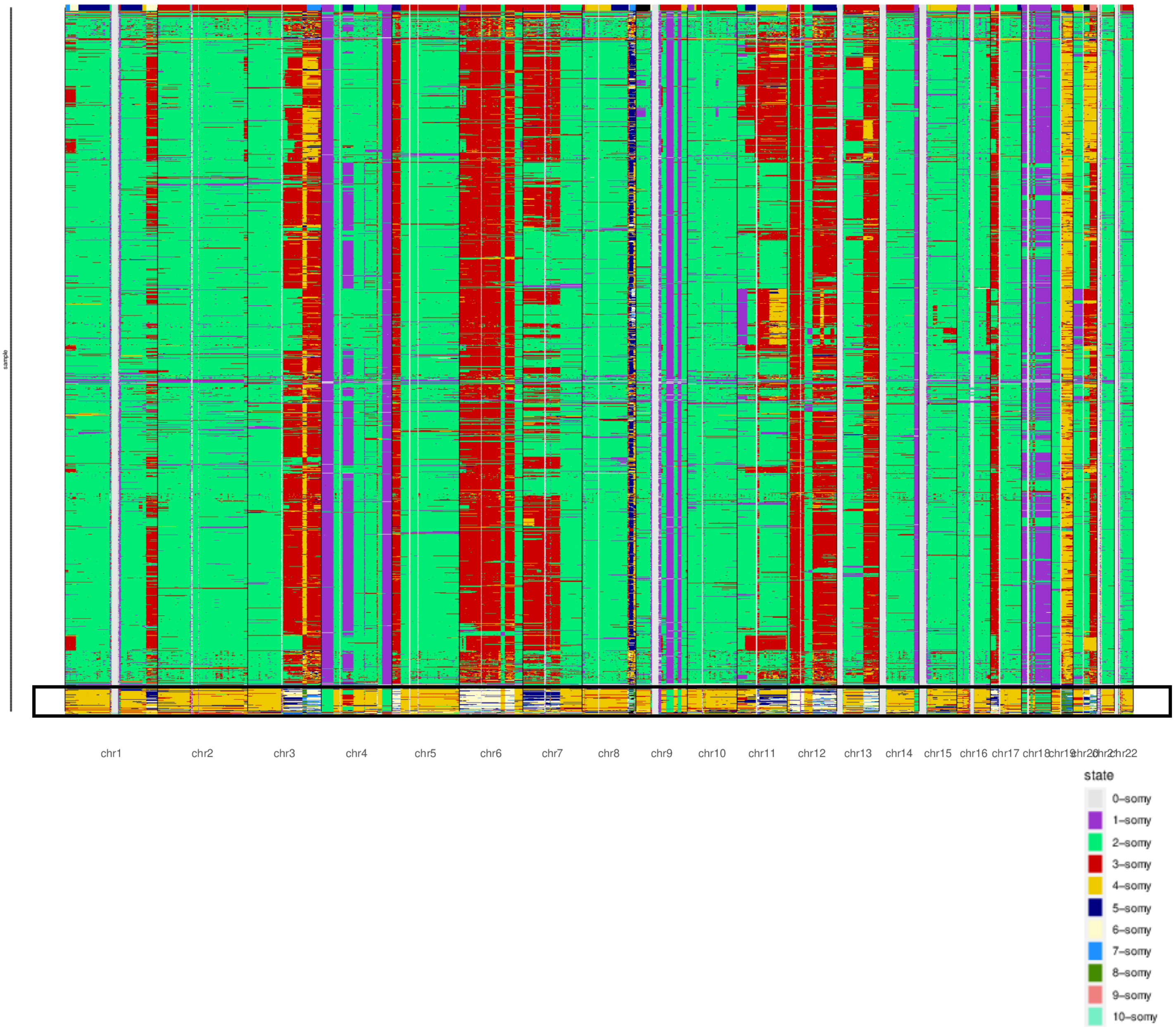

Figure S3: a) Karyogram per single-cell (every cell is a row, every column is a chromosome) for the SNU601 scWGS dataset (Andor et al. 2020). The copy numbers are marked with a color code (see legend). The cells marked with a rectangle corresponded to a separate cluster with high ploidy and were removed.

Figure S3: SNU601 segmentation for WGS

b)

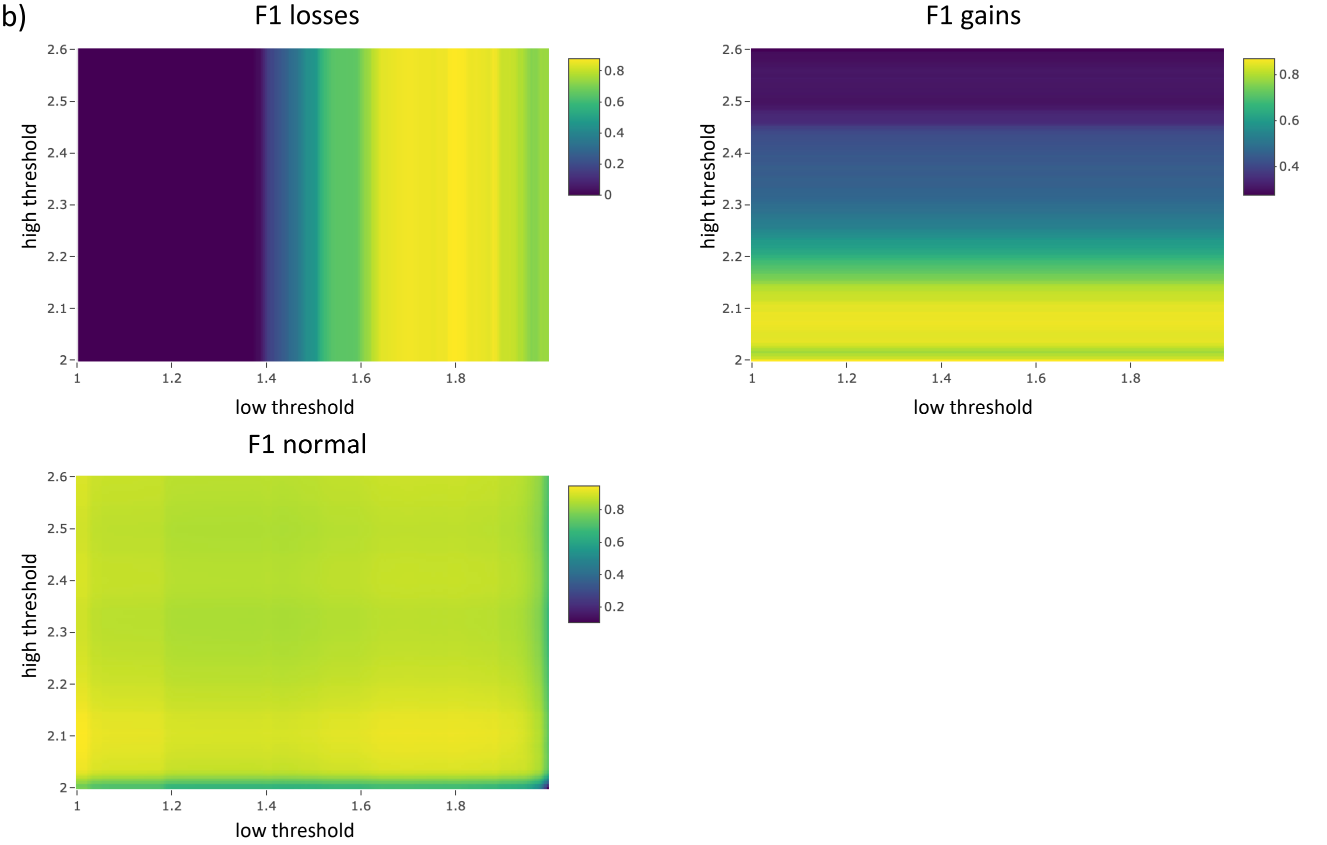

c)

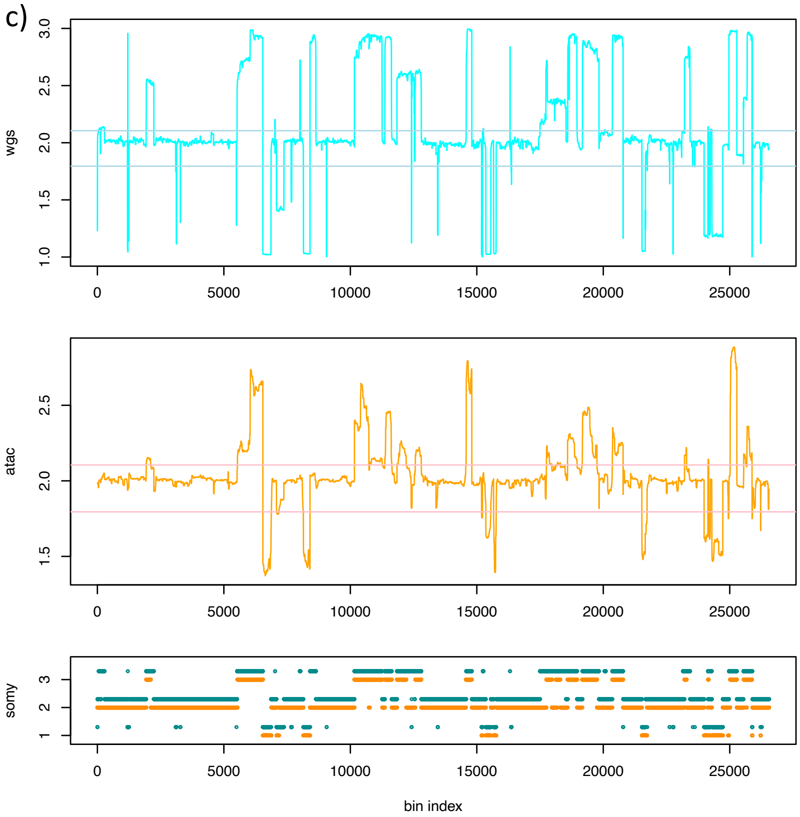

	precision	recall	F1
loss	0.939979123173278	0.82350251486054	0.877894223738728
normal	0.871109930961232	0.990818483884174	0.927116009608591
gain	0.992428153501979	0.737939859245042	0.846469983854396

Figure S3: b) F1 scores for the normal, loss and gain states in the comparison between the pseudobulk results of the scATAC-seq and the scDNA karyotypes for the SNU601 cell line. The low and high thresholds refer to the threshold used for calling losses and the threshold used for calling gains, respectively. c) example of the low and high threshold that lead to the best F1 values for all three states. Top: WGS; middle: pseudo bulk scATAC; bottom: gain (3), normal (2) or loss (1) per bin for the scATAC pseudo bulk (orange) and the WGS (green).

Figure S4: HCT116 segmentation for scATAC-seq and scDNA-seq

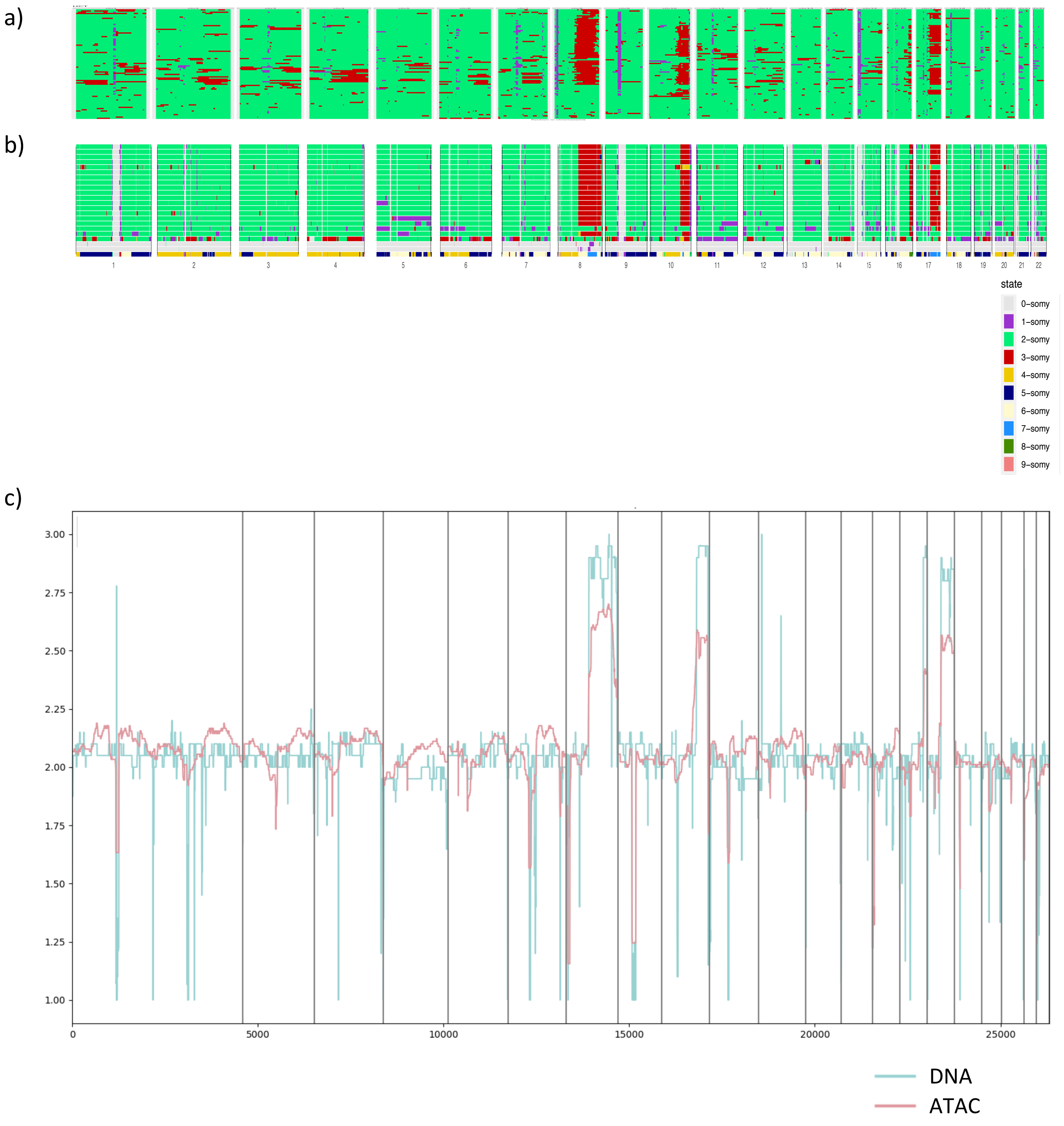

Figure S4: Karyogram plots for the HCT116 cell line for the a) scATAC-seq dataset (Liu et al 2019) and the b) scDNA-seq dataset (Cohen-Sharir et al. 2021). c) comparison of the pseudobulk results between the scATAC-seq and the scDNA-seq results. Note that the pseudo bulk for the sc genome sequencing is calculated from only 22 cells, hence the jagged single cell genome sequencing profile, especially seen in the small sharp losses which are mostly due to noise in the coverage.

Figure S4: HCT116 segmentation for scATAC-seq and scDNA-seq

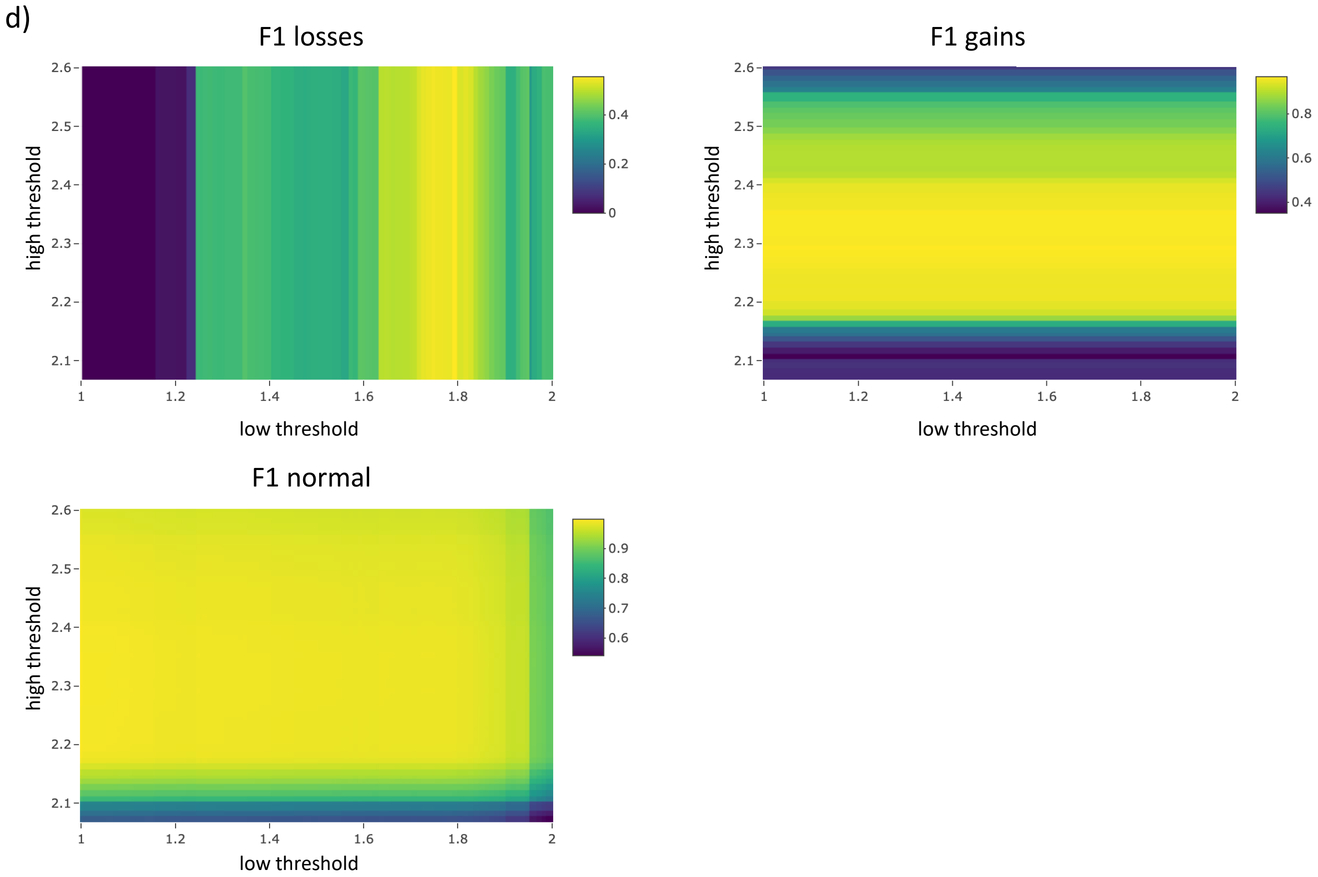

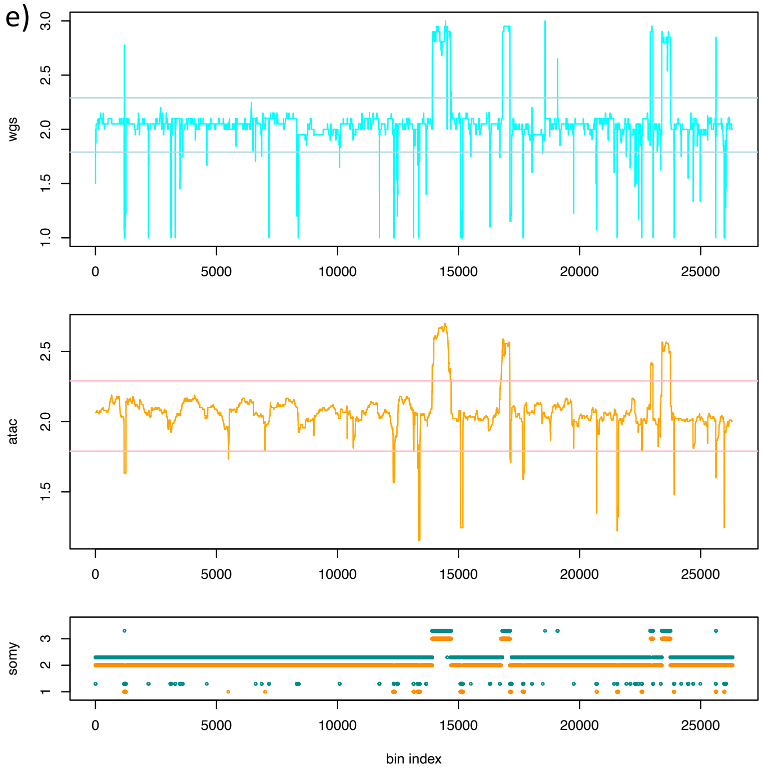

	precision	recall	F1
loss	0.623946037099494	0.501355013550135	0.555972952667167
normal	0.983336096833029	0.988169624260602	0.985746935383337
gain	0.95875	0.975206611570248	0.966908288685786

Figure S4: d) F1 scores for the normal, loss and gain states in the comparison between the pseudobulk results of the scATAC-seq and the scDNA karyotypes for the HCT cell line. The low and high thresholds refer to the threshold used for calling losses and the threshold used for calling gains, respectively. e) example of the low and high threshold that lead to the best F1 values for all three states. Top: WGS; middle: pseudo bulk scATAC; bottom: gain (3), normal (2) or loss (1) per bin for the scATAC pseudo bulk (orange) and the WGS (green).

Figure S5: colo320 segmentation for scATAC-seq and WGS data

a)

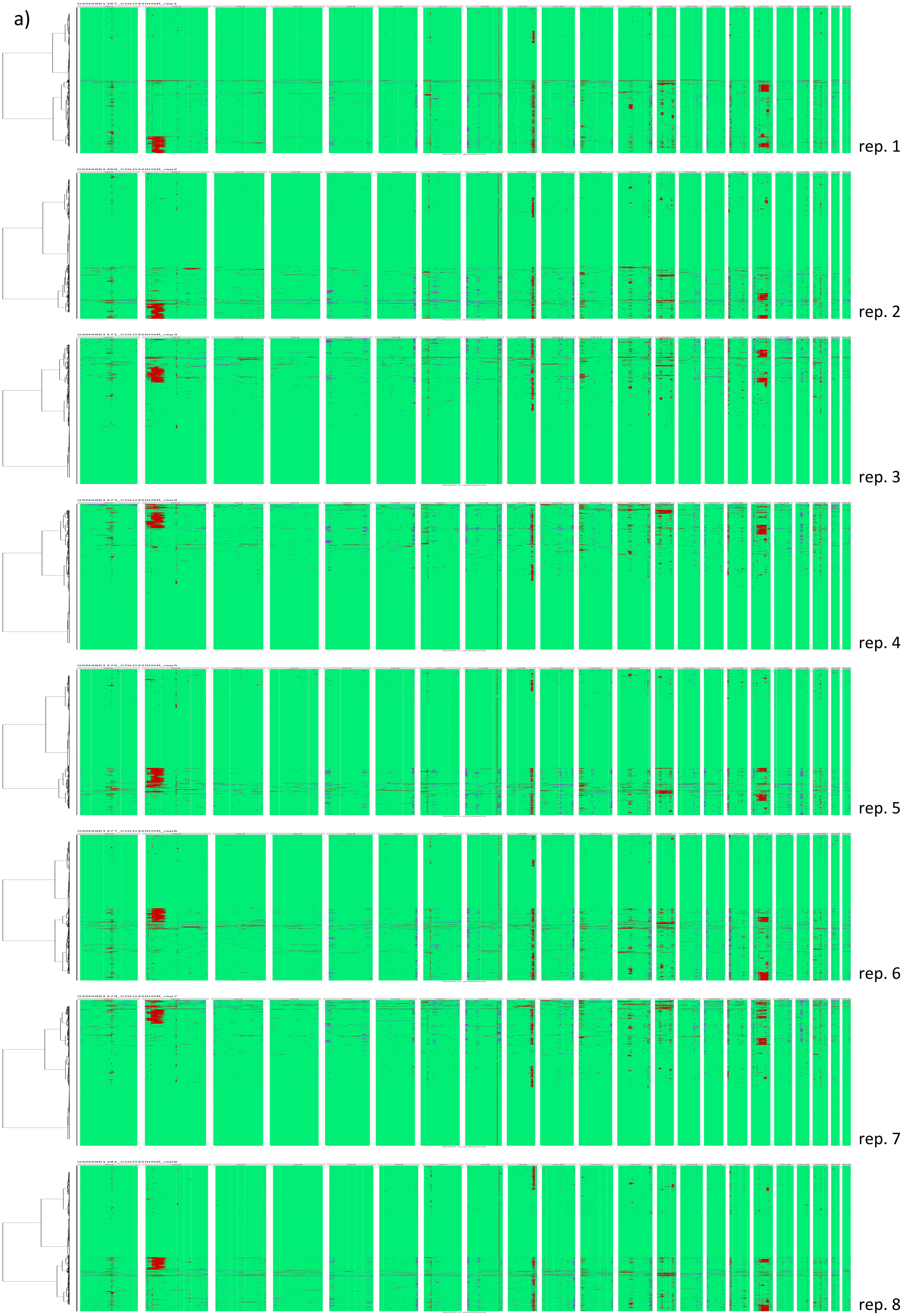

Figure S5: colo320 segmentation for scATAC-seq and WGS data

b)

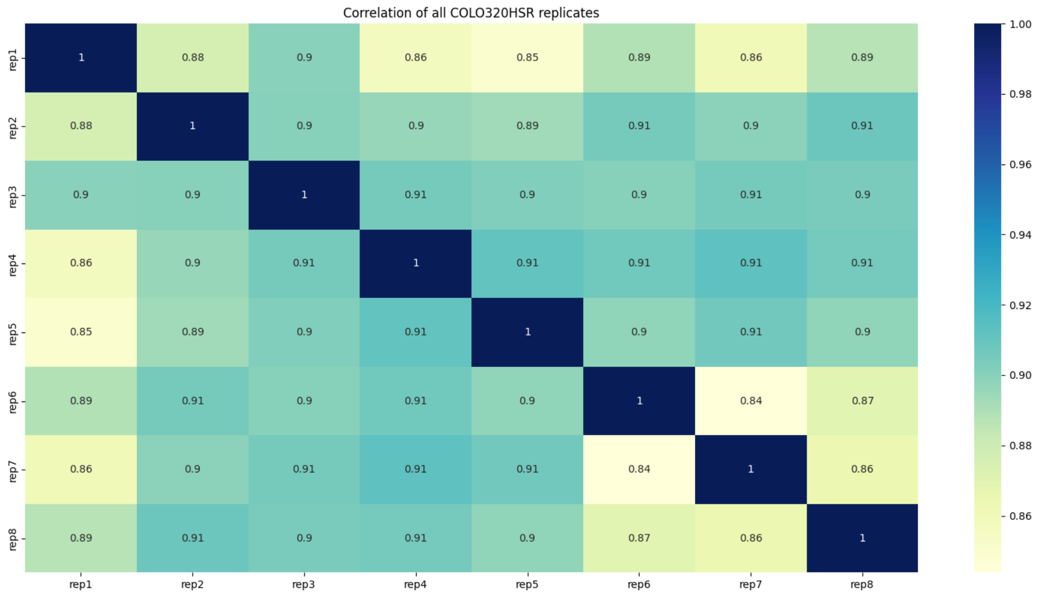

c)

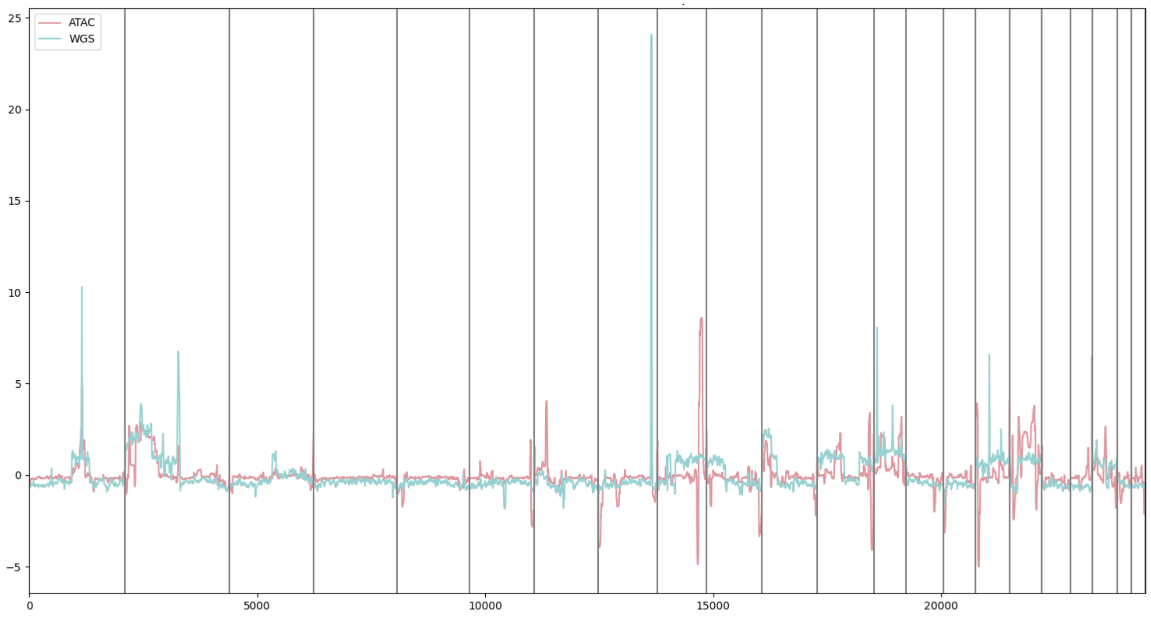

Figure S5: a) Karyogram plots for the eight colo320 replicate (Hung et al 2021). b) correlation between the karyogram results of the eight colo320 replicates (genome-wide correlation between pseudobulk karyotype profiles). c) genome-wide pseudobulk karyotype profile for the scATAC-seq (replicate 1) compared to the whole genome sequencing signal for the colo320 cell line (Hoffman et al. 2019).

Figure S5: colo320 segmentation for scATAC-seq and WGS data

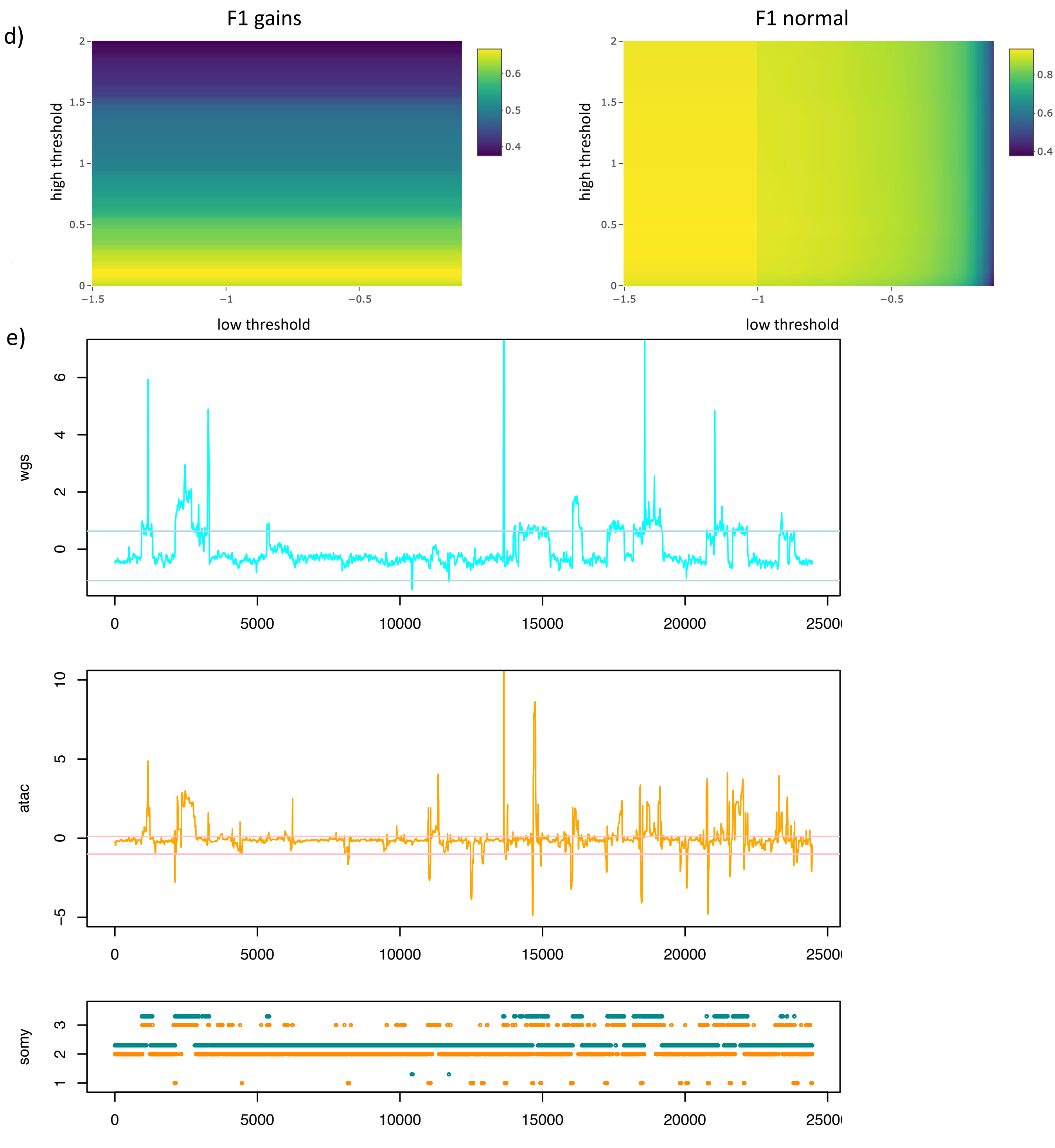

	precision	recall	F1
loss	NA	NA	NA
normal	0.933056278790306	0.922205176424011	0.927598994499614
gain	0.649810648251281	0.685707569346497	0.667276678485646

Figure S5: d) F1 scores for the normal and gain states in the comparison between the pseudobulk results of the scATAC-seq (replicate 1) and the WGS signal. The low and high thresholds refer to the threshold used for calling losses and the threshold used for calling gains in the scATAC-seq data, respectively. The precision and recall were calculated with varying scATAC-seq limits while keeping the WGS limits fixed. There were only residual losses identified in this dataset and therefore the F1 values for losses was not calculated. e) example of the low and high threshold that lead to the best F1 values. Top: WGS; middle: pseudo bulk scATAC; bottom: gain (3), normal (2) or loss (1) per bin for the scATAC pseudo bulk (orange) and the WGS (green).

Figure S6: Primary glioblastoma sample segmentation for scATAC-seq and WGS data

a)

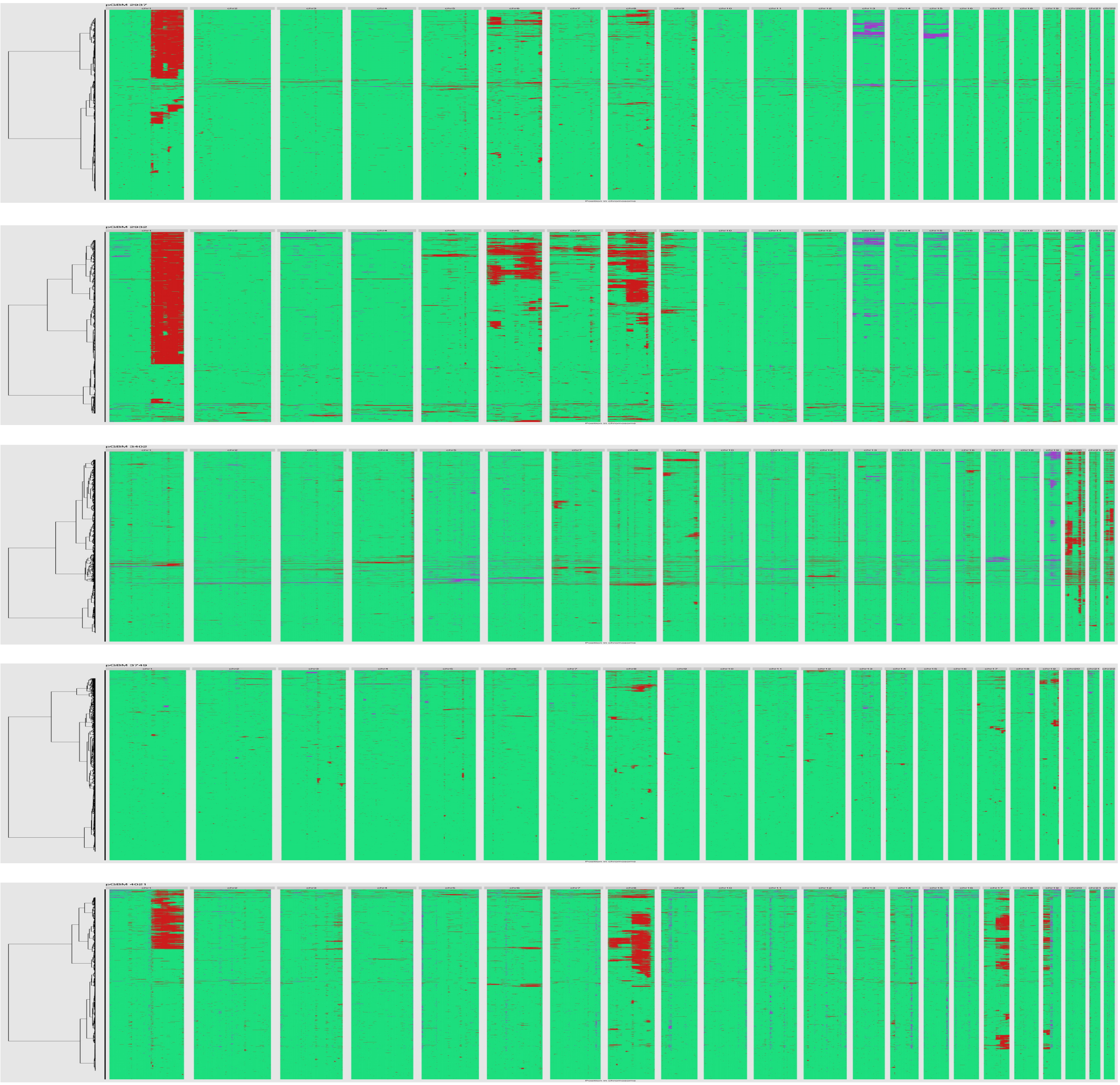

2937

2932

3402

3749

4021

Figure S6: a) karyograms for the pediatric glioblastoma samples, for five different biological samples (2932 and 3749 (diagnostic); 2937, 3402 and 4021 (relapse)) (Wu et al. 2019).

Figure S6: Primary glioblastoma sample segmentation for scATAC-seq and WGS data

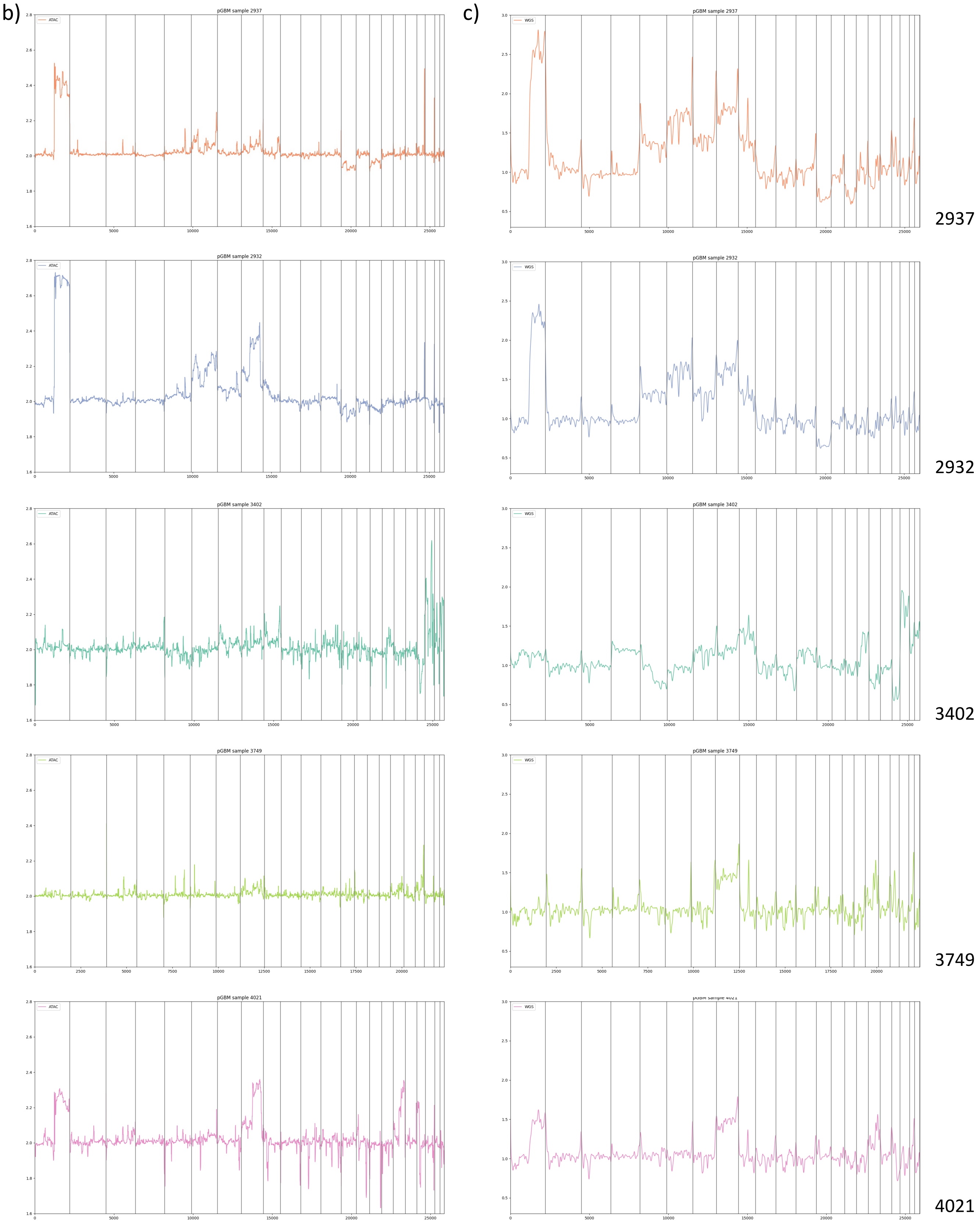

Figure S6: b) pseudo bulk aggregates for the pediatric glioblastoma samples, for five different samples (2937, 2932, 3402, 3749 and 4021) (Wu et al. 2019). c) WGS signal for the same samples. From the comparison between the scATAC aggregates and the WGS signal, it can be noticed that samples 3402 and 3749 are in both cases nearly disomic.

Figure S6: Primary glioblastoma sample segmentation for scATAC-seq and WGS data

d)

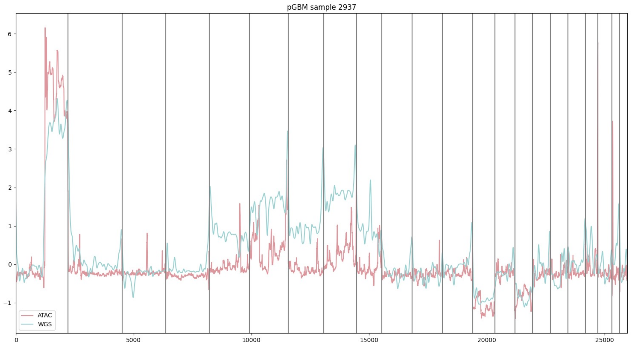

2937

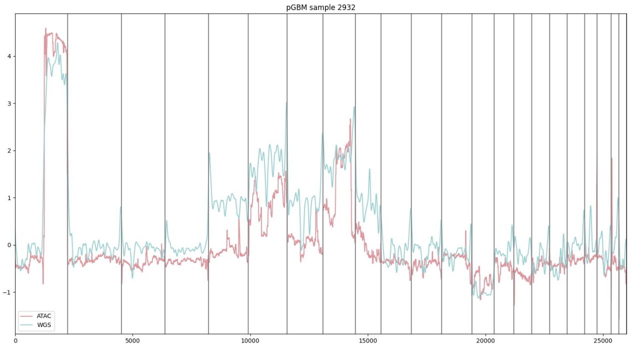

2932

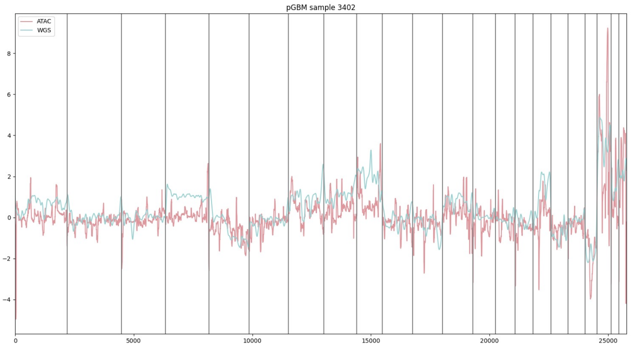

3402

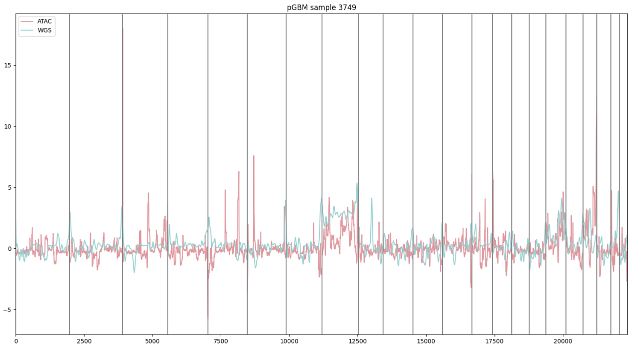

3749

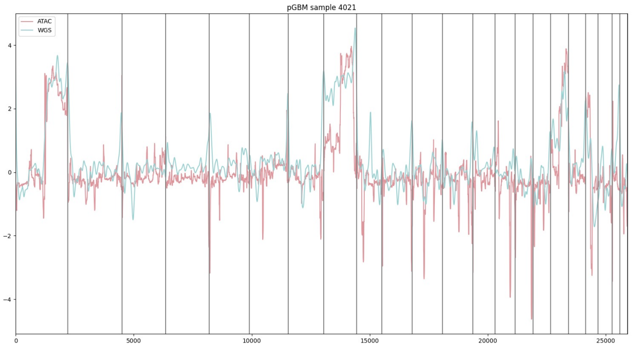

4021

Figure S6: d) same profiles as before, where both signals have been standardized (mean=1 and std=0) in order for the two signals to be placed in the same scale (resulting in a different scale per sample). Samples 3402 and 3749 are nearly disomic in both modalities.

Figure S6: Primary glioblastoma sample segmentation for scATAC-seq and WGS data

e)

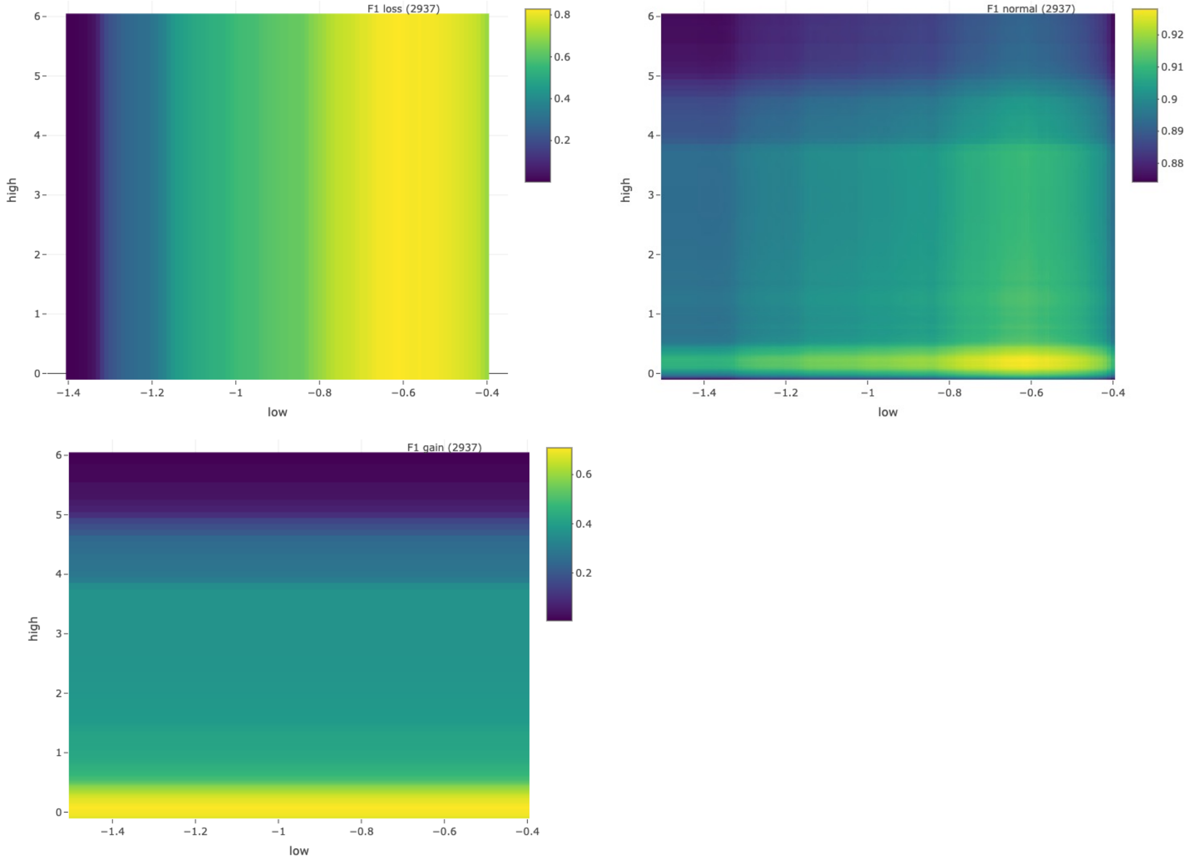

f)

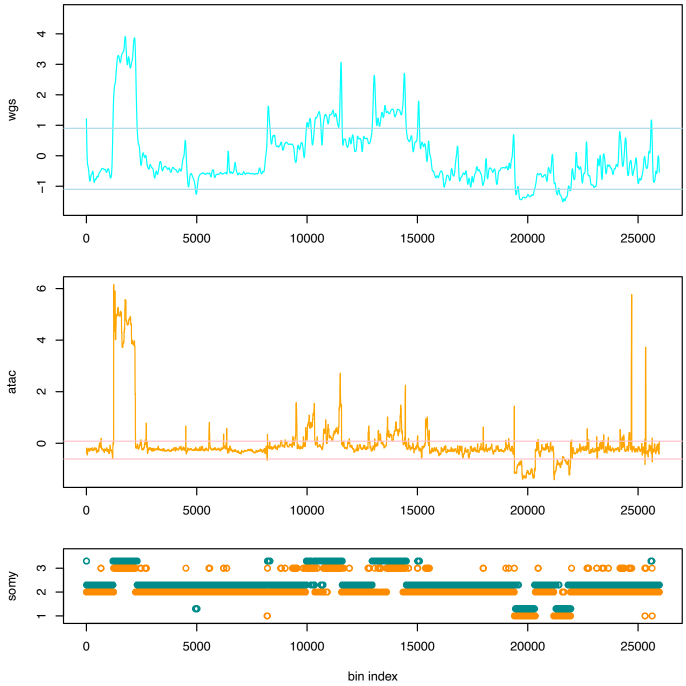

	precision	recall	F1
loss	0.774386594853381	0.889347079037801	0.827895073576456
normal	0.927005851433105	0.925033400959968	0.926018575851393
gain	0.723703344643723	0.694580134915097	0.708842729970326

Figure S6: Primary glioblastoma sample segmentation for scATAC-seq and WGS data

g)

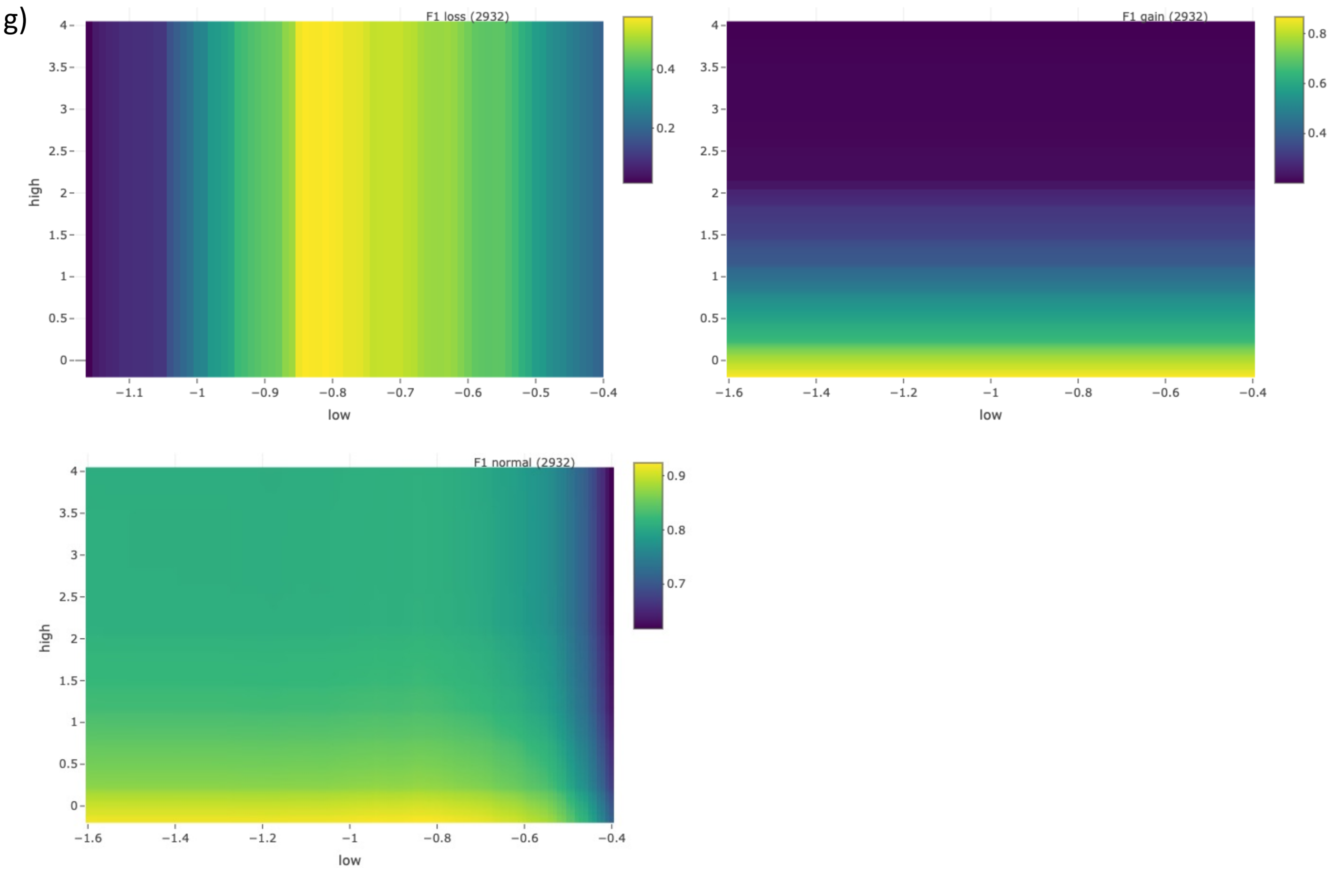

h)

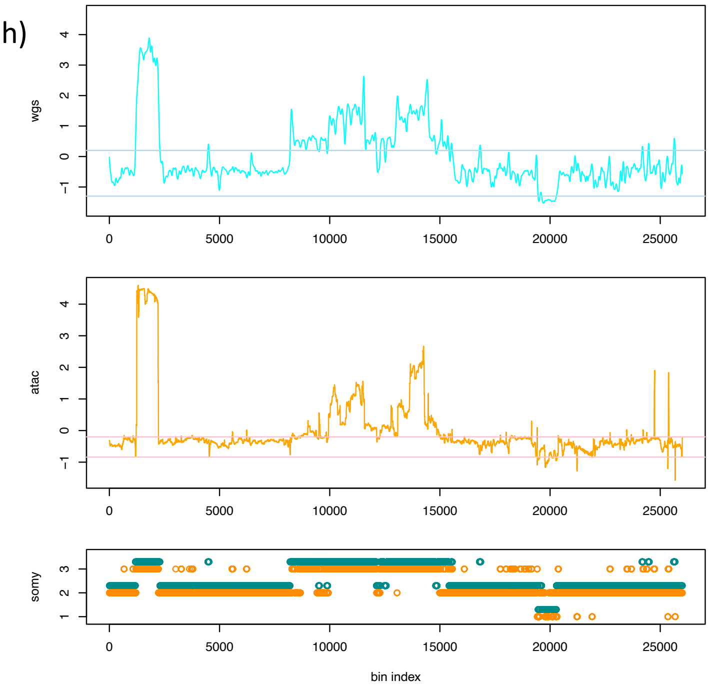

	precision	recall	F1
loss	0.735112936344969	0.476063829787234	0.577885391444713
normal	0.902585722315908	0.945419218087612	0.923506067751769
gain	0.896685655100984	0.837991530550514	0.866345612608668

Figure S6: Primary glioblastoma sample segmentation for scATAC-seq and WGS data

i)

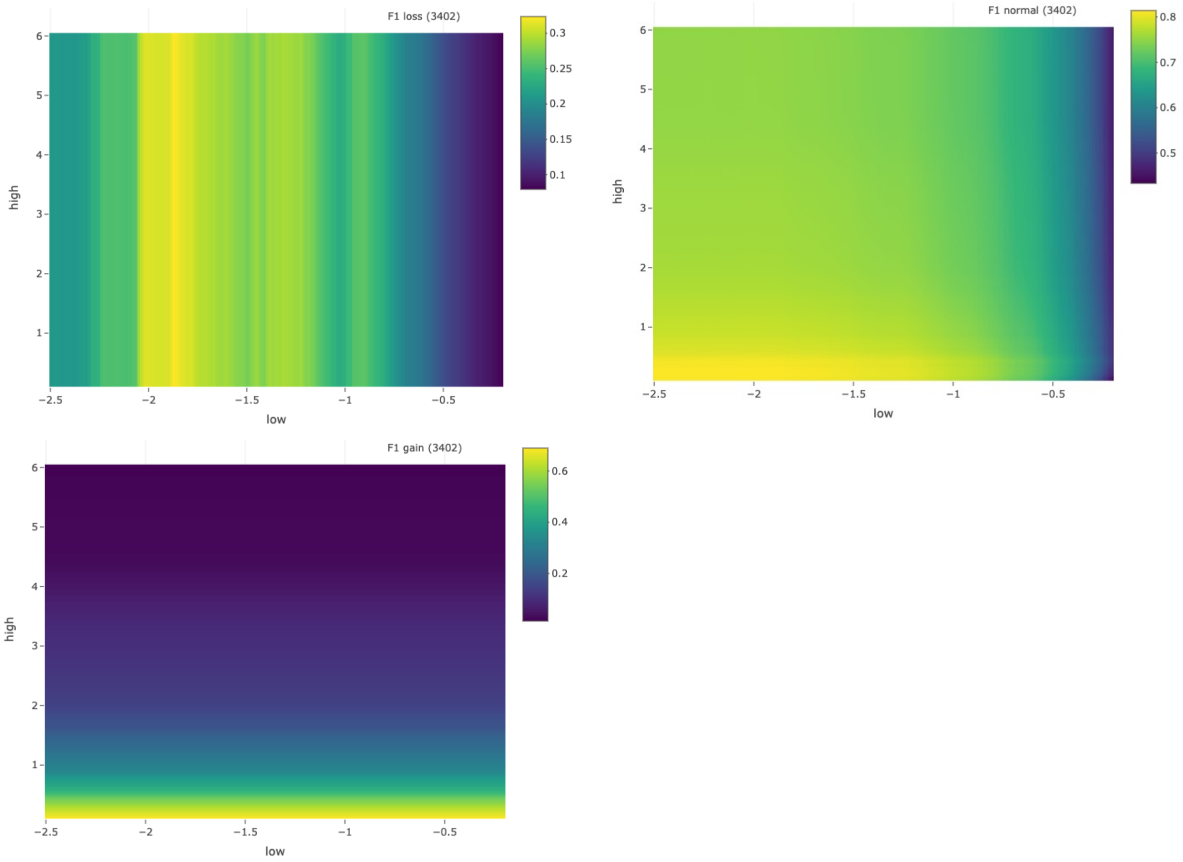

j)

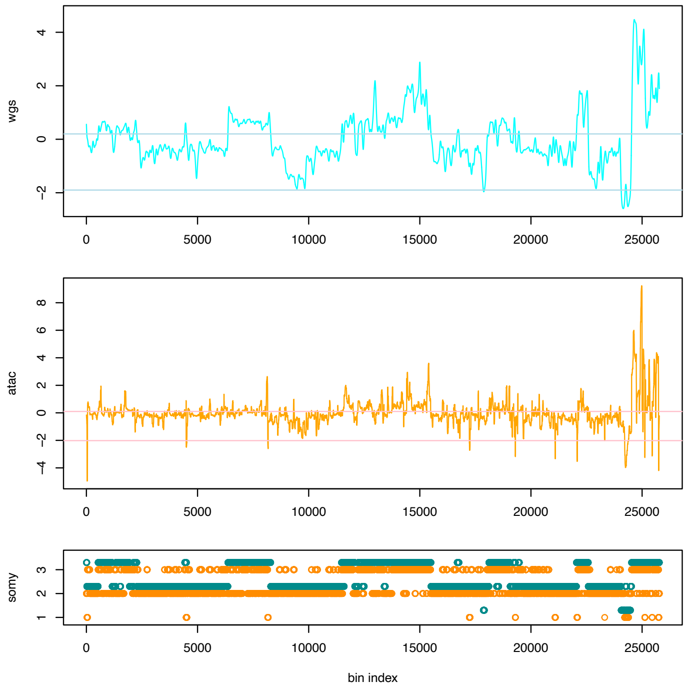

*

	precision	recall	F1
loss	0.339080459770115	0.286407766990291	0.310526315789474
normal	0.77697415224093	0.848724258515736	0.811265861962241
gain	0.744479495268139	0.642273964318113	0.68961038961039

* This sample is nearly disomic in both modalities (see a, b, c). Therefore, the comparison of gains and losses between datasets is very much noise driven, resulting in lower F1 scores for these two states.

Figure S6: Primary glioblastoma sample segmentation for scATAC-seq and WGS data

k)

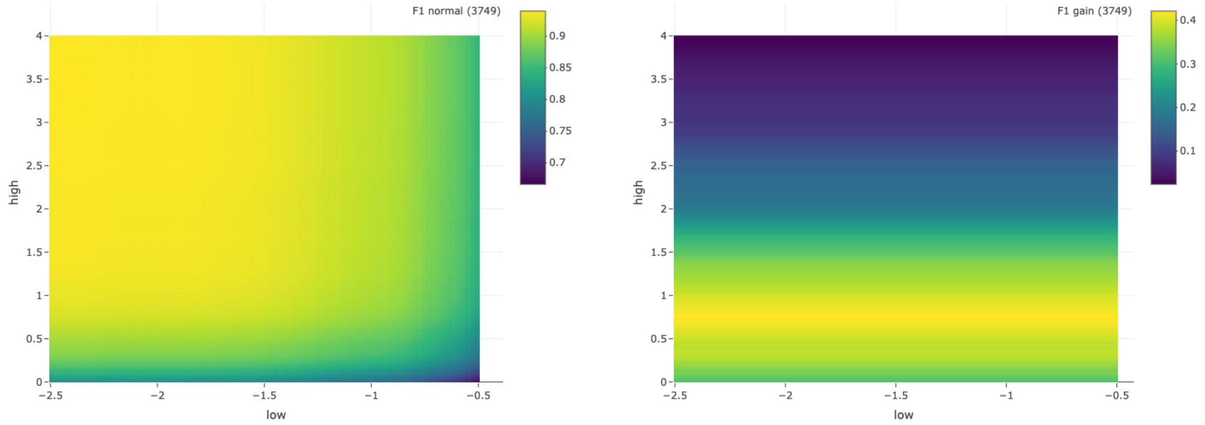

l)

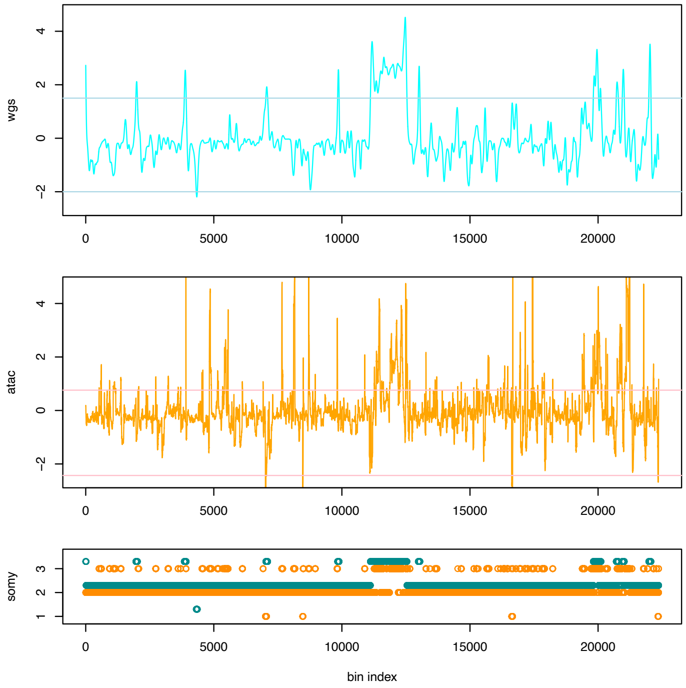

*

	precision	recall	F1
loss	0	0	NaN
normal	0.936599866741838	0.911103355437004	0.92367569753336
gain	0.383720930232558	0.465813850904279	0.42080095636581

* This sample is nearly disomic in both modalities (see a, b, c). Therefore, the comparison of gains and losses between datasets is very much noise driven, resulting in lower F1 scores for these two states.

Figure S6: Primary glioblastoma sample segmentation for scATAC-seq and WGS data

m)

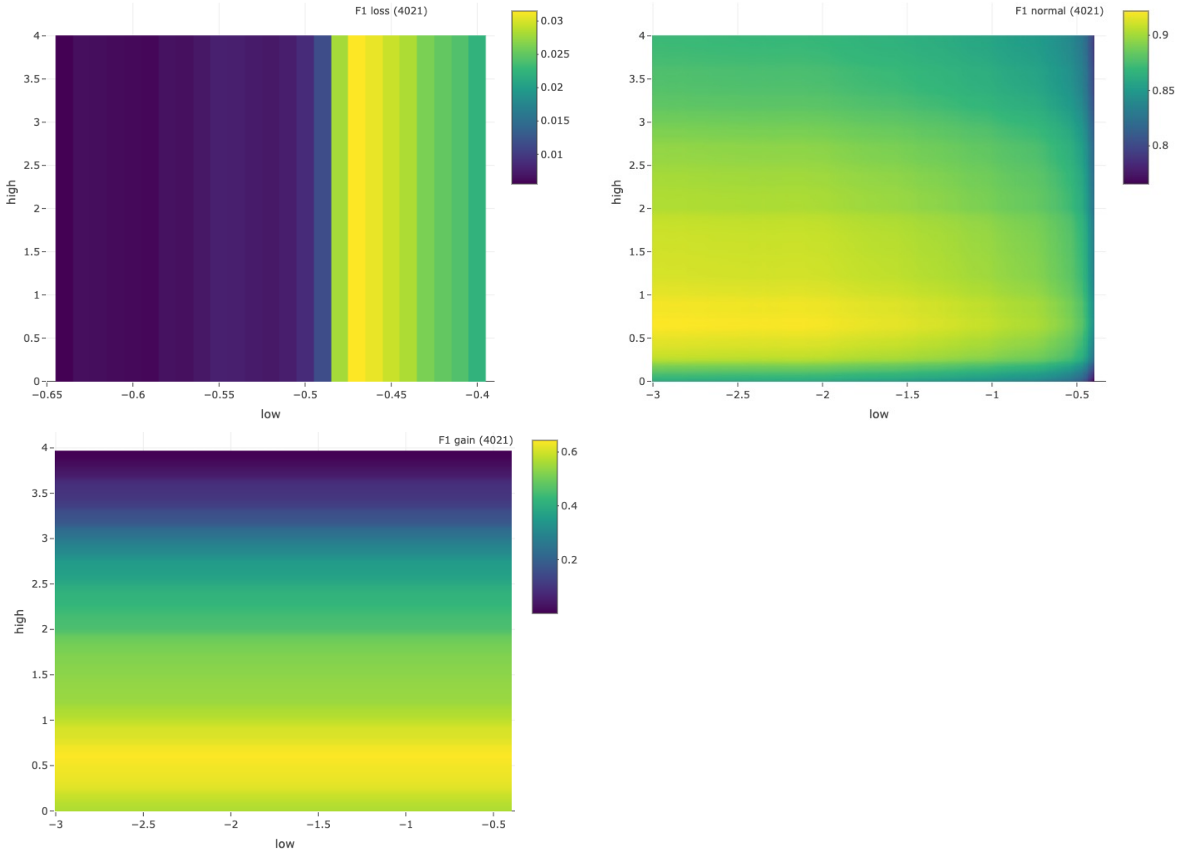

n)

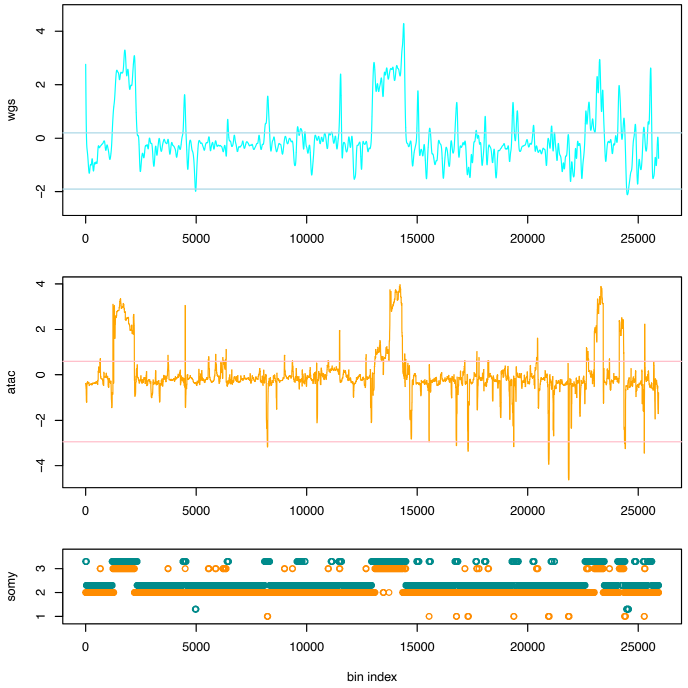

	precision	recall	F1
loss	0	0	NaN
normal	0.871859855053132	0.975814879323215	0.92091297609543
gain	0.887280566676933	0.504641793659135	0.643367574810183

Figure S6: e,g,i,k,m) F1 scores for the normal, gain and loss states in the comparison between the pseudobulk results of the scATAC-seq and the WGS signal. The low and high thresholds refer to the threshold used for calling losses and for calling gains in the scATAC-seq data, respectively. The precision and recall were calculated with varying scATAC-seq limits while keeping the WGS limits fixed. In general, no losses were present in the datasets and therefore the F1 score for losses was low (noise versus noise). For sample 3749, there were no losses identified. d,f,h,j,l) example of the low and high threshold that lead to the best F1 values. Top: WGS; middle: pseudo bulk scATAC; bottom: gain (3), normal (2) or loss (1) per bin for the scATAC pseudo bulk (orange) and the WGS (green).

Figure S7: euploid samples

a)

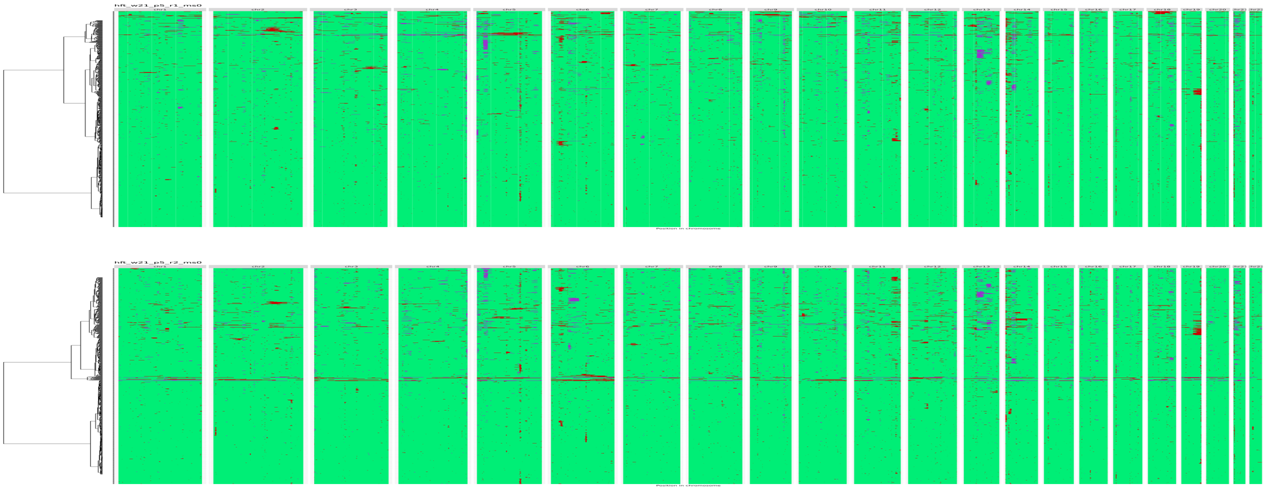

b)

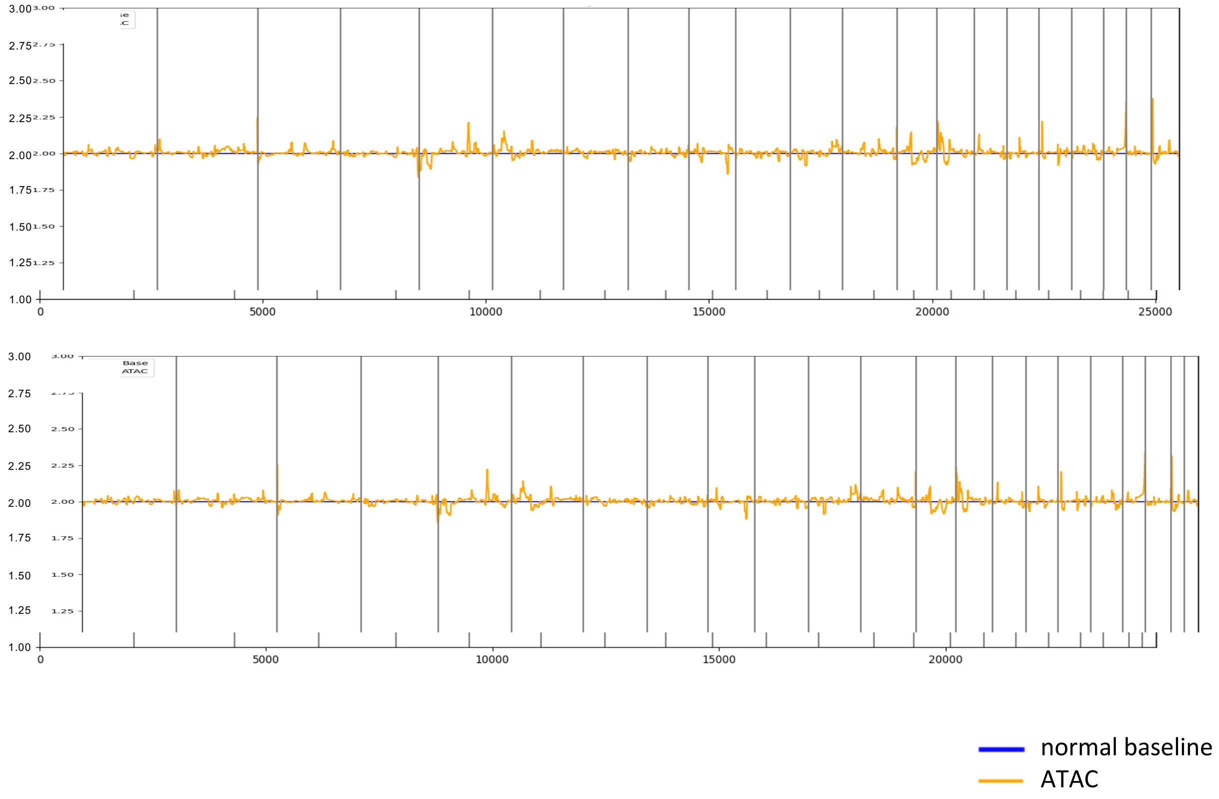

Figure S7: a) karyograms for two replicate datasets (replicates 1 and 2) from human brain (Trevino et al. 2021) that show no reproducible copy number gains or losses in the population. b) aggregated copy number profiles for all cells, compared to an euploid baseline set at the value of 2.

Figure S7: euploid samples

c)

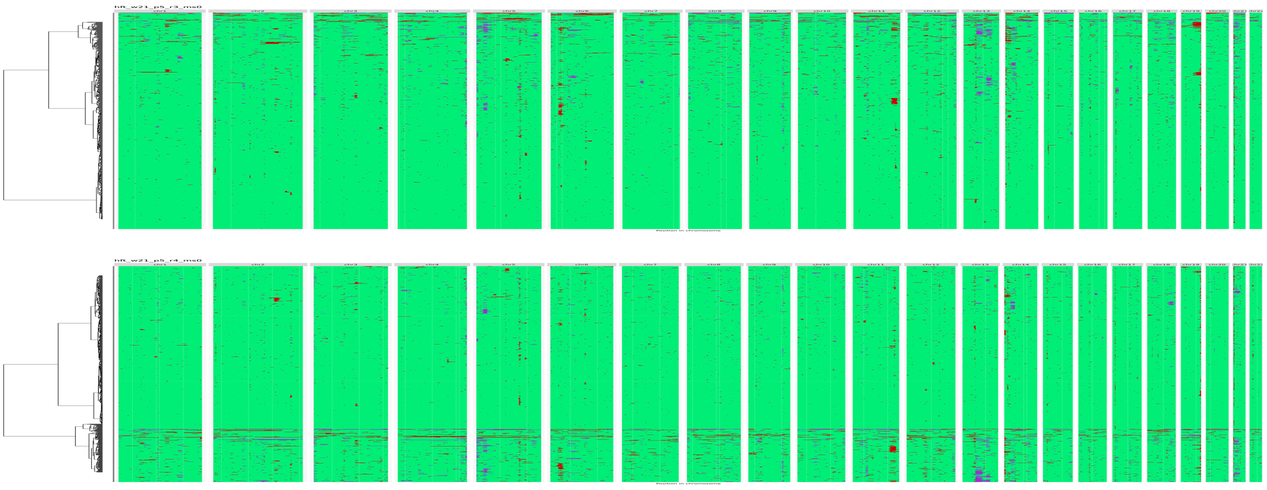

d)

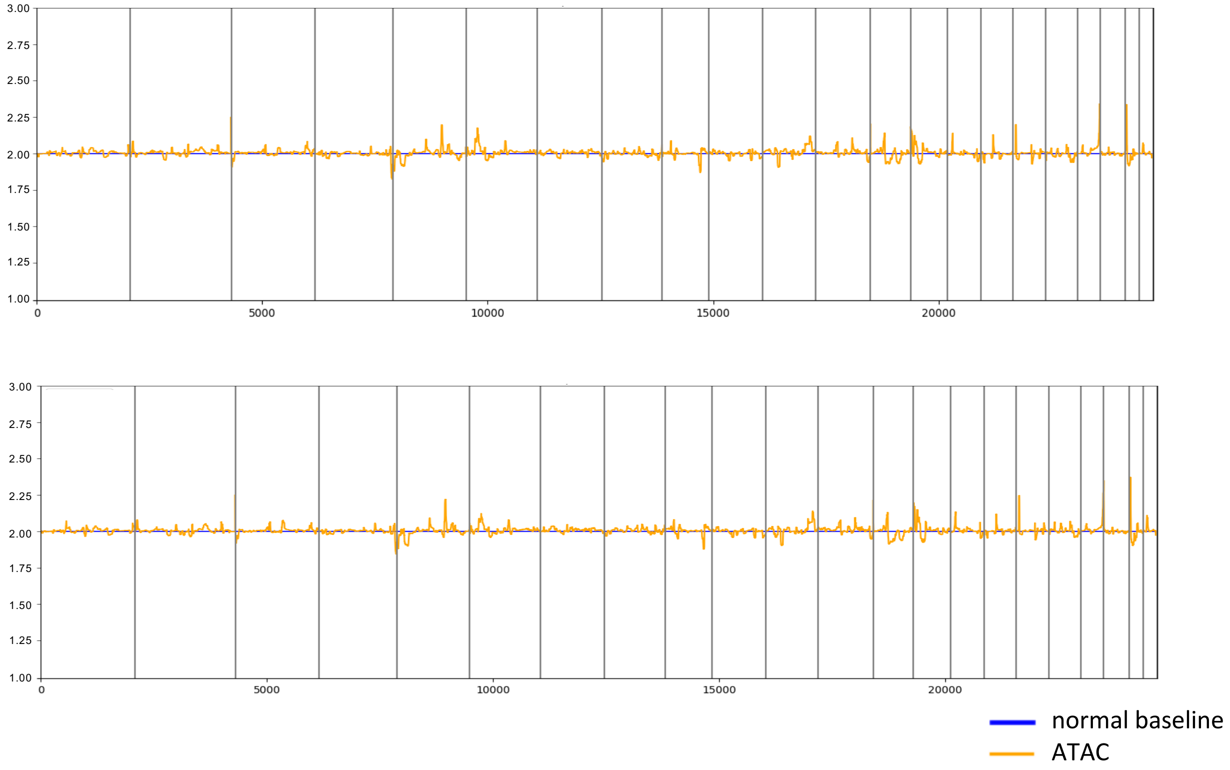

Figure S7: c) karyograms for two replicate datasets (replicates 3 and 4) from human brain (Trevino et al. 2021) that show no reproducible copy number gains or losses in the population. d) aggregated copy number profiles for all cells, compared to an euploid baseline set at the value of 2.

Figure S7: euploid samples

e)

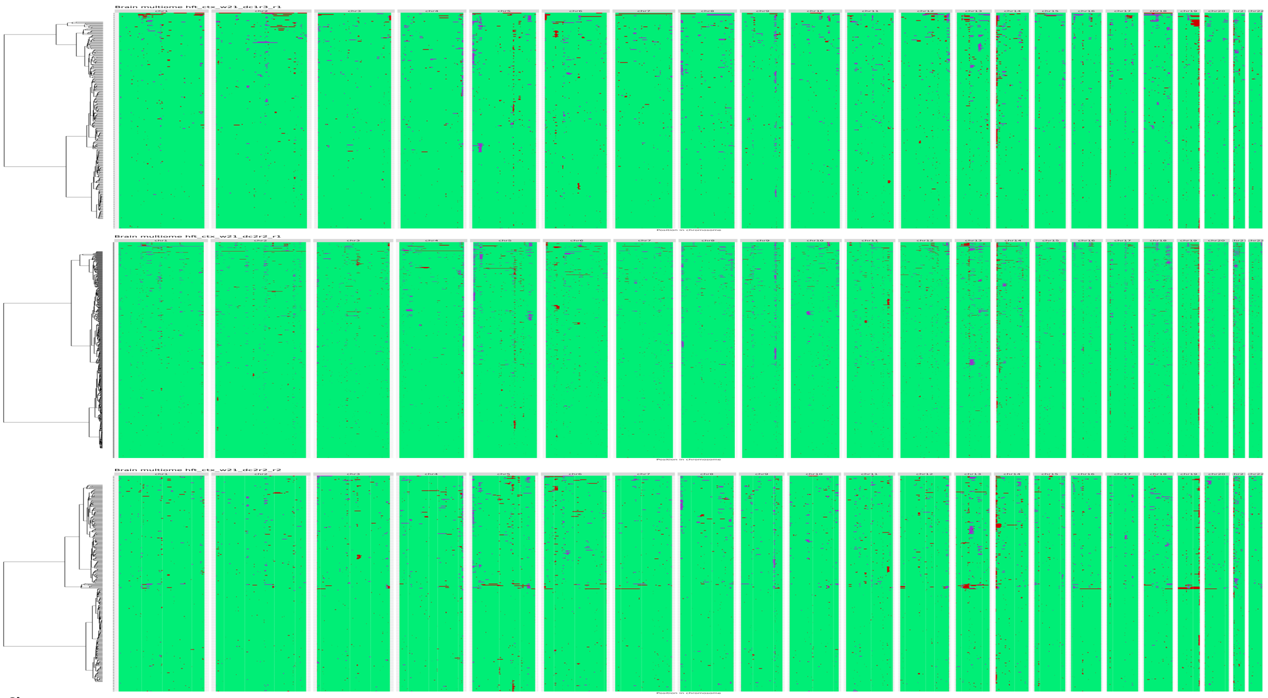

f)

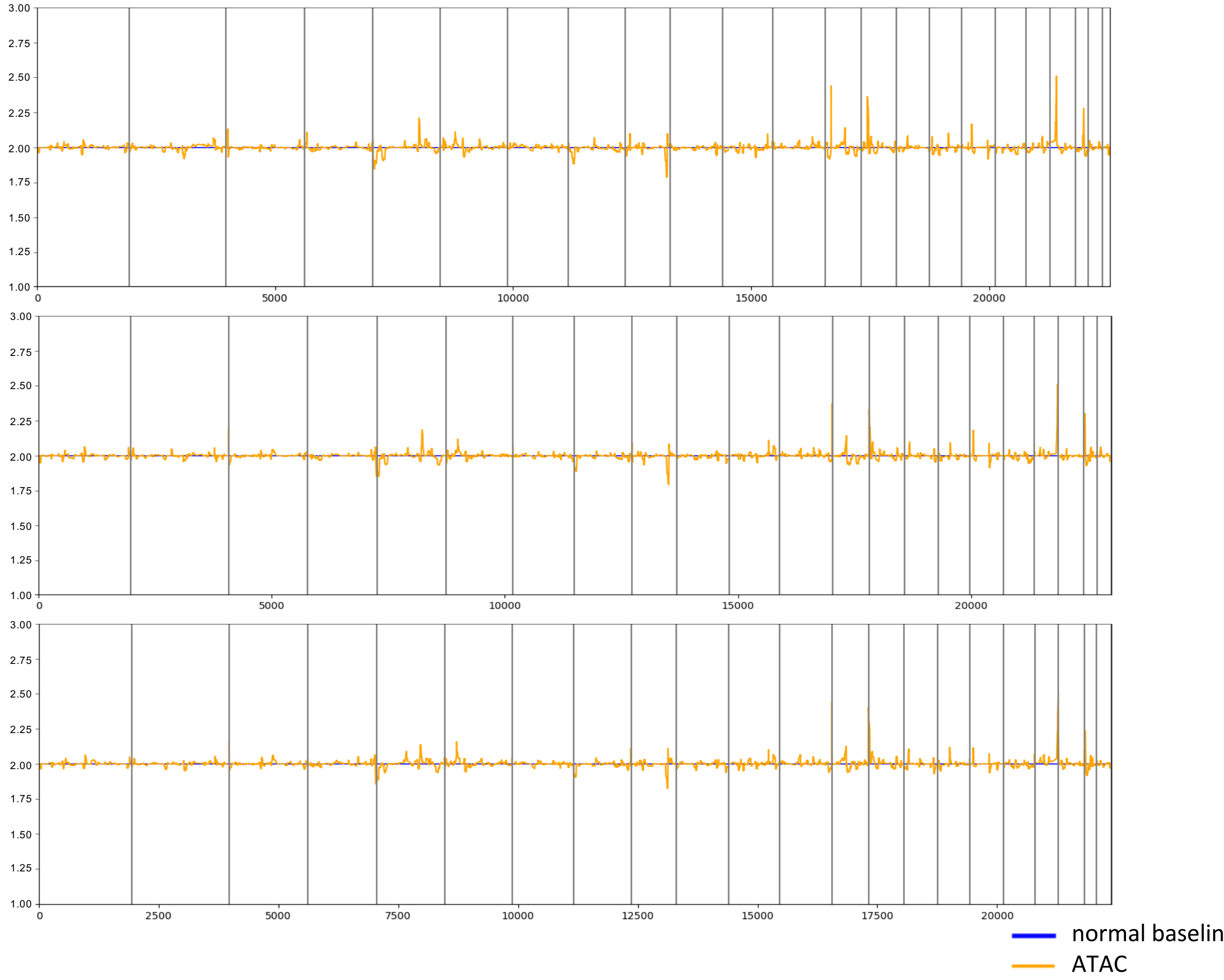

Figure S7: e) karyograms for three replicate datasets from human brain (Trevino et al. 2021), measured using single-cell multi-ome, that show no reproducible copy number gains or losses in the population. f) aggregated copy number profiles for all cells, compared to an euploid baseline set at the value of 2.

Figure S7: euploid samples

g)

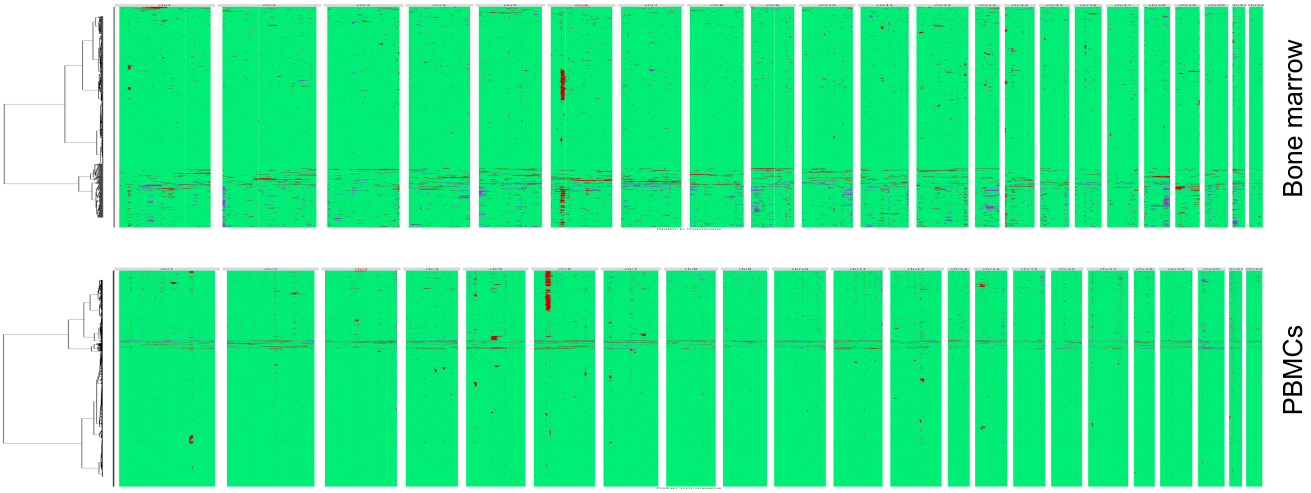

h)

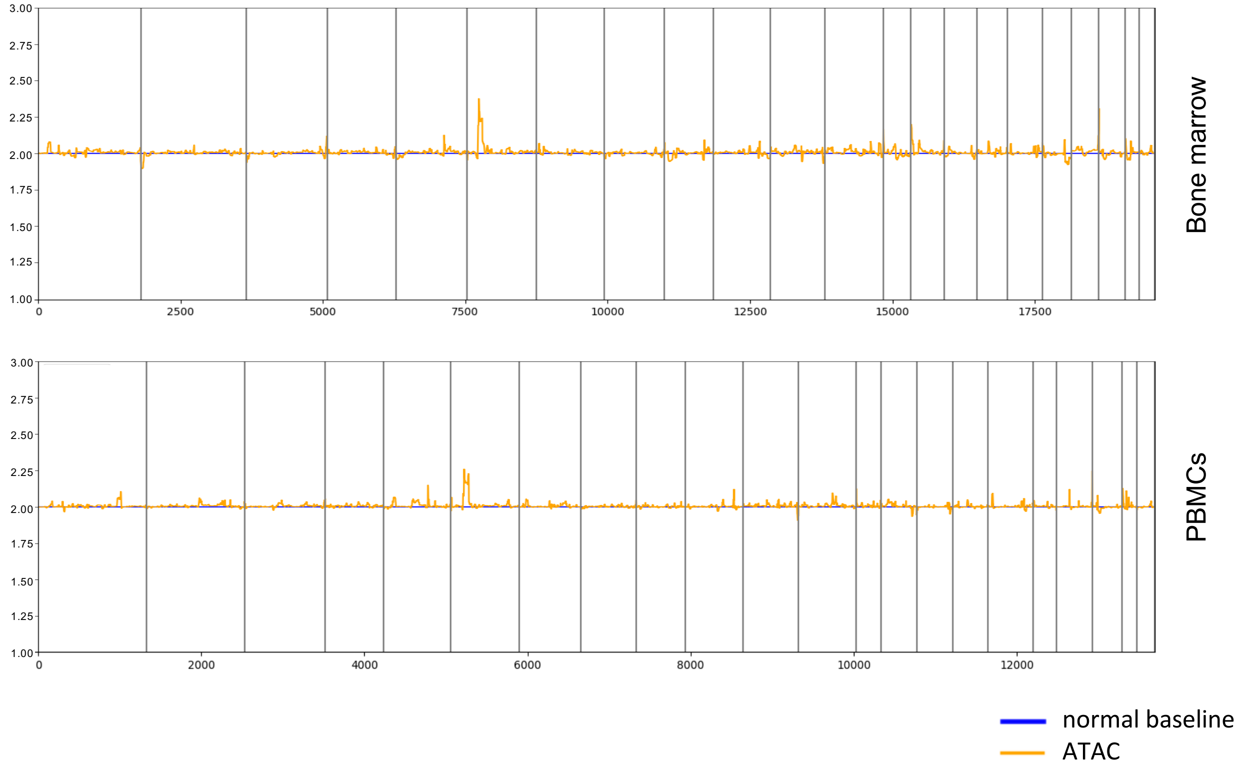

Figure S7: g) karyograms for a bone marrow (top) and a PBMC (bottom) human sample (Satpathy et al. 2019), that show no reproducible copy number gains or losses in the population. h) aggregated copy number profiles for all cells, compared to an euploid baseline set at the value of 2.

Figure S8: Comparison to scRNA-seq CNV callers and Copy-scAT

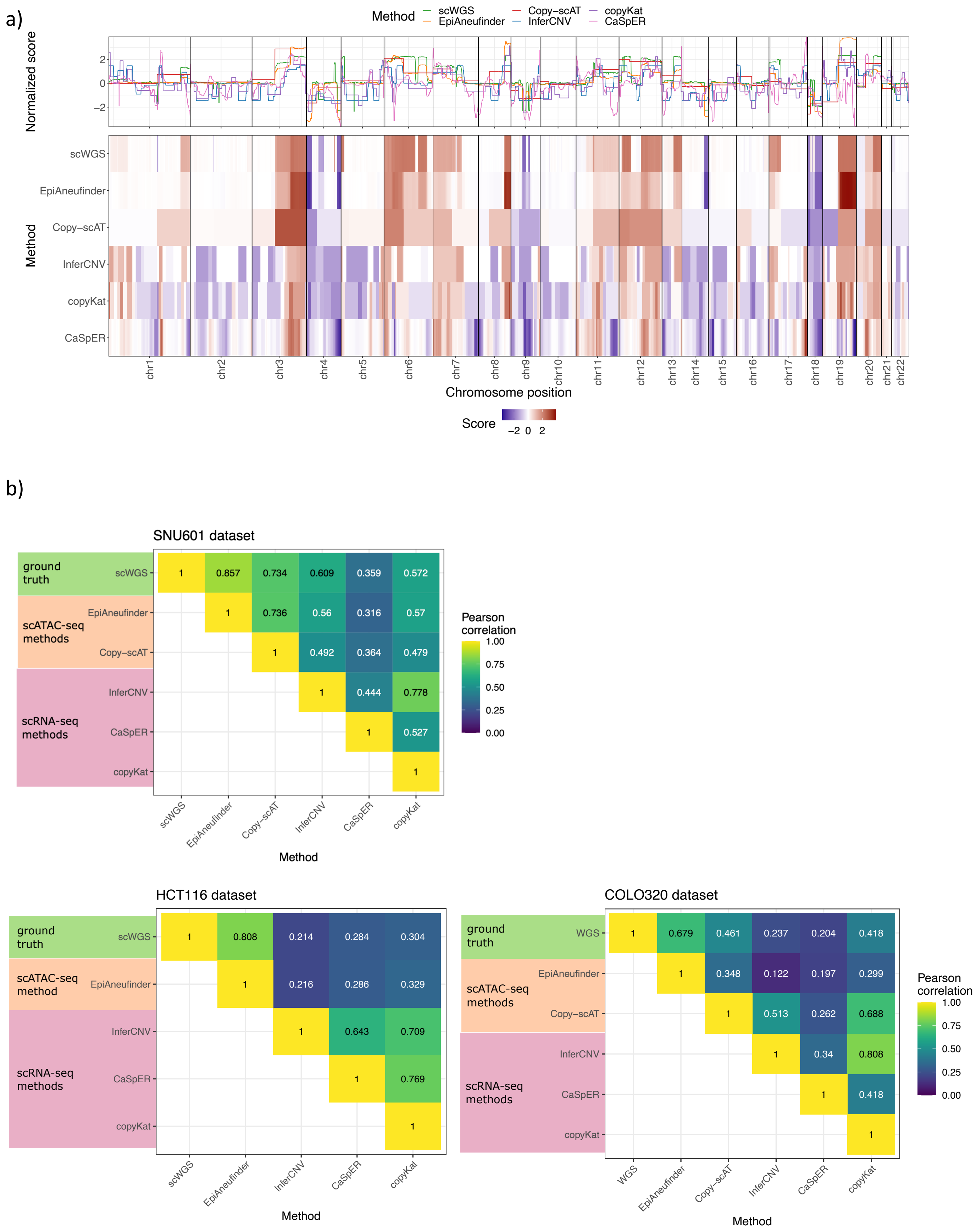

Figure S8: Comparison to scRNA-seq CNV callers and Copy-scAT

c)

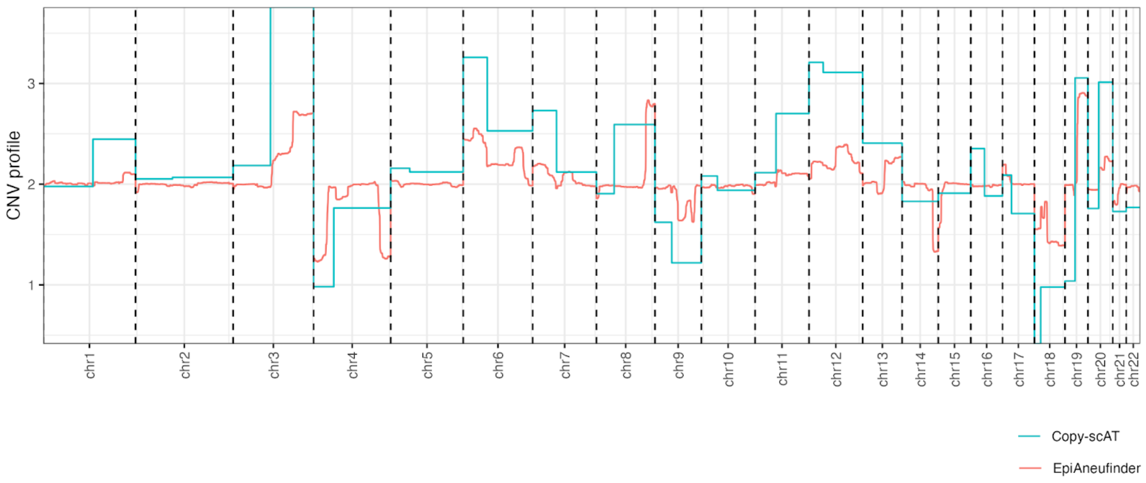

Figure S8: a) example output for every one of the tested CNV methods (scRNA-seq: inferCNV-copyKat, CaSpER; scATAC-seq: epiAneufinder, Copy-scAT) tested on the SNU601 cell line. A high score indicates gains and a low score losses. b) genome-wide correlation results for the comparisons of all outputs among them (pseudobulk correlations). Copy-scAT was not used to calculate CNVs in the HCT116 cell line as that data was not produced using the 10x technology. c) example output (pseudobulk profile) for epiAneufinder vs Copy-scAT for the SNU601 dataset.

Figure S9: Karyotypes for the SNU601 cell line when downsampling the number of reads

a)

100%

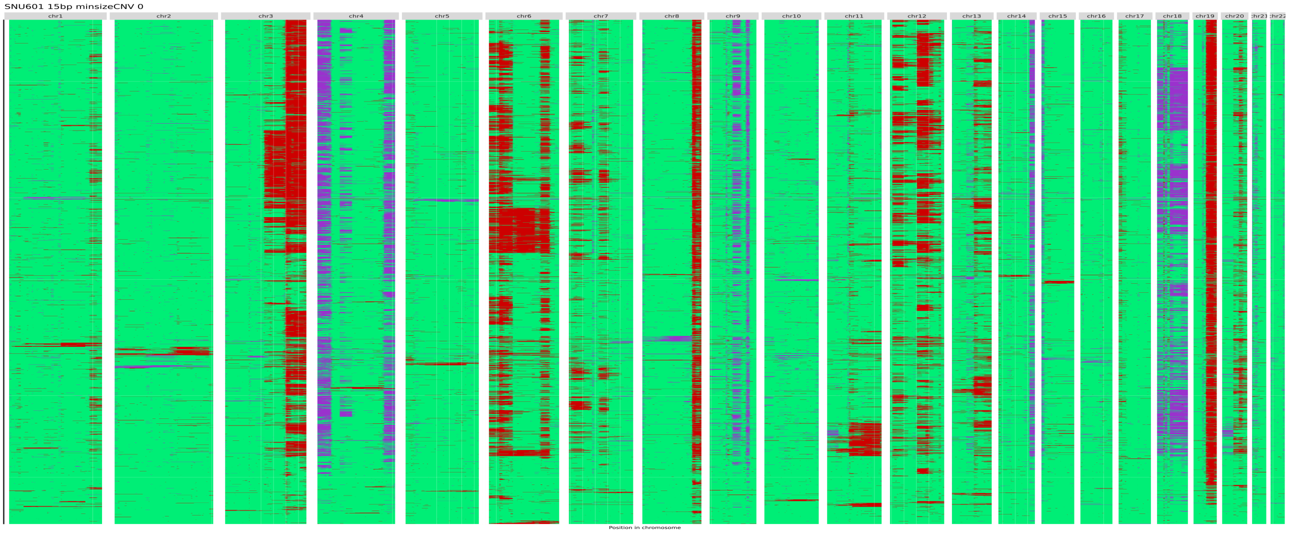

90%

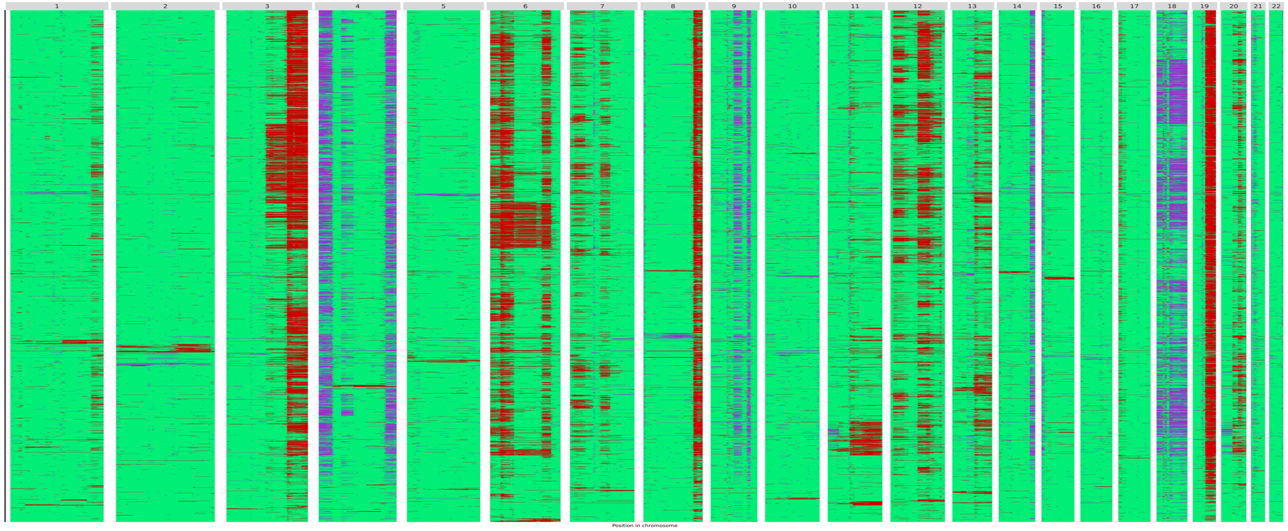

80%

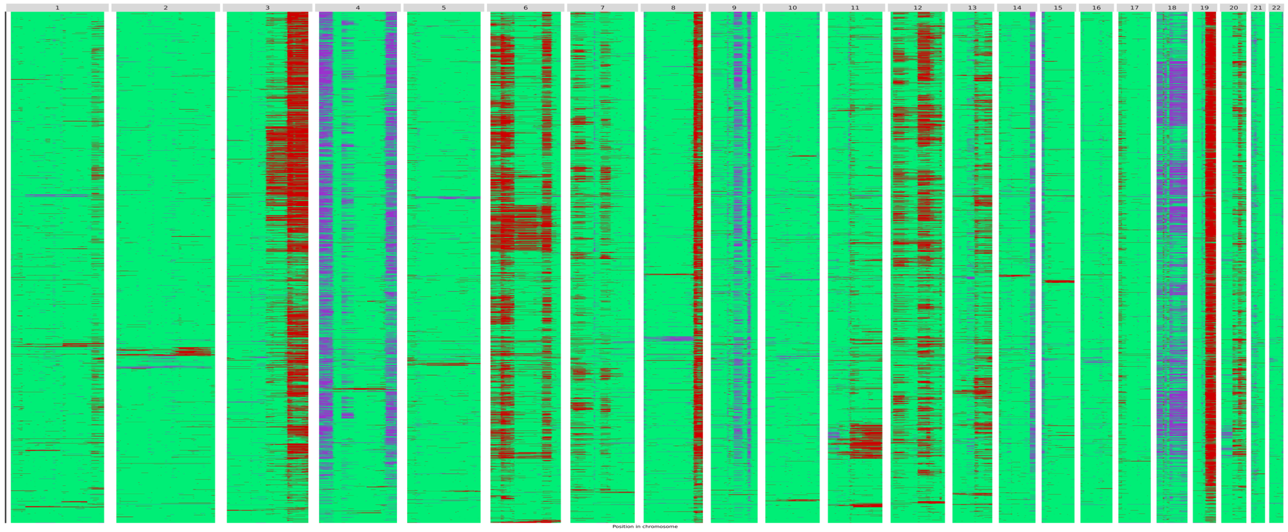

Figure S9: Karyotypes for the SNU601 cell line when downsampling the number of reads

70%

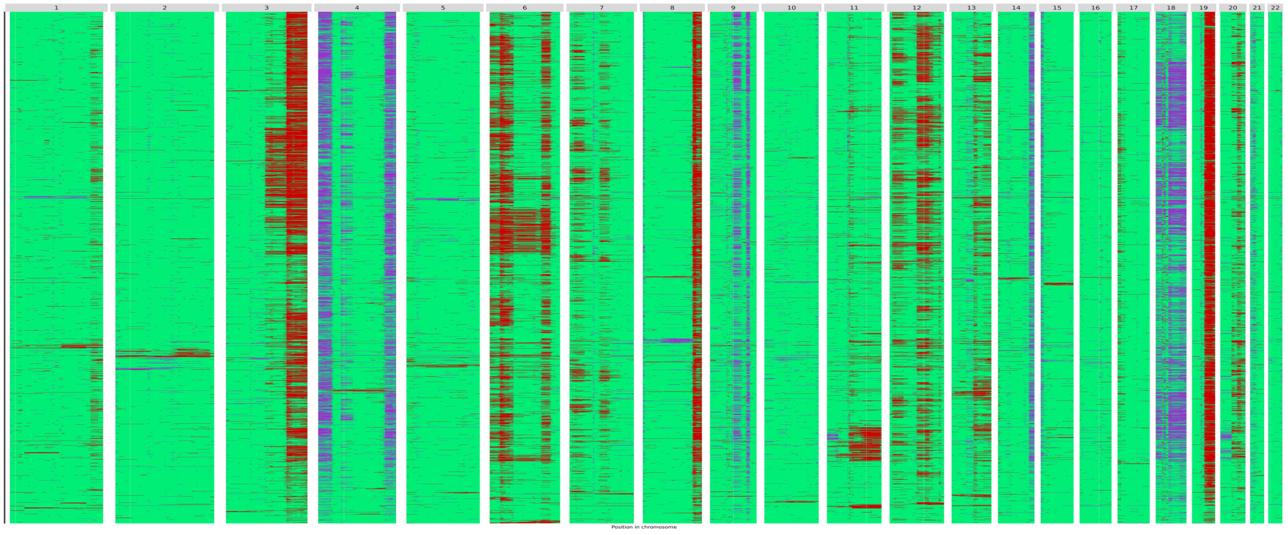

60%

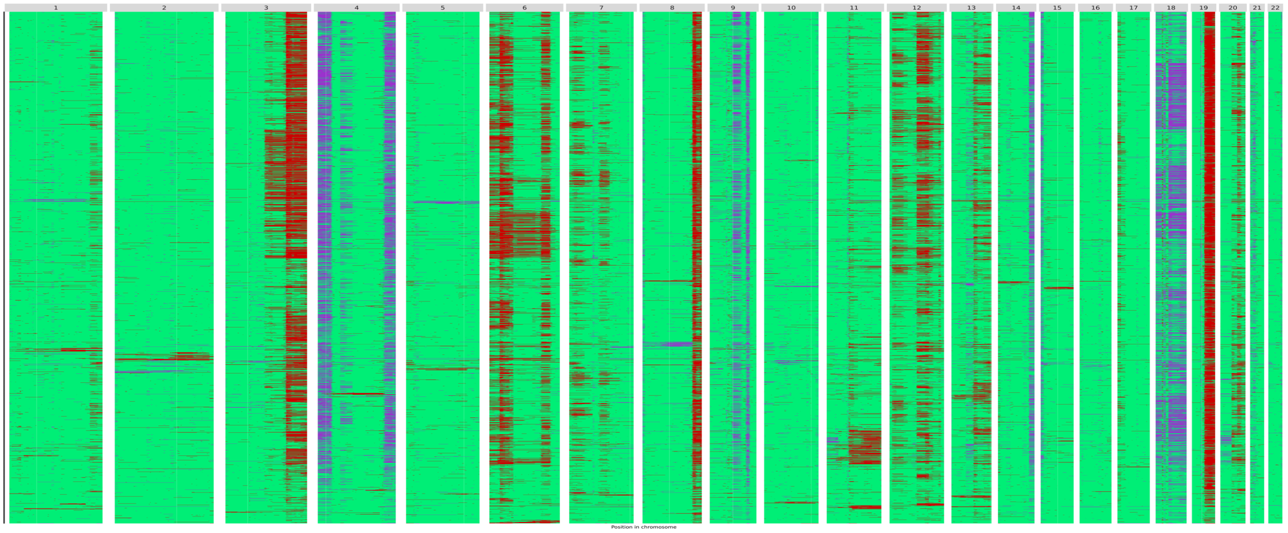

50%

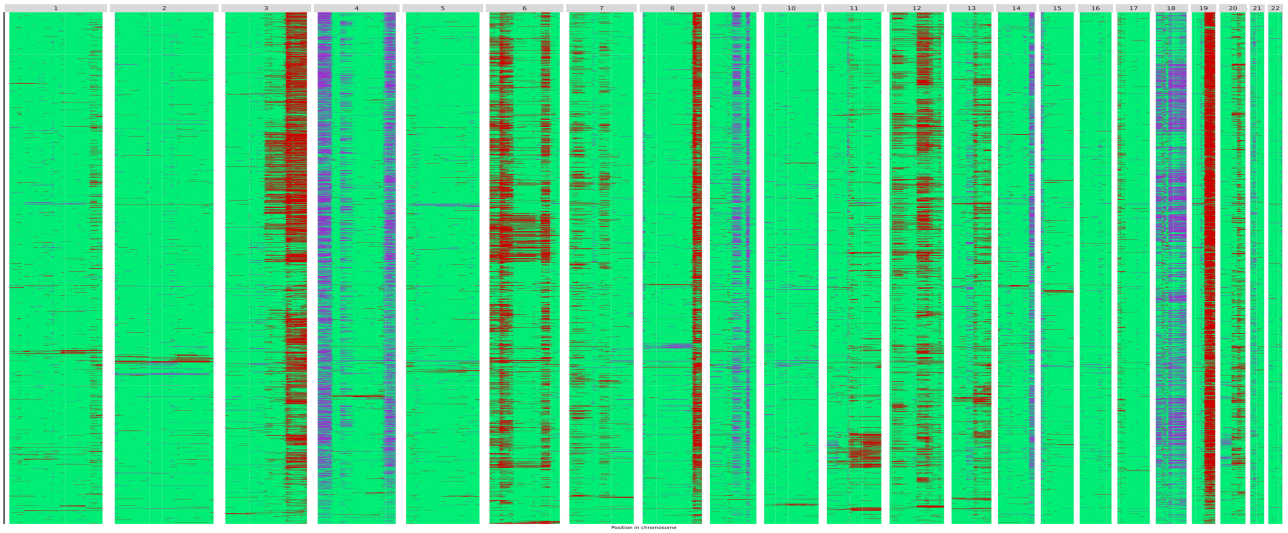

Figure S9: Karyotypes for the SNU601 cell line when downsampling the number of reads

40%

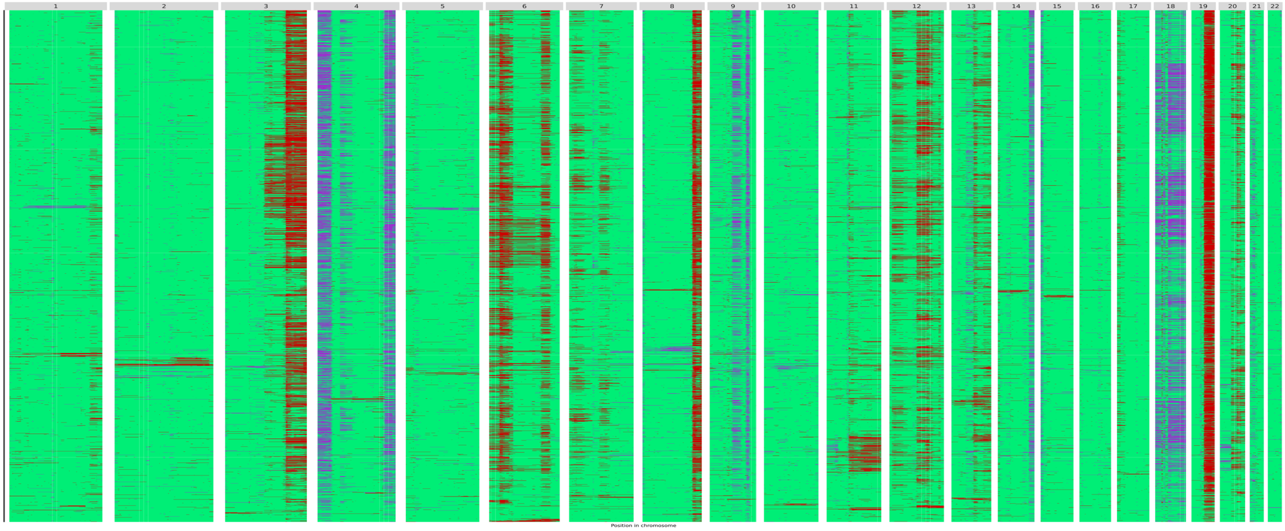

30%

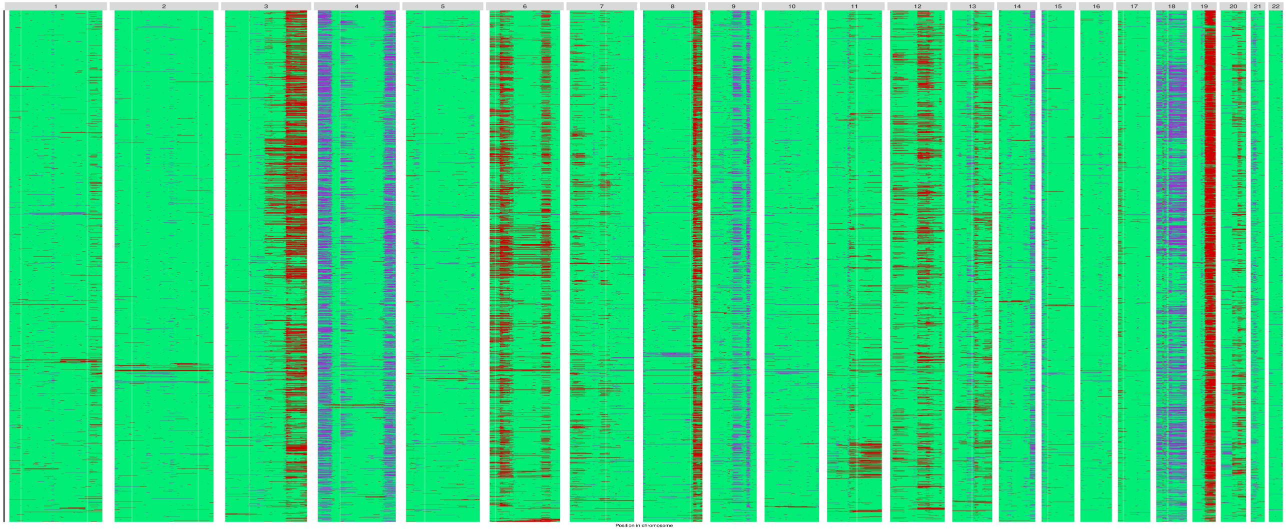

20%

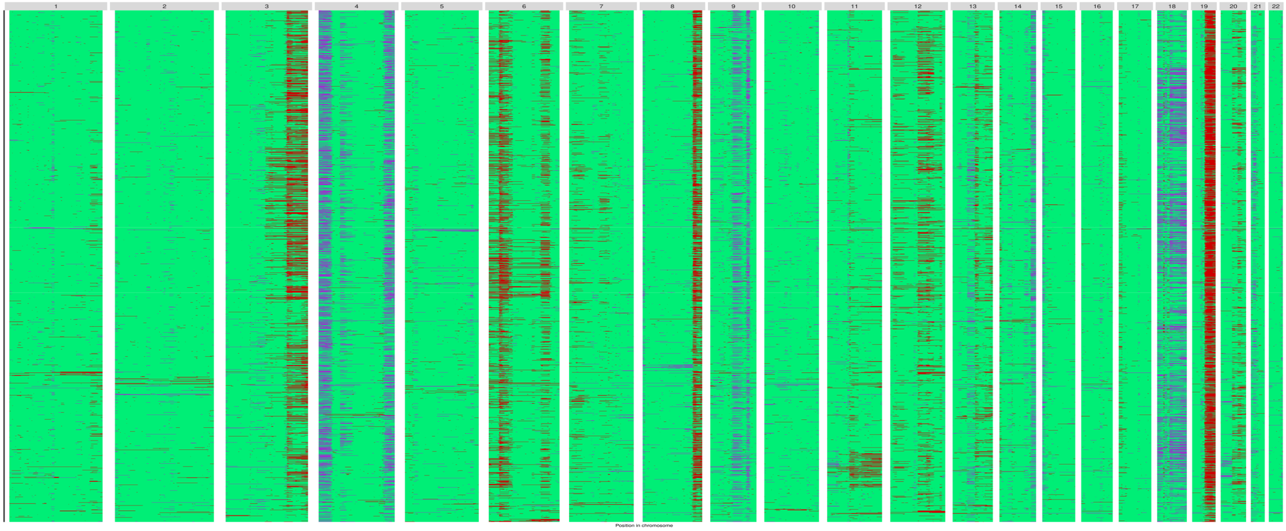

Figure S9: Karyotypes for the SNU601 cell line when downsampling the number of reads

10%

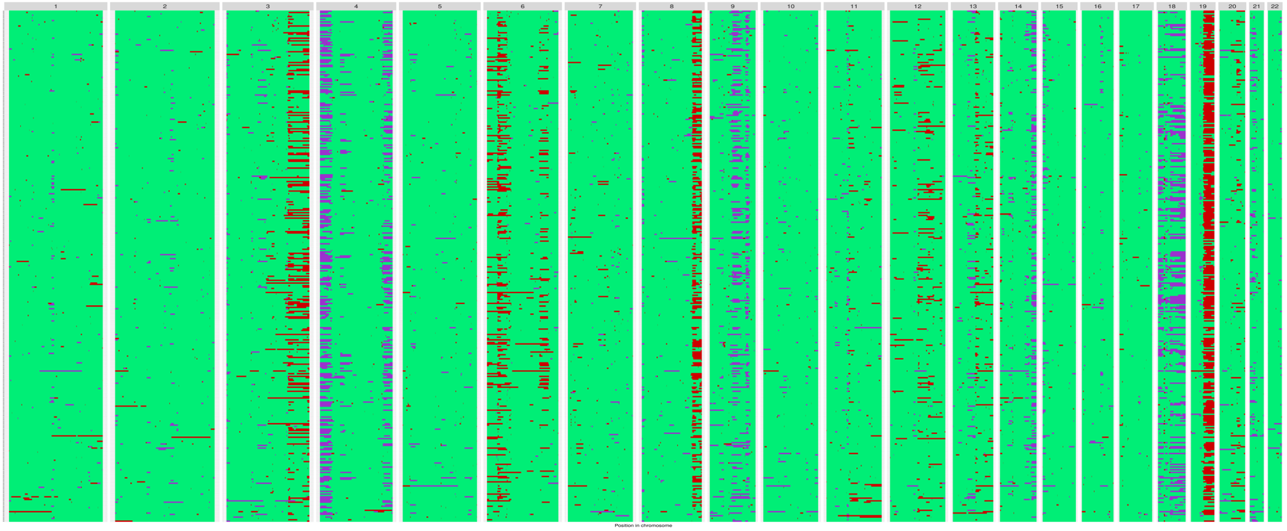

b)

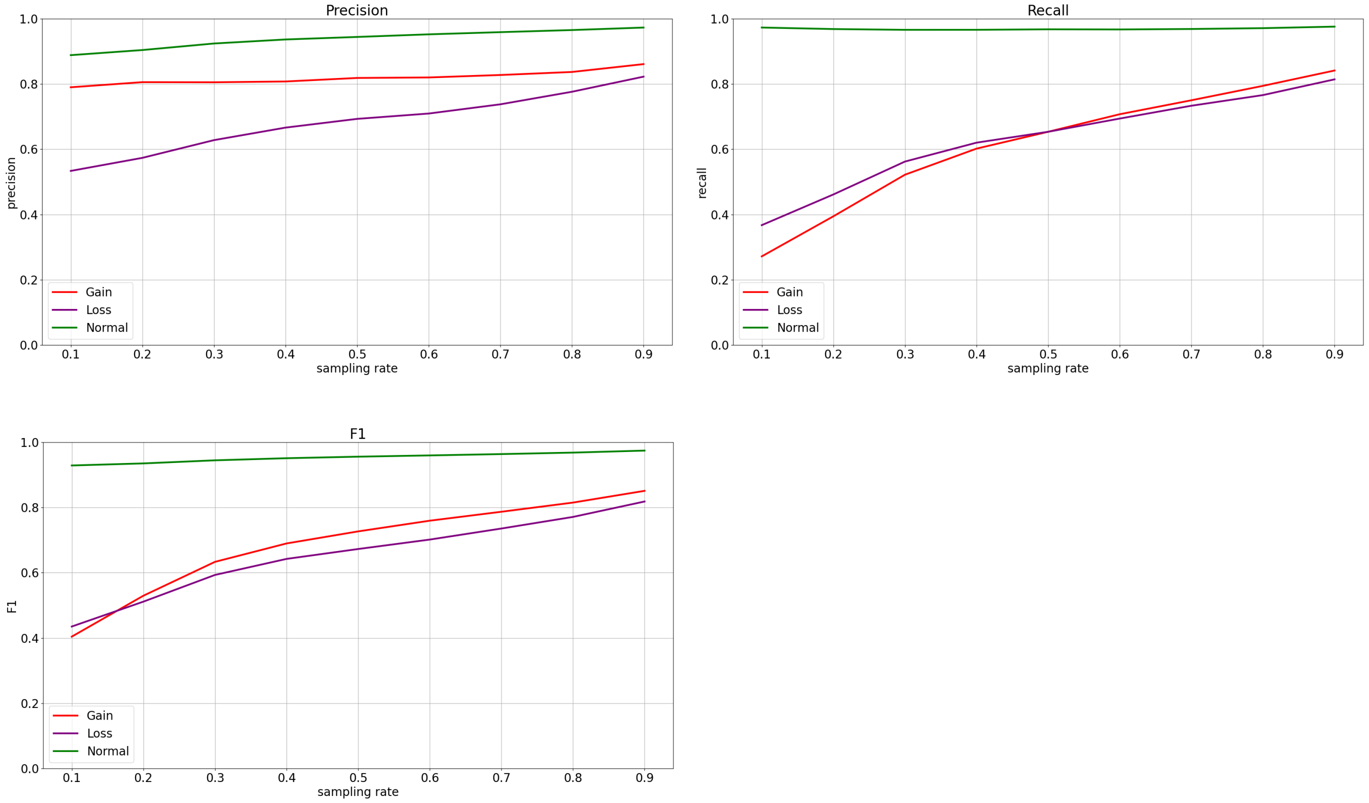

Figure S9: a) Karyograms for the results of the SNU601 cell line (Wu et al. 2021) when retaining only a percentage of the reads in the dataset (from 100% to 10%). The cells are always displayed in the same order. b) Precision, recall and F1 scores for every state, for the comparison between every downsampled dataset and the original 100% covered one.

Figure S10: SU006 patient sample

a)

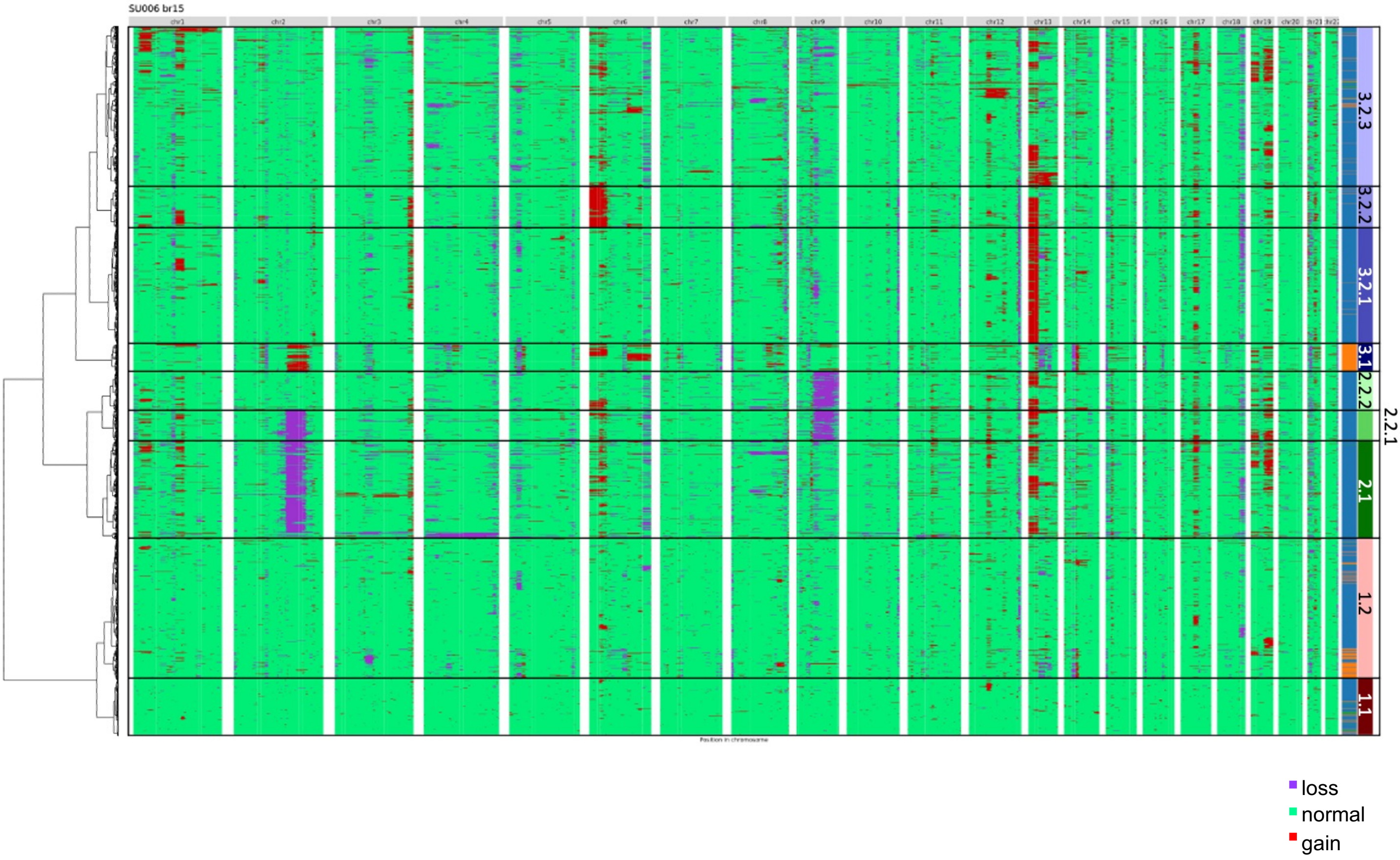

Figure S10: a) Karyogram per single-cell (every cell is a row, every column is a chromosome) for the SU006 patient sample (Satpathy et al. 2019), with gains indicated in red, losses in pruple and normal state in green. The cells are ordered based on the clustering results (clustering based on their copy number profiles using Euclidean distance and Ward Clustering). Karyotype clusters are indicated for branch cutting at depth 9. The colour bar represents cell types from b).

Figure S10: SU006 patient sample

b)

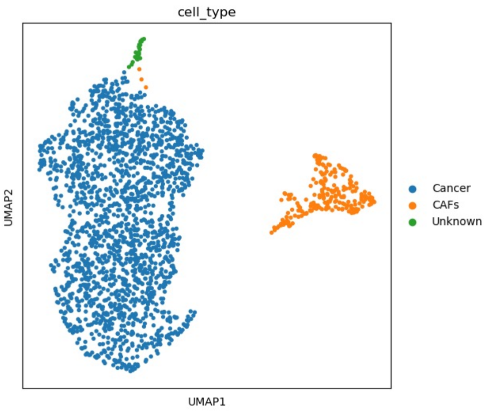

c)

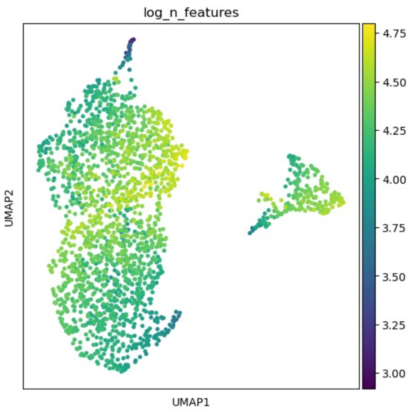

d)

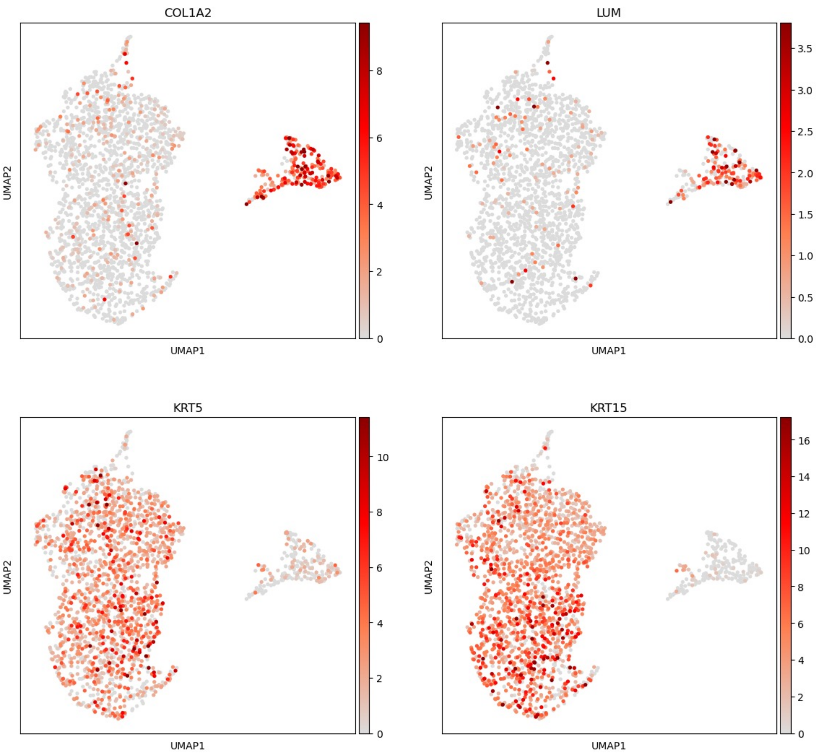

Figure S10: b) Embedding based on scATAC-seq data, cell types are indicated. c) Embedding based on scATAC-seq data, number of features are indicated, where it can be observed that the “unknown” cluster has a low number of features. d) gene activity levels for cancer associated fibroblast genes (COL1A2, LUM) and for basal cell marker genes (KRT5, KRT15).

Figure S10: SUO06 patient sample

e)

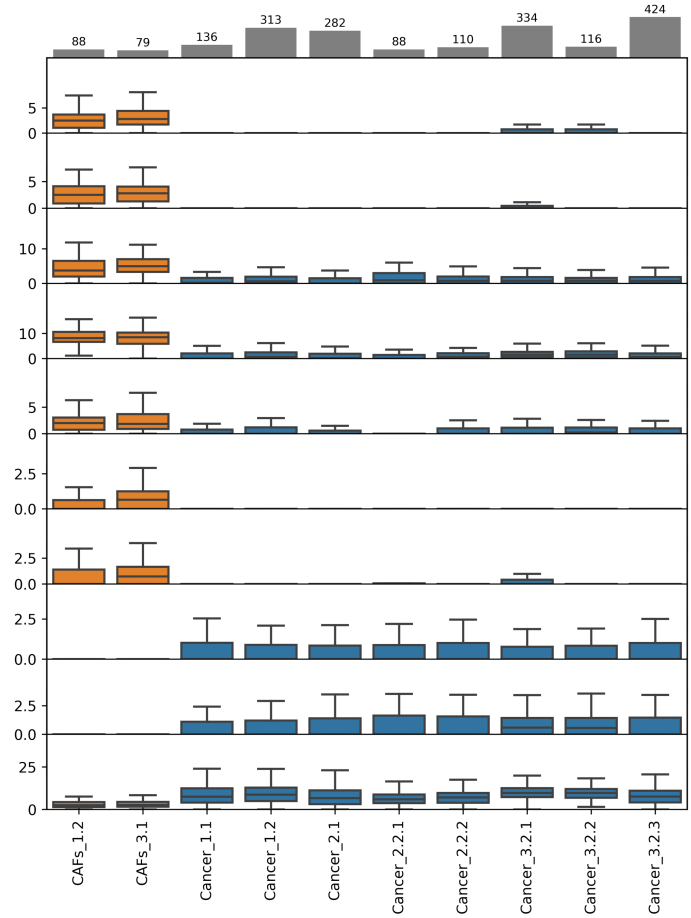

Figure S10: e) gene activity of the CAF and cancer associated marker genes per cluster (i.e. cell type) and per karyotype cluster. Indicated are only the cell types per cluster that make up at least 20% of the given karyotype. The boxplots represent with the horizontal line in the middle of the plot the median value. Lower and upper boundaries represent the first and third quartile, respectively. Largest and smallest observed values are shown (whisker lines).

Figure S10: SU006 patient sample

f)

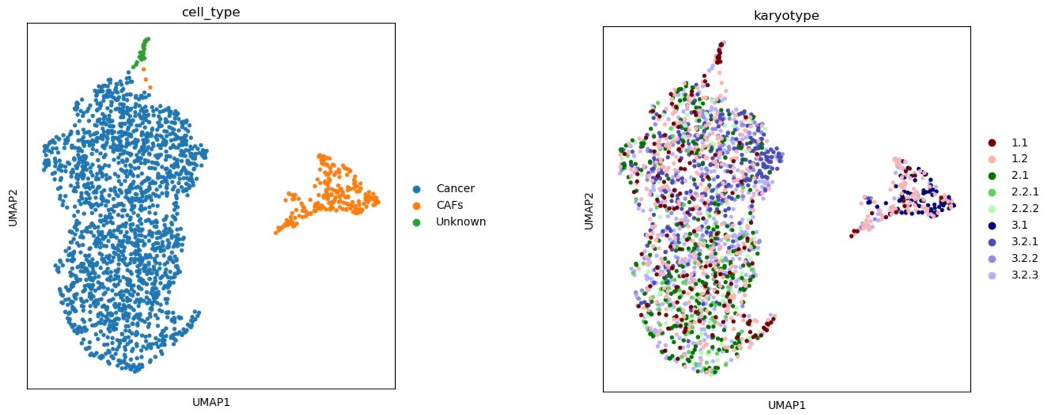

g)

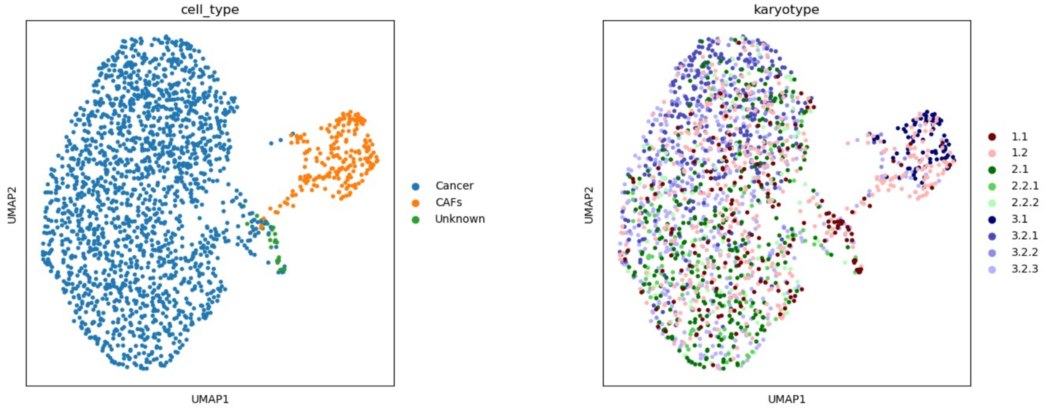

h)

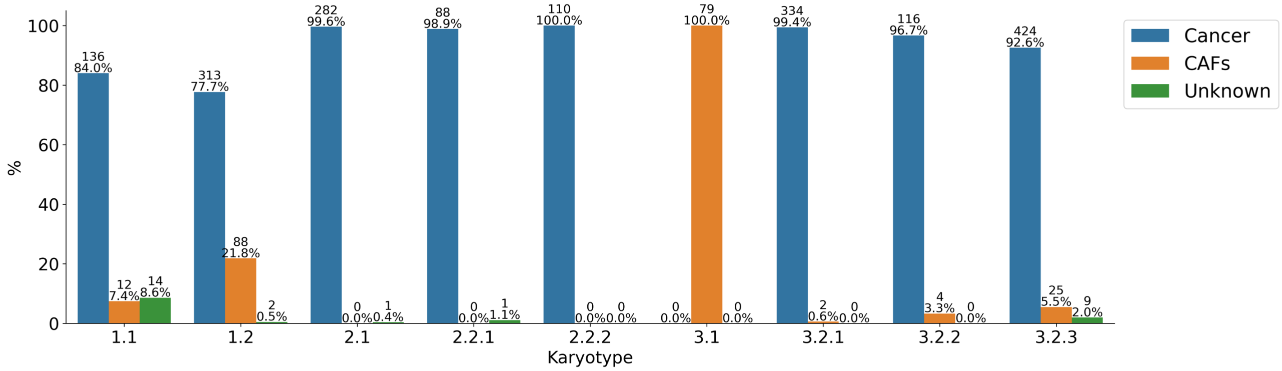

i)

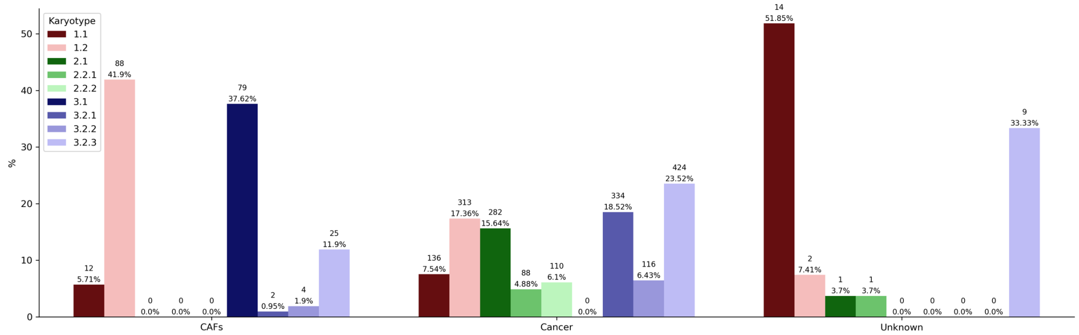

Figure S10: f) Embedding based on scATAC-seq peaks, with cell types (left) and karyotype clusters (right). g) Embedding based on scATAC-seq windows (same 100,000bp windows as used for karyotyping), with cell types (left) and karyotype clusters (right). h) Correspondence between the cells in every karyotype clusters and Leiden cluster. i) Correspondence between the cells in every Leiden cluster and karyotype clusters.

Figure S11: SU008 patient sample

a)

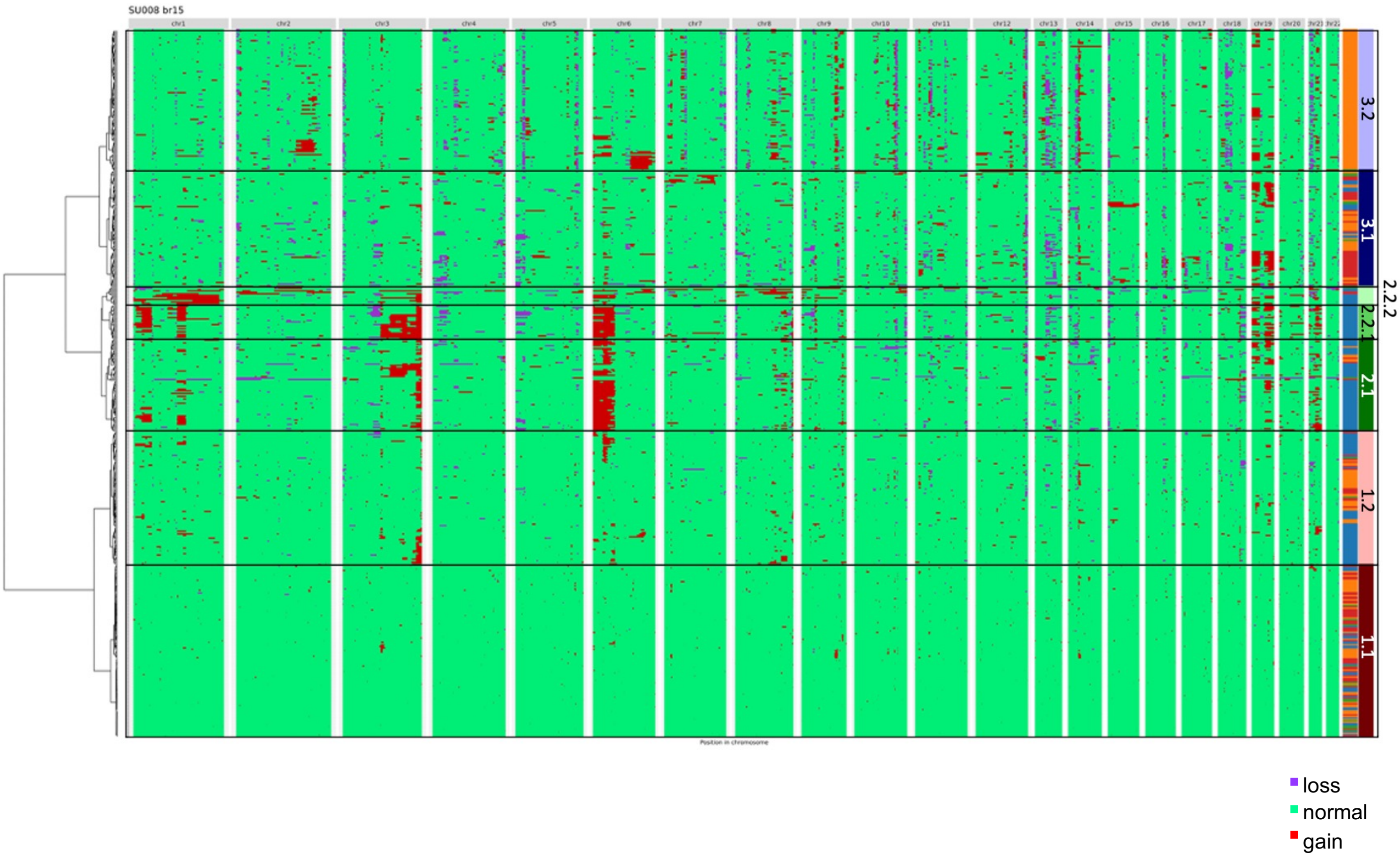

Figure S11: a) Karyogram per single-cell (every cell is a row, every column is a chromosome) for the SU008 patient sample (Satpathy et al. 2019), with gains indicated in red, losses in pruple and normal state in green. The cells are ordered based on the clustering results (clustering based on their copy number profiles using Euclidean distance and Ward Clustering). Karyotype clusters are indicated for branch cutting at depth 7. The colour bar represents cell types from b).

Figure S11: SU008 patient sample

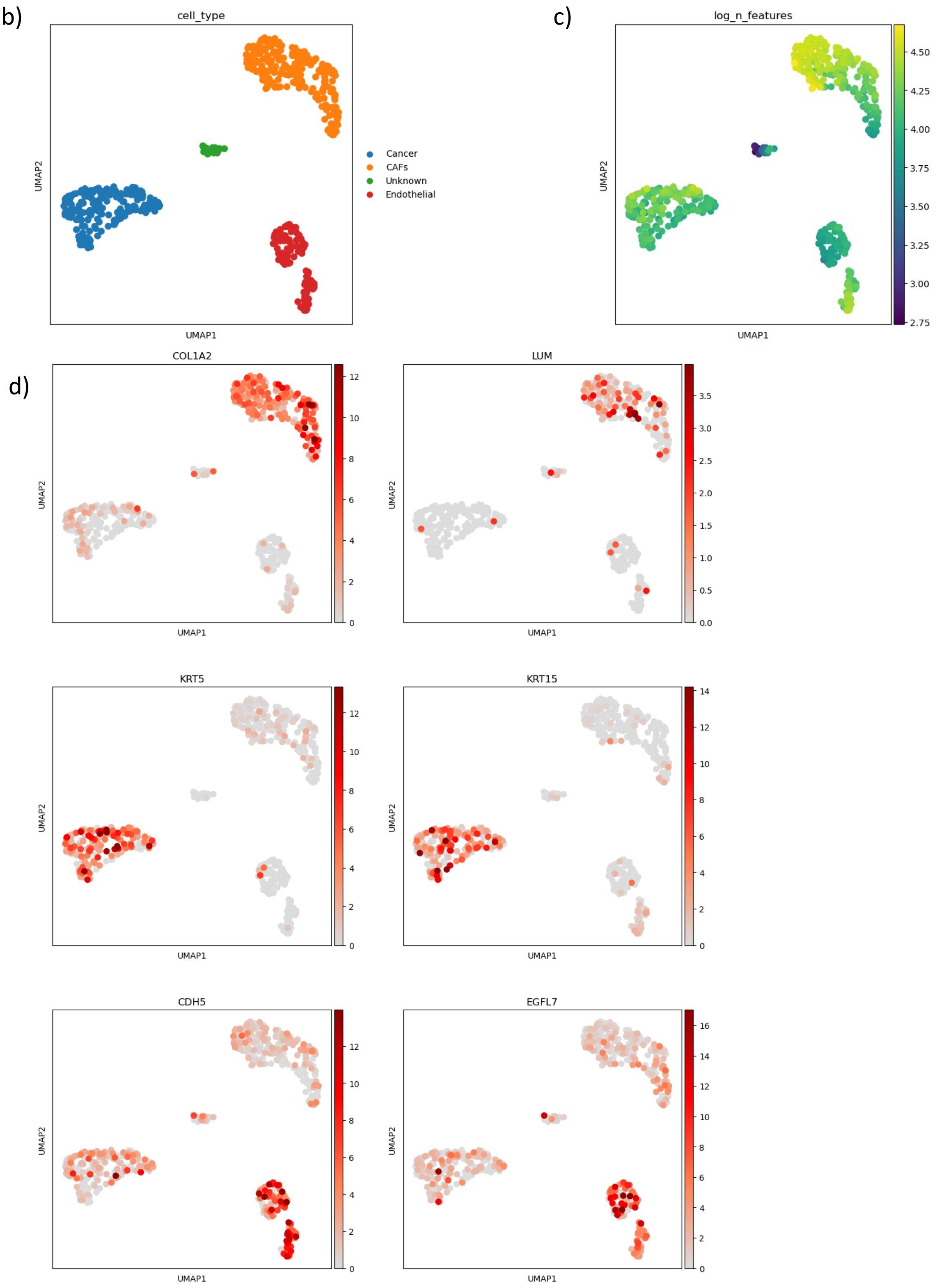

Figure S11: b) Embedding based on scATAC-seq data, cell types are indicated. c) Embedding based on scATAC-seq data, number of features are indicated, where it can be observed that the "unknown" cluster has a low number of features. d) Gene activity levels for cancer associated fibroblast genes (COL1A2, LUM), basal cell marker genes (KRT5, KRT15) and endothelial cell marker genes (CDH5, EGFL7).

Figure S11: SU008 patient sample

e)

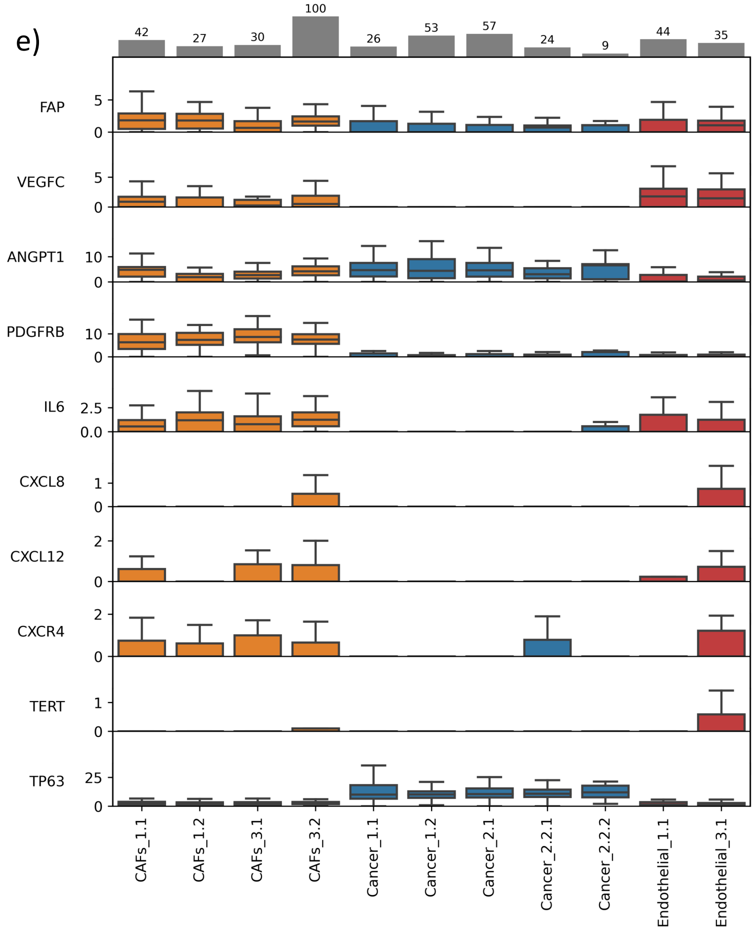

Figure S11: e) gene activity of the CAF and cancer associated marker genes per cluster (i.e. cell type) and per karyotype cluster. Indicated are only the cell types per cluster that make up at least 20% of the given karyotype. The boxplots represent with the horizontal line in the middle of the plot the median value. Lower and upper boundaries represent the first and third quartile, respectively. Largest and smallest observed values are shown (whisker lines).

Figure S11: SU008 patient sample

f)

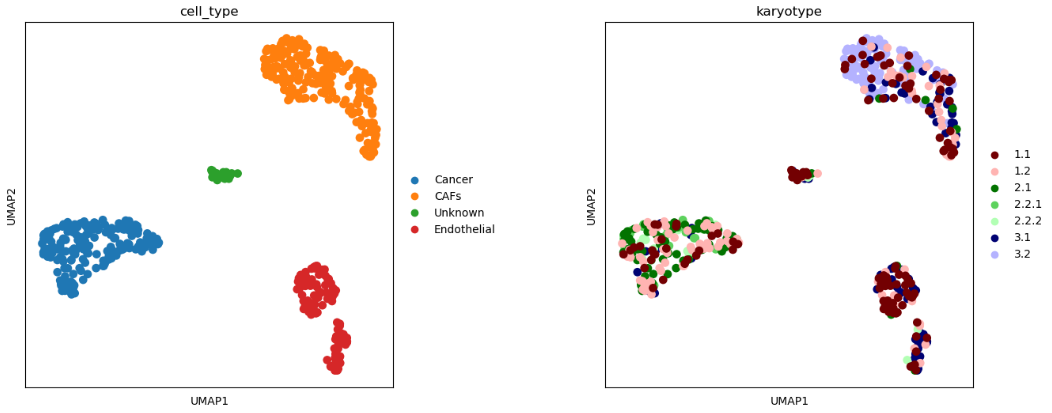

g)

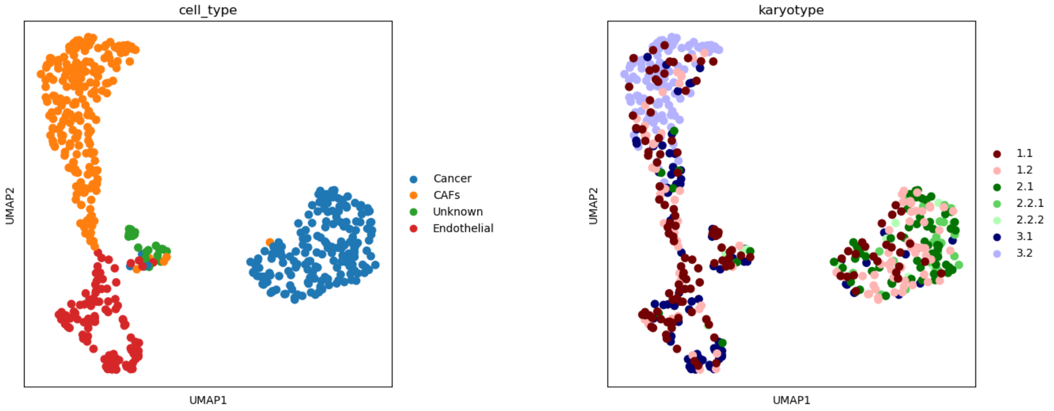

h)

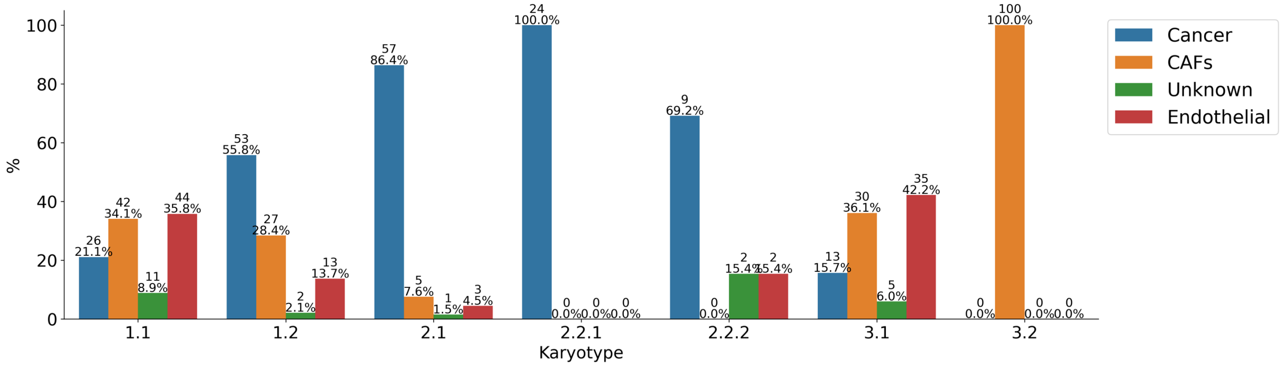

i)

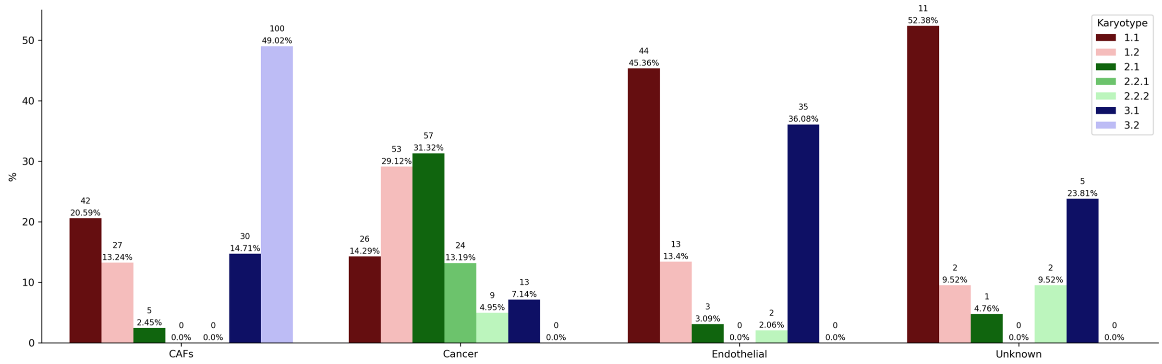

Figure S11: f) Embedding based on scATAC-seq peaks, with cell types (left) and karyotype clusters (right). g) Embedding based on scATAC-seq windows (same 100,000bp windows as used for karyotyping), with cell types (left) and karyotype clusters (right). h) Correspondence between the cells in every karyotype cluster and Leiden cluster. i) Correspondence between the cells in every Leiden cluster and karyotype clone.

Table S1: Runtime

Number 0f breakpoints	RAM Usage	CPU wall time
3	159.27GB	13:55:51
7	160.16 GB	17:43:38
15	168.32GB	22:02:56
31	174.89GB	35:53:38

Sup. Table 1 : CPU wall time and memory usage of the SNU601 dataset (3157 cells), using different number of breakpoints. For all datasets a single node in the cluster was used, with 6 cores and 192Gb RAM. For the wall time calculations we used all the steps of the epiAneufinder pipeline.

Table S2: Mean square error for the SNU601 sample

	Mean Square Error
genome	0,09365611
chr1	0,04145181
chr2	0,00239785
chr3	0,08465411
chr4	0,09371863
chr5	0,10027005
chr6	0,29434402
chr7	0,13314193
chr8	0,01518072
chr9	0,10345382
chr10	0,00205928
chr11	0,05815861
chr12	0,26920566
chr13	0,21350414
chr14	0,03350693
chr15	0,00503614
chr16	0,00749366
chr17	0,12447185
chr18	0,13280346
chr19	0,0942314
chr20	0,17696876
chr21	0,02750322
chr22	0,00451012

Sup. Table 2: Mean square error for the comparison between the copy number calls from scWGS and scATAC-seq in the SNU601 cell line.

Table S3: Mean square error for the HCT116 sample

	Mean Square Error
genome	0,02542377
chr1	0,01630795
chr2	0,02504493
chr3	0,0077424
chr4	0,01383782
chr5	0,01150972
chr6	0,00579021
chr7	0,02742496
chr8	0,08324881
chr9	0,00920415
chr10	0,07265785
chr11	0,03635834
chr12	0,01035052
chr13	0,00554879
chr14	0,00784719
chr15	0,03865515
chr16	0,0711472
chr17	0,06564807
chr18	0,00659765
chr19	0,01173866
chr20	0,00254783
chr21	0,0414797
chr22	0,03232457

Sup. Table 3: Mean square error for the comparison between the copy number calls from scWGS and scATAC-seq in the HCT116 cell line.

Table S4: Recall, variation, and mean square error for all the euploid samples when compared to a “normal” baseline

Sample	Recall	Variation	MSE
Satpathy_BoneMarrow	0,97459945	0,01251366	0,00067758
Satpathy_PBMC_rep1	0,98485799	0,00921701	0,00038248
Greenleaf_brain_rep1	0,97002464	0,01485453	0,00093185
Greenleaf_brain_rep2	0,97008819	0,01436002	0,00085472
Greenleaf_brain_rep3	0,9710737	0,01440061	0,00085159
Greenleaf_brain_rep4	0,97130869	0,01458655	0,00088512
multiome_brain_rep1	0,98151908	0,01408168	0,00079308
multiome_brain_rep2	0,98316854	0,013363	0,00071464
multiome_brain_rep3	0,98261042	0,01374836	0,00075774

Sup. Table 4: Recall, variation, and mean square error for all the euploid samples when comparing the pseudo bulk aggregates to a reference line of “normal” (value of 2).

Table S5: Downsampling of the SNU601 dataset

SNU601 Percent coverage	cells	fragments per cell	Mean read pairs per cell	Sequenced read pairs
1	3575	75013	147358.63	526807107
0.9	3547	70117	132921.31	474130324
0.8	3555	64738	118548.79	421440958
0.7	3541	58944	104138.51	368754462
0.6	3534	52679	89439.96	316080807
0.5	3527	45843	74679.96	263396204
0.4	3528	38420	59725.61	210711941
0.3	3502	30298	45124.65	158026510
0.2	3462	21323	30430.35	105349877
0.1	3326	11447	15838.18	52677780

Sup. Table 5: Results from downsampling the SNU601 cell line dataset.

Table S6: Downsampling of the SNU601 dataset

SNU601 Percent coverage	cells after filtering	bins after filtering
1	3153	26557
0.9	3119	26543
0.8	3085	26540
0.7	3039	26523
0.6	2973	26509
0.5	2879	26495
0.4	2474	26478
0.3	2485	26439
0.2	1907	26308
0.1	377	26096

Sup. Table 6: Number of cells and bins that were retained after the filtering step of epiAneufinder for the different downsampling percentages.

Table S7: Precision, recall and F1 for the downsampled datasets

	Precision			Recall			F1			
Percent coverage	Gain	Loss	Disomic	Gain	Loss	Disomic	Gain	Loss	Disomic	#cells
0,1	0,7901	0,5336	0,8886	0,2715	0,3673	0,9733	0,4041	0,4351	0,9290	378
0,2	0,8058	0,5736	0,9043	0,3944	0,4613	0,9686	0,5296	0,5114	0,9353	1908
0,3	0,8054	0,6280	0,9243	0,5220	0,5623	0,9662	0,6334	0,5933	0,9448	2486
0,4	0,8079	0,6664	0,9367	0,6020	0,6202	0,9663	0,6899	0,6425	0,9513	2745
0,5	0,8187	0,6932	0,9444	0,6536	0,6536	0,9678	0,7269	0,6728	0,9560	2880
0,6	0,8202	0,7095	0,9524	0,7075	0,6941	0,9674	0,7597	0,7018	0,9599	2974
0,7	0,8277	0,7378	0,9591	0,7502	0,7334	0,9688	0,7871	0,7356	0,9639	3040
0,8	0,8370	0,7763	0,9656	0,7944	0,7661	0,9714	0,8152	0,7712	0,9685	3086
0,9	0,8611	0,8226	0,9732	0,8415	0,8143	0,9760	0,8512	0,8185	0,9746	3120

Sup. Table 7: Precision, recall and F1 score of the gain/loss/disomic bins in the different downsampled fractions
