# Supplementary material for: epiAneufinder identifies copy number alterations from single-cell ATAC-seq data
Source: Nat Commun. 2023 Sep 20;14:5846. doi: 10.1038/s41467-023-41076-1 (PMC10511508; doi:10.1038/s41467-023-41076-1)
Supplement: Supplementary file 3 — Reporting Summary [file 41467_2023_41076_MOESM3_ESM.pdf]

nature portfolio

Corresponding author(s): Maria Colomé-Tatché

Last updated by author(s): Jun 7, 2023

Reporting Summary

Nature Portfolio wishes to improve the reproducibility of the work that we publish. This form provides structure for consistency and transparency in reporting. For further information on Nature Portfolio policies, see our Editorial Policies and the Editorial Policy Checklist.

Statistics

For all statistical analyses, confirm that the following items are present in the figure legend, table legend, main text, or Methods section.

n/a	Confirmed	
☐	☒	The exact sample size (*n*) for each experimental group/condition, given as a discrete number and unit of measurement
☐	☒	A statement on whether measurements were taken from distinct samples or whether the same sample was measured repeatedly
☐	☒	The statistical test(s) used AND whether they are one- or two-sided<br>*Only common tests should be described solely by name; describe more complex techniques in the Methods section.*
☒	☐	A description of all covariates tested
☒	☐	A description of any assumptions or corrections, such as tests of normality and adjustment for multiple comparisons
☐	☒	A full description of the statistical parameters including central tendency (e.g. means) or other basic estimates (e.g. regression coefficient) AND variation (e.g. standard deviation) or associated estimates of uncertainty (e.g. confidence intervals)
☐	☒	For null hypothesis testing, the test statistic (e.g. *F*, *t*, *r*) with confidence intervals, effect sizes, degrees of freedom and *P* value noted<br>*Give P values as exact values whenever suitable.*
☒	☐	For Bayesian analysis, information on the choice of priors and Markov chain Monte Carlo settings
☐	☒	For hierarchical and complex designs, identification of the appropriate level for tests and full reporting of outcomes
☐	☒	Estimates of effect sizes (e.g. Cohen's *d*, Pearson's *r*), indicating how they were calculated

Our web collection on statistics for biologists contains articles on many of the points above.

Software and code

Policy information about availability of computer code

Data collection	All data used were publicly available and downloaded from the respective databases (SRA, GEO,ENA). No software was used for data collection
Data analysis	R 4.1, Python 3.8.10 and 3.9.1, Aneufinder v1.20.0, scanpy v1.8.2, epiScanpy v0.3.2, anndata v0.7.8, pandas v1.3.5, matplotlib v3.5.1, scipy v1.7.1 and 1.22.0, seaborn v0.11.2 sklearn v1.0.1 and fargv v0.1.4

For manuscripts utilizing custom algorithms or software that are central to the research but not yet described in published literature, software must be made available to editors and reviewers. We strongly encourage code deposition in a community repository (e.g. GitHub). See the Nature Portfolio guidelines for submitting code & software for further information.

Data

Policy information about availability of data

All manuscripts must include a data availability statement. This statement should provide the following information, where applicable:

- Accession codes, unique identifiers, or web links for publicly available datasets
- A description of any restrictions on data availability
- For clinical datasets or third party data, please ensure that the statement adheres to our policy

The following publically available datasets analyzed in this study were downloaded: The scATAC-seq dataset for the SNU601 cell line was downloaded from the Short Read Archive (SRA) accession PRJNA674903 [https://www.ncbi.nlm.nih.gov/sra?linkname=bioproject_sra_all&from_uid=674903] and the scWGS data for the SNU601 cell line was downloaded from SRA accession PRJNA498809 [https://www.ncbi.nlm.nih.gov/bioproject/?term=PRJNA498809]; the scRNA samples for the

SNU601 cell line were downloaded from GEO under accession number GSE142750 [https://www.ncbi.nlm.nih.gov/geo/query/acc.cgi?acc=GSE142750] and the control samples from GSE150290 [https://www.ncbi.nlm.nih.gov/geo/query/acc.cgi?acc=GSE150290] (only the normal stomach) for the scRNA CNV calling. and t The two pre-treatment basal cell carcinoma samples, the PBMC and bone marrow euploid samples were obtained from SRS accession GSE129785 [https://www.ncbi.nlm.nih.gov/geo/query/acc.cgi?acc=GSE129785] (accession number GSM3722057 for SU006, and GSM3722064 for SU008, GSM3722015 for PBMC and GSM3722071 for bone marrow). The multi-ome and scATAC brain samples were downloaded from the Gene Expression Omnibus (GEO) database, series number GSE162170 [https://www.ncbi.nlm.nih.gov/geo/query/acc.cgi?acc=GSE162170] (accession numbers for the multi-ome GSM5584685, GSM5584686, GSM5584687 and for the scATAC GSM4944156, GSM4944157, GSM4944158 and GSM4944159). The pediatric glioblastoma scATAC data were downloaded from GEO, under accession numbers GSE163655 [https://www.ncbi.nlm.nih.gov/geo/query/acc.cgi?acc=GSE163655] and GSE163656 [https://www.ncbi.nlm.nih.gov/geo/query/acc.cgi?acc=GSE163656], while the matching WGS data were downloaded from the European Genome-Phenome Archive (ENA), under accession number EGAD00001005212 ESA S00001003432 [https://ega-archive.org/datasets/EGAD00001005212]. The multi-ome dataset of the COLO320HSR cell line were downloaded from the SRA accession PRJNA672109 [https://www.ncbi.nlm.nih.gov/bioproject/PRJNA672109], while the WGS of the same cell line were downloaded from the SRA accession PRJNA506071 [https://www.ncbi.nlm.nih.gov/bioproject/?term=PRJNA506071] (sample SRS4831935) and the control scRNA samples from the https://www.gutcellatlas.org/. The scWGS dataset of the HCT116 was downloaded from the European Nucleotide Archive (ENA), under accession number PRJEB27084 [https://www.ncbi.nlm.nih.gov/bioproject/?term=PRJEB27084] and the multi-ome dataset was downloaded from the SRA with accession number SRP167062 [https://trace.ncbi.nlm.nih.gov/Traces/index.html?view=study&acc=SRP136421]. For the scRNA CNV identification, as control sample was used a set of non-cancer samples downloaded from GEO under the accession GSE146771 [https://www.ncbi.nlm.nih.gov/geo/query/acc.cgi?acc=GSE146771].

Research involving human participants, their data, or biological material

Policy information about studies with human participants or human data. See also policy information about sex, gender (identity/presentation), and sexual orientation and race, ethnicity and racism.

Reporting on sex and gender	All data used for this study were publicly available.
Reporting on race, ethnicity, or other socially relevant groupings	All data used for this study were publicly available.
Population characteristics	All data used for this study were publicly available.
Recruitment	All data used for this study were publicly available.
Ethics oversight	All data used for this study were publicly available.

Note that full information on the approval of the study protocol must also be provided in the manuscript.

Field-specific reporting

Please select the one below that is the best fit for your research. If you are not sure, read the appropriate sections before making your selection.

☒ Life sciences ☐ Behavioural & social sciences ☐ Ecological, evolutionary & environmental sciences

For a reference copy of the document with all sections, see nature.com/documents/nr-reporting-summary-flat.pdf

Life sciences study design

All studies must disclose on these points even when the disclosure is negative.

Sample size	For most of the experiments described in the paper no sample size selection was made. The datasets were used in their entirety, as provided by the original publications. For the downsampling experiment, we used the downsampling function of CellRanger (see Methods) to construct unbiased sub-sized datasets from the original one. We selected to downsample several times using a different percentage of the original dataset in an step-wise manner to exhibit the robustness of the algorithm.
Data exclusions	From the SNU601 scWGS dataset a set of 134 cells were excluded from the corresponding comparison, because of their very high ploidy, that did not align with the majority of the cells. Since the cell lines were measured in different laboratories, clonal variation among them can be expected
Replication	In order to replicate the results from other studies we used the protocols provided in the relative publications. In all cases the results were successfully replicated.
Randomization	No randomization took place in the study. We used available datasets.
Blinding	No blinding took place in the study. We used available datasets.

Reporting for specific materials, systems and methods

We require information from authors about some types of materials, experimental systems and methods used in many studies. Here, indicate whether each material, system or method listed is relevant to your study. If you are not sure if a list item applies to your research, read the appropriate section before selecting a response.

Materials & experimental systems

n/a	Involved in the study
☒	☐ Antibodies
☒	☐ Eukaryotic cell lines
☒	☐ Palaeontology and archaeology
☒	☐ Animals and other organisms
☒	☐ Clinical data
☒	☐ Dual use research of concern
☒	☐ Plants

Methods

n/a	Involved in the study
☒	☐ ChIP-seq
☒	☐ Flow cytometry
☒	☐ MRI-based neuroimaging
